# SNOR promotes translation restart after dormancy

**DOI:** 10.1038/s41586-026-10530-7

**Published:** 2026-05-13

**Authors:** Maciej Gluc, Higor Rosa, Maria Bozko, Lesley A. Turner, Cassidy R. Prince, Yelena Peskova, Heather A. Feaga, Kathleen L. Gould, Simone Mattei, Ahmad Jomaa

**Affiliations:** 1 Department of Molecular Physiology and Biological Physics and Center for Cell and Membrane Physiology, University of Virginia, Charlottesville, VA USA; 2 Molecular Systems Biology Unit, European Molecular Biology Laboratory, Heidelberg, Germany; 3 Collaboration for joint PhD degree, Faculty of Biosciences, EMBL and Heidelberg University, Heidelberg, Germany; 4 Department of Cell and Developmental Biology, Vanderbilt University School of Medicine, Nashville, TN USA; 5 Department of Microbiology, Cornell University, Ithaca, NY USA; 6 EMBL Imaging Centre, European Molecular Biology Laboratory, Heidelberg, Germany; 7 Department of Biochemistry and Molecular Genetics, University of Virginia, Charlottesville, VA USA

**Keywords:** Ribosome, Cryoelectron tomography, Cryoelectron microscopy

## Abstract

Cellular dormancy enables survival during prolonged nutrient limitation by reversibly suppressing protein synthesis^1–4^. How inactive eukaryotic ribosomes are reactivated when nutrients return remains unclear. Here, using high-resolution in situ cryo-electron tomography in *Schizosaccharomyces pombe*, we identify SNOR, an SBDS domain-containing ribosome-associated factor that binds at the peptidyl transferase centre and contacts the hypusinated loop of eIF5A during glucose depletion-induced dormancy. Rather than acting as a canonical hibernation factor, SNOR licenses dormant ribosomes for rapid translational restart. Upon glucose repletion, SNOR and eIF5A act together to promote efficient recovery of polysomes and exit from dormancy. These findings define a stress-responsive ribosome restart module that couples carbon-source limitation to surveillance of the ribosomal active site and reactivation of protein synthesis.

## Abstract (web summary)

High-resolution in situ cryo-electron tomography in *Schizosaccharomyces pombe* identifies SNOR protein, which binds to ribosomes during dormancy induced by glucose depletion, priming them for rapid reactivation of protein synthesis upon glucose repletion.

## Main

Nutrient deprivation triggers metabolic stress pathways and initiates adaptive programmes to sustain cell survival. Under prolonged stress, cells can enter a reversible, non-proliferative dormant state characterized by low metabolic activity that enables them to survive extended periods of nutrient or water deprivation^1,2,4^. Dormancy is observed across diverse eukaryotic systems, including fungi, protozoa, plants, animals and even cancer cells^5–8^. In fungi, dormant cells can withstand oxidative and pH stress due to harsh environmental conditions, and in some cases, can persist in host tissues by evading the immune response for many years before reactivating^9^. Slow-growing or dormant cells formed by pathogenic fungi are also less susceptible to antifungal drugs and contribute to drug treatment failure, persistence and infection relapse^10^.

During exponential growth, yeast can devote up to 50% of cellular energy to protein synthesis. This process is rapidly downregulated under unfavourable metabolic conditions that induce cellular dormancy^3^. Ribosome shutdown is an integral part of downregulating protein synthesis and is mediated by a specialized group of proteins, known as hibernation factors^11–13^. These factors engage with critical ribosomal sites, including the transfer RNA (tRNA) binding pockets, mRNA entry channel, peptidyl transferase centre (PTC) and polypeptide exit tunnel (PET). Through this extensive interaction network, hibernation factors not only block translation but also protect ribosomes from nuclease-mediated degradation^11,14–17^. Upon return to favourable conditions, these factors dissociate or are displaced, enabling translation to resume and restoring protein synthesis^18,19^.

The first ribosome hibernation factors were discovered more than 30 years ago in bacteria, including the ribosome modulation factor (RMF) and hibernation promoting factor (HPF)^14,20,21^. Since then, functionally similar yet structurally and evolutionarily distinct proteins have also been identified in eukaryotes, particularly in the context of development, stress responses and cellular quiescence^15,16,22,23^. Despite their common function, these factors exhibit remarkable mechanistic and structural diversity. Within a single organism, multiple hibernation factors can respond to specific environmental stressors, suggesting that varied pathways for ribosome hibernation are important for prolonged survival^12,13^. Together, these discoveries point to a broader and more functionally diverse landscape of ribosome regulation than is currently known.

Recent advances in in situ cryo-electron tomography (cryo-ET) enable the structural investigation of how multiple factors act in concert to orchestrate ribosome hibernation directly within intact cells. Here we identify SNOR as a ribosome-associated factor that engages the PTC during glucose depletion-induced dormancy. Upon reintroduction of glucose, SNOR in cooperation with the universally conserved translation factor eIF5A promotes efficient translational restart. Together, these findings reveal a previously unrecognized mechanism that governs ribosome reactivation during exit from dormancy in eukaryotic cells.

## Cryo-ET reveals SNOR on hibernating ribosomes

**Fig. 1:**
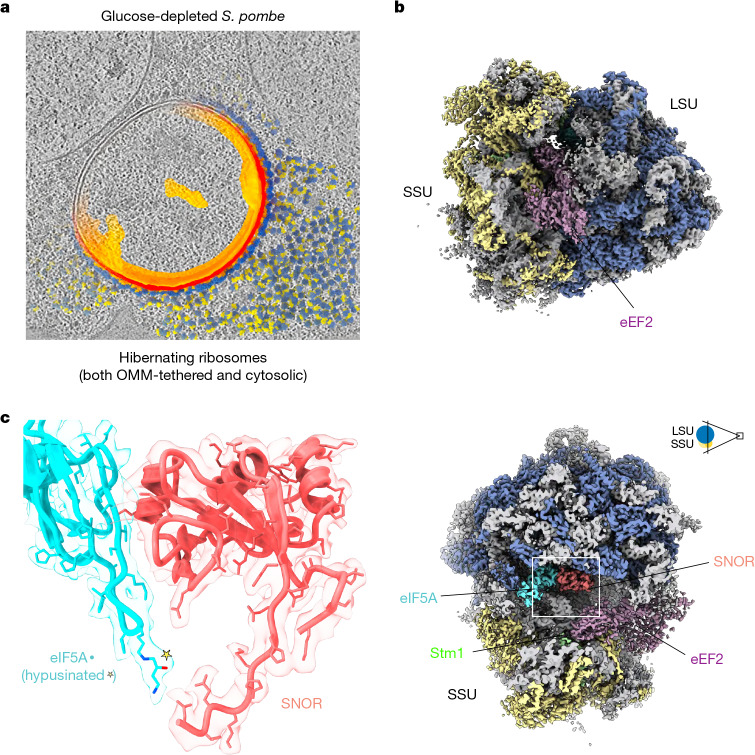
Identification of SNOR, an SBDS domain-containing factor bound to hibernating ribosomes, by in situ cryo-ET. **a**, Computational slice through a tomogram of a mitochondrion decorated with ribosomes from an *S. pombe* cell grown for 7 days in EMM containing 0.5% (w/v) glucose. Partial segmentation highlights the mitochondrion and surrounding ribosomes. The ribosomal small subunit (SSU) and large subunit (LSU) are shown in yellow and blue, respectively, and the outer mitochondrial membrane (OMM) and inner mitochondrial membrane are shown in dark and light orange, respectively. **b**, Cryo-ET reconstruction of the hibernating ribosome at 3.38 Å resolution, shown as a surface. Ribosomal proteins of the SSU and LSU are coloured yellow and blue, respectively. **c**, Close-up view of the SNOR–eIF5A interface with the corresponding electron microscopy density shown as a semi-transparent surface.

Our previous cellular cryo-ET analysis of glucose-depleted *S. pombe* dormant cells revealed a population of outer mitochondrial membrane (OMM)–associated ribosomes (Fig. 1a). These ribosomes were trapped in a hibernating state, bound by elongation factor eEF2 and devoid of tRNA^24^. Additional electron densities were apparent at the ribosomal subunit interface within the tRNA-binding sites, suggesting that dormant ribosomes might include previously unrecognized regulatory components. However, the limited resolution of the initial reconstruction (around 11 Å) precluded molecular identification of these features.

**Extended Data Fig. 1:**
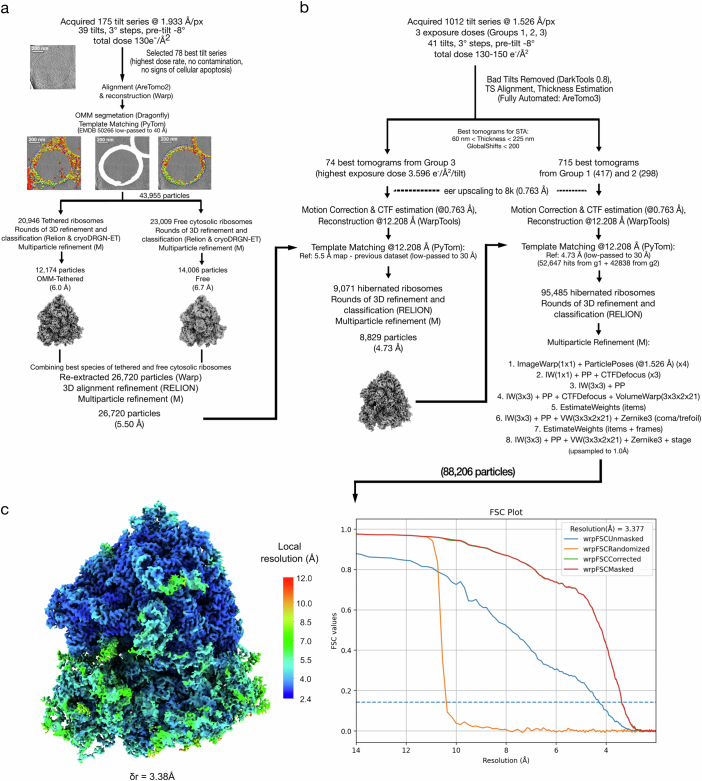
Cryo-ET data processing and resolution assessment of hibernating 80S ribosomes. (a) Cryo–electron tomography (cryo-ET) initial dataset. 175 tilt series were collected and processed. OMM-tethered and free-cytosolic ribosomes were selected by segmenting mitochondrial membranes and manual curation. Maps of each were later reconstructed to check for the presence of hibernation factors. Once these factors were found in both, the particles were combined, reaching a 5.50 Å map. (b) A second dataset of 1,012 tilt series was acquired at a pixel size of 1.526 Å, using three exposure dose groups and a total dose of 130–150 e–/Å². Tilt series alignment, thickness estimation, and quality filtering were performed in a fully automated manner using AreTomo3. After removal of poor-quality tilt series, the best tomograms were selected based on thickness (<225 nm) and global shift criteria. Tomograms from the highest-dose group (Group 3; 74 tomograms) and from Groups 1 and 2 (715 tomograms total) were processed separately. Motion correction, CTF estimation (0.763 Å), and tomogram reconstruction (12.208 Å) were performed using WarpTools. Template matching was carried out in PyTom using low-pass filtered reference maps, yielding 9,071 and 95,485 hibernating ribosomes, respectively. Particles were subjected to rounds of 3D refinement and classification in RELION, followed by multiparticle refinement in M, including iterative image warping, particle pose refinement, CTF defocus estimation, volume warping, Zernike polynomial refinement, and weight estimation, with final maps upsampled to 1.0 Å. (c) A local resolution map of the final reconstruction is shown, colored according to local resolution (Å). At the right, Gold-standard Fourier shell correlation (FSC) curves calculated from independently refined half-maps using masked and unmasked reconstructions. The final overall resolution is 3.38 Å at the FSC = 0.143 criterion.

**Extended Data Fig. 2:**
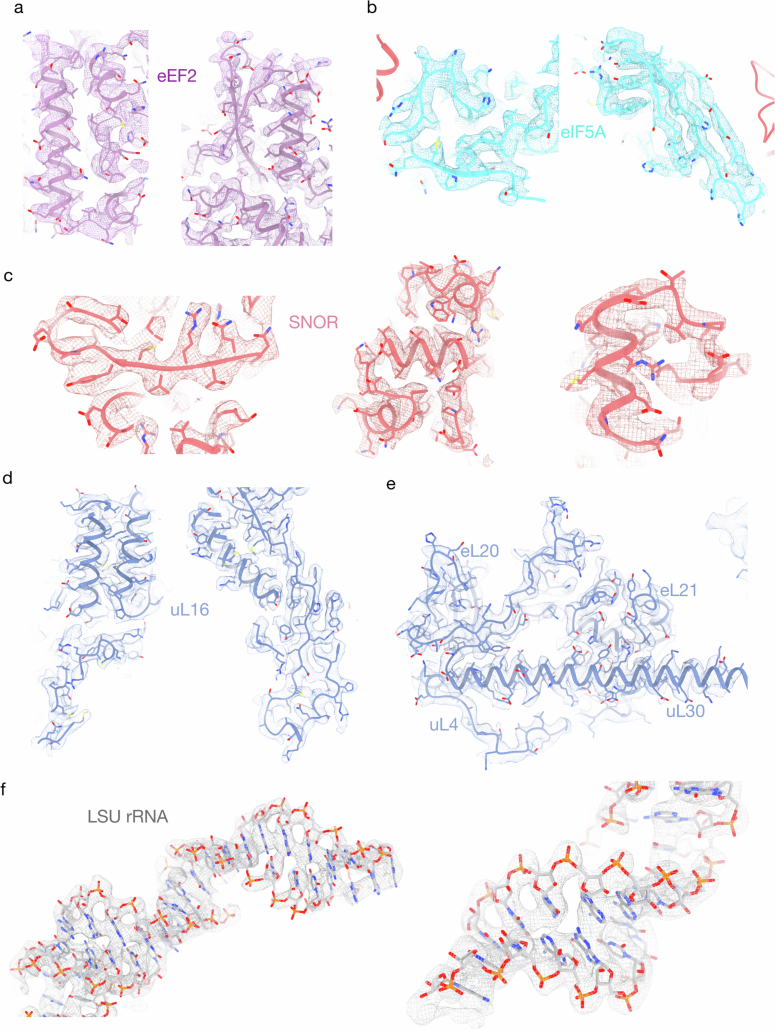
Representative cryo-ET densities for ribosomal proteins, factors, and rRNA. Representative cryo-ET densities illustrating map quality and agreement with the atomic model of the hibernating *S. pombe* ribosome, highlighting regions corresponding to eEF2 (a, purple), eIF5A (b, teal), SNOR (c, coral), ribosomal protein uL16 (d, blue), ribosomal proteins uL4, eL20, eL21, and uL30 (e, blue), and rRNA (f, gray). Atomic models are shown as cartoons for proteins and sticks for rRNA, with maps displayed as meshes. All renderings were generated in ChimeraX.

To address this question, we undertook a large-scale in situ cryo-ET acquisition strategy, combining optimized cryogenic focused ion beam (cryo-FIB) lamella preparation with extensive subtomogram averaging (Extended Data Fig. 1 and Methods). Subclassification of OMM-associated and cytosolic ribosomes using an initial dataset revealed a shared hibernation architecture and no structural differences in ribosome-bound factors (Supplementary Fig. 2). We therefore expanded data acquisition across the entire cell to maximize particle numbers and resolution. In total, 1,012 tilt series were collected from 26 lamellae, yielding nearly 100,000 high-quality ribosomal particles. This effort resulted in a 3.38 Å in situ reconstruction of the hibernating ribosome, with local resolution reaching 2.8 Å in the best-resolved regions, enabling unambiguous side-chain assignment of ribosome-associated factors at the subunit interface (Fig. 1a,b, Extended Data Fig. 2 and Extended Data Table 1).

At this resolution, the molecular architecture of the dormant ribosome can be defined at near-atomic detail. In addition to eEF2^24^ and the hibernation factor Oga1 (the orthologue of *Saccharomyces cerevisiae* Stm1)^25^, we resolved density corresponding to the initiation factor eIF5A, including its hypusinated lysine (Hyp52) (Fig. 1b,c and Extended Data Fig. 2), a modification that is critical for its activity^26^. Of note, we identified an additional electron density with clearly resolved side chains corresponding to a small globular domain positioned between eEF2 and eIF5A, adjacent to RNA helix 69 (H69) and capping the PTC.

**Extended Data Fig. 3:**
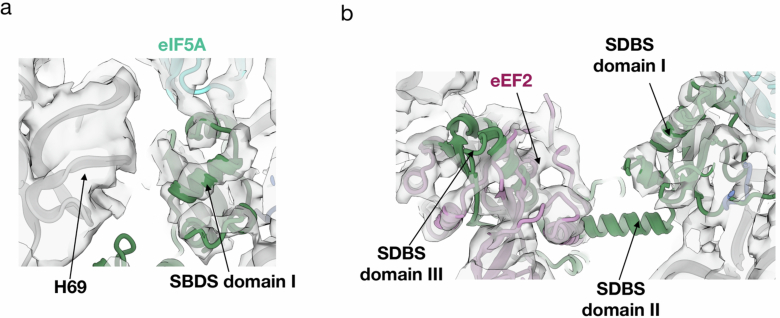
Domains I but not Domain II or III of Sdo1/SBDS fit into the EM-density of the hibernating ribosomes. (a) Fit of SBDS domain I (green) into the cryo-EM density of the *S. pombe* hibernating ribosome. Ribosomal RNA is shown in gray, with helix 69 indicated by an arrow; eIF5A is shown in teal. The electron density map is displayed as a semi-transparent surface. (b) Absence of observable density for SBDS domain II and predicted steric clash between SBDS domain III and eEF2 (purple). The positions of SBDS domains II and III are indicated by arrows. The electron density map is shown as a semi-transparent surface. Electron density maps filtered to 7 Å for visualization.

Based on its location and overall fold, we compared this density with known ribosome-associated factors. The closest structural resemblance was to the N-terminal SBDS domain (domain I) of Sdo1 in yeast^27^ (SBDS in humans^28^). However, this assignment was inconsistent with the resolved side-chain features, and domains II and III were neither supported by density nor structurally compatible, as they would sterically clash with eEF2 (Extended Data Fig. 3).

To identify the factor in an unbiased manner, we performed structure-based searches against protein structure databases using Foldseek^29^. This analysis identified Rtc3 (SPBC21C3.19), an SBDS domain-containing protein of previously unknown function^30–32^ as the closest structural homologue. An AlphaFold model of Rtc3 docked into the in situ cryo-ET map with excellent agreement and minimal adjustment, accounting for nearly all resolved side-chain features and providing strong structural evidence for its identity. Consistently, mass spectrometry analysis confirmed the presence of Rtc3 in ribosomes isolated after prolonged glucose depletion (Supplementary Table 1; available on Figshare: 10.6084/m9.figshare.31350286 (ref. ^33^)). Based on its SBDS fold and newly defined role in cellular dormancy, we designate this factor the SBDS domain-containing hibernation factor (SNOR) (Fig. 1c).

## SNOR expression increases during glucose stress

**Fig. 2:**
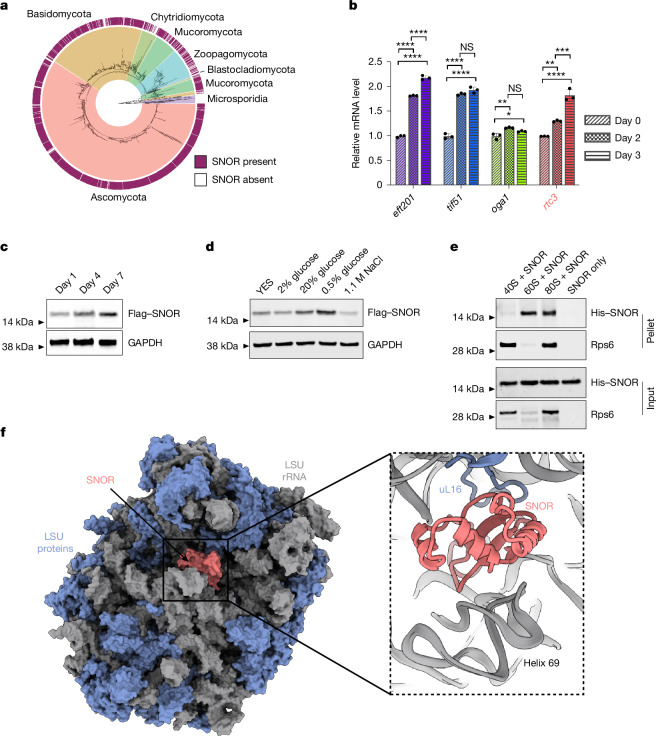
SNOR expression increases during glucose depletion and SNOR binds ribosomes. **a**, Maximum-likelihood phylogeny based on 18S rRNA from 2,248 fungal genomes showing the distribution of SNOR. The accompanying heat map indicates detection of SNOR in each genome. **b**, Normalized relative mRNA levels of *eft201* (encoding eEF2), *tif51* (eIF5A), *oga1* (Stm1) and *rtc3* (SNOR) at days 0 (diagonal stripes), 2 (checkered) and 3 (horizontal stripes) of glucose depletion (*n* = 3 biologically independent samples). Data are mean ± s.d. One-way ANOVA. **c**, Immunoblot analysis of SNOR protein in *S. pombe* cell lysates collected after 1, 4 and 7 days of glucose depletion. SNOR was detected using a Flag antibody; GAPDH served as a loading control. Representative immunoblots from biological replicate experiments are shown. Uncropped gels can be found in Supplementary Fig. 1. **d**, Immunoblot analysis of SNOR protein in *S. pombe* cell lysates exposed to the indicated stress conditions for 2 h prior to lysis. SNOR was detected using a Flag antibody; GAPDH served as a loading control. Representative immunoblots from biological replicate experiments are shown. YES, yeast extract with supplements. **e**, Ribosome co-sedimentation assays showing SNOR association with purified 40S, 60S and 80S ribosomal particles. SNOR was detected via His tag; Rps6 served as a ribosomal loading control. Representative immunoblots from biological replicate experiments are shown. **f**, Cryo-EM structure of the in vitro reconstituted 60S–SNOR complex. Ribosomal proteins are shown in blue and rRNA is in grey. Inset shows a close-up view of the atomic model, with SNOR bound at the PTC of the ribosome. *P < 0.05, **P < 0.01, ***P < 0.001, ****P < 0.0001; NS, not significant (*P* ≥ 0.05).

We first explored how conserved SNOR is across fungi. To assess its distribution across fungal species, we searched 2,248 representative fungal genomes from the NCBI RefSeq database using SNOR sequences similar to the protein characterized in this study. SNOR was detected in 87% of surveyed fungi and was predominantly found in Ascomycota, Basidiomycota and Mucoromycota (94%, 87% and 85% of annotated genomes, respectively) (Fig. 2a and Supplementary Tables 2 and 3). Most genomes surveyed encoded a single SNOR homologue, although a few contained up to five copies. Conversely, SNOR was entirely absent in Microsporidia, which are known for their highly compact genomes resulting from extreme genome reduction associated with obligate intracellular parasitism^34^. We also found several hits in plants, insects, fish and in some mammals that were annotated as an SBDS N-terminal domain-containing protein. However, they were only detected at the transcript level and contain low sequence homology to SNOR (below 30%), therefore we excluded these hits from our final search criteria.

Next, we investigated SNOR expression levels during glucose depletion and other stress-related conditions associated with nutrient deprivation. We performed quantitative PCR with reverse transcription (RT-qPCR) to monitor changes in mRNA levels during progressive glucose depletion. Samples were collected on days 0, 2 and 3 of the glucose depletion course in Edinburgh minimal medium (EMM) containing 0.5% glucose, based on prior observations showing that global protein synthesis is markedly repressed by day 4 (ref. ^24^). Expression of *eft201*,* tif51* and *rtc3* transcripts, encoding eEF2, eIF5A and SNOR, respectively, was increased after 3 days of glucose deprivation, with *eft201* and *tif5*1 transcripts showing the strongest induction, whereas *oga1* (Stm1) transcript levels remained largely unchanged (Fig. 2b and Supplementary Table 4). To assess whether SNOR protein levels were similarly upregulated, we used CRISPR–Cas9 to introduce a 2× Flag epitope at the endogenous SNOR locus, generating an N-terminally tagged SNOR protein. Immunoblotting with anti-Flag over a 7-day glucose depletion course in EMM containing 0.5% glucose showed a significant increase in SNOR protein levels by days 4 and 7 of glucose deprivation (Fig. 2c).

To determine whether SNOR induction reflects carbon stress rather than a general stress response, we examined SNOR expression under a range of nutrient and osmotic conditions. Immunoblot analysis showed that SNOR was selectively upregulated under limiting (0.5% w/v) and high (20% w/v) glucose conditions, but not under standard glucose levels (2%). SNOR expression was not induced by complete glucose starvation (0%), amino acid or nitrogen deprivation, or by osmotic stress induced by 1.1 M NaCl (Fig. 2d and Supplementary Fig. 3). Together, these data indicate that SNOR induction is specific to glucose-dependent carbon stress and is not a consequence of general nutrient or osmotic stress.

## Structure of the 60S–SNOR complex

To determine whether SNOR directly associates with the ribosome in vitro, we recombinantly expressed and purified SNOR from *Escherichia coli* and conducted binding assays using purified *S. pombe* ribosomal subunits. SNOR was incubated with either 80S ribosomes, 60S large subunits or 40S small subunits, followed by ribosome co-sedimentation using sucrose cushion ultracentrifugation. The binding assays showed that SNOR specifically interacts with the 60S large subunit and intact 80S ribosomes (Fig. 2e). To further examine how SNOR engages the ribosome and describe the molecular interactions responsible for stabilizing its binding to ribosomal RNA (rRNA), we determined the structure of the in vitro reconstituted complex between the 60S ribosomal subunit and SNOR (60S–SNOR complex) using single-particle cryo-electron microscopy (SPA cryo-EM). Given that SNOR binds both 60S and 80S ribosomes, we focused on the 60S subunit to minimize sample heterogeneity arising from intersubunit rotational dynamics, thereby facilitating cryo-EM image processing.

**Extended Data Fig. 4:**
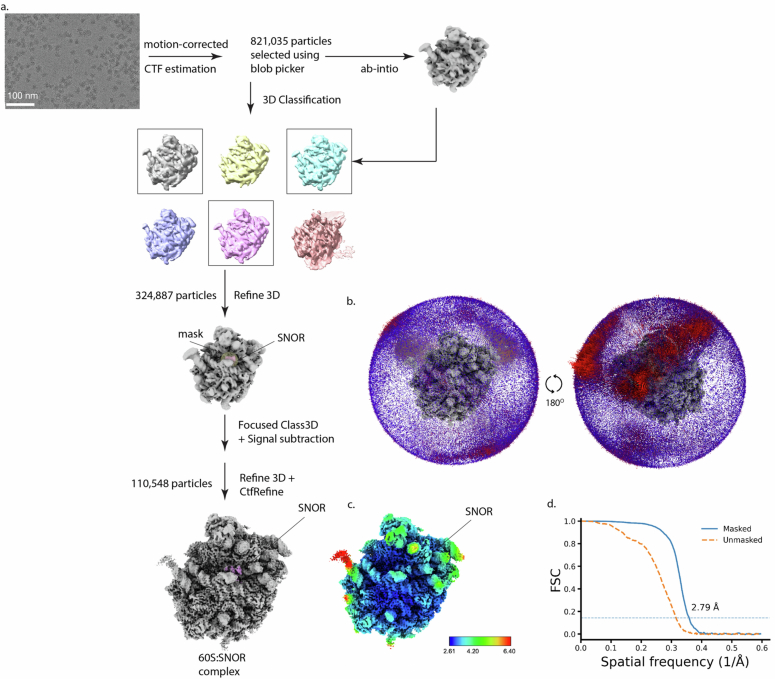
Processing scheme of the single-particle analysis of 60S:SNOR complex. (a) Cryo-EM data processing workflow. Motion-corrected micrographs were first subjected to CTF estimation, and particles were then picked using Laplacian of Gaussian (LoG) picker in RELION, yielding 821,035 particles. Following ab initio reconstruction and an initial round of 3D classification, particles from 60S classes exhibiting SNOR density were selected, resulting in 324,887 particles for initial 3D refinement. A soft mask encompassing the SNOR density was then applied for two rounds of focused 3D classification with signal subtraction in RELION3. Final 3D refinement and CTF refinement of 110,548 particles displaying high-resolution SNOR features yielded the final 60S–SNOR reconstruction. (b) Angular distribution plots of particle orientations for the final reconstruction, shown in two views rotated by 180°. (c) Local resolution map of the final 60S–SNOR reconstruction, colored according to local resolution (Å). (d) Gold-standard Fourier shell correlation (FSC) curves for masked (solid blue) and unmasked (dashed orange) reconstructions.

**Extended Data Fig. 5:**
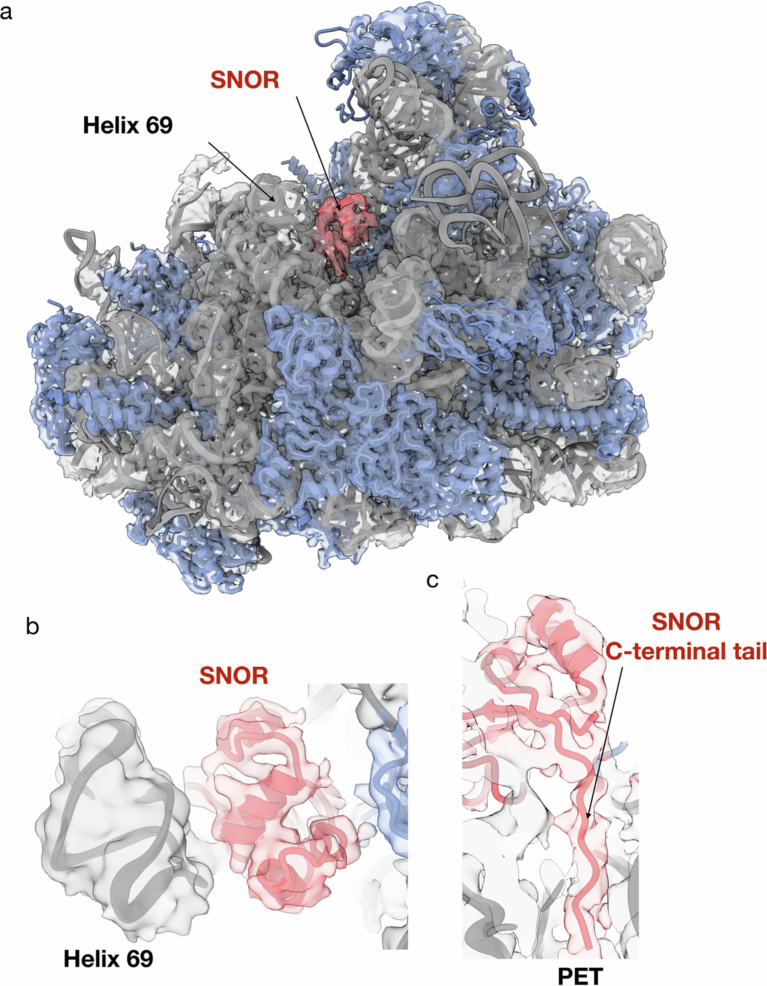
Atomic model of SNOR bound to the ribosomal large subunit. (a) Overall fit of the 60S:SNOR atomic model into the cryo-EM density map, shown as a semi-transparent surface filtered to 3.5 Å. Ribosomal RNA is shown in gray, ribosomal proteins in blue, and SNOR in coral. RNA helix 69 and the SNOR binding site are indicated by arrows. (b) Top-down close-up view showing the fit of SNOR within the observed density at the PTC, adjacent to helix 69. (c) Close-up view of the SNOR C-terminal tail inserted into the PET.

**Extended Data Fig. 6:**
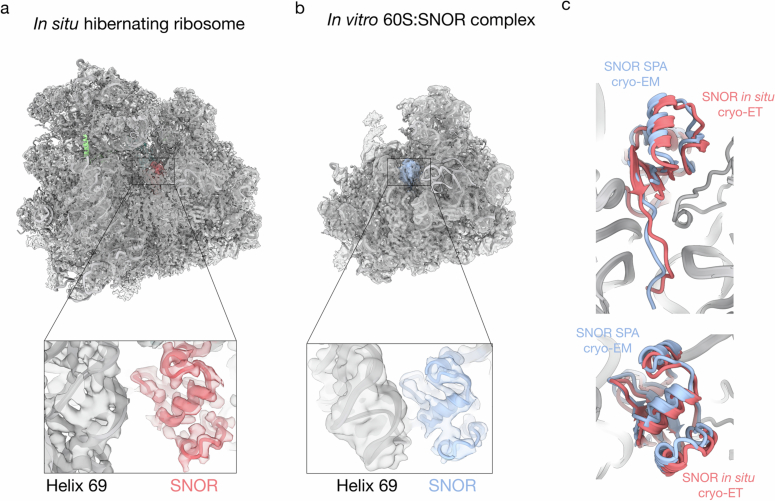
Comparison of SNOR binding position between in situ hibernating ribosome and in vitro reconstituted 60S:SNOR complex atomic models. (a) Overall fit of the atomic model of the *S. pombe* hibernating ribosome bound to eEF2 (purple), eIF5A (teal), Oga1 (Stm1, green) and SNOR (coral). Inset shows close-up of SNOR binding position in the PTC near helix 69 of the large ribosomal subunit RNA, density corresponding to SNOR colored coral. (b) Overall fit of the atomic model of in vitro reconstituted complex of the large ribosomal subunit and SNOR (sky blue). Inset shows close-up of SNOR binding position in the PTC near helix 69 of the large ribosomal subunit RNA, density corresponding to SNOR colored sky blue. (c,d) Overlay of atomic models of SNOR obtained from the in situ cryo-ET (SNOR colored coral) and in vitro SPA cryo-EM (SNOR colored sky blue) reconstructions. In all renderings the atomic models are shown as cartoon and the maps shown as semi-transparent surface. All images were produced in ChimeraX.

The final structure of the 60S–SNOR complex was resolved at a resolution of 2.8–3.5 Å (Extended Data Fig. 4 and Extended Data Table 1). The SPA cryo-EM map of the in vitro reconstituted complex confirmed that SNOR binds to the PTC of the 60S subunit at the same active site location that is observed in the in situ cryo-ET structure of the hibernating ribosome (Fig. 2f and Extended Data Figs. 5 and 6), independently confirming the location of SNOR binding on dormant ribosomes.

## SNOR–rRNA interactions drive ribosome binding

**Fig. 3:**
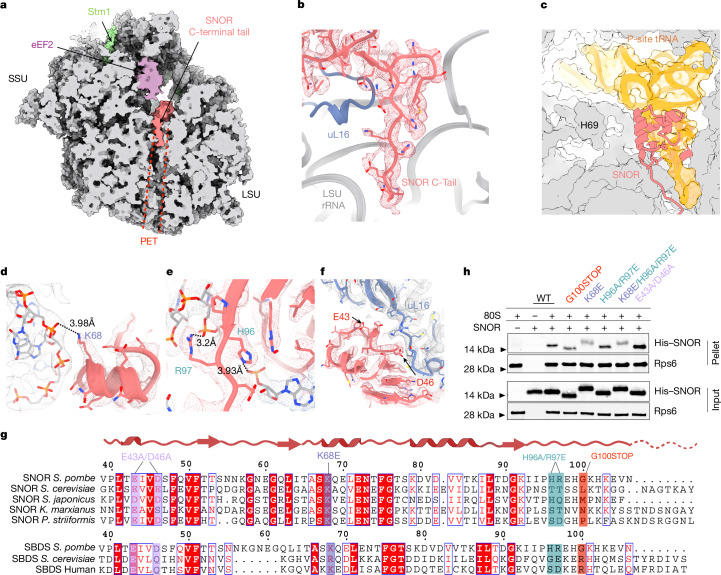
Structural and mutational analysis of the SNOR-bound hibernating ribosome. **a**, Slab view of the *S. pombe* hibernating ribosome atomic model shown as a surface, highlighting insertion of the C-terminal tail of SNOR into the PET (dashed lines). Ribosomal RNA and proteins are shown in grey. **b**, Close-up view of the PET showing electron microscopy density with resolved side chains corresponding to the C-terminal tail of SNOR inserted into the tunnel. **c**, Structural comparison of SNOR occupancy with the P-site tRNA binding site (Protein Data Bank (PDB): 9AXV). Ribosomal protein and RNA are shown as grey surface; SNOR is depicted as coral cartoon; and the P-site tRNA is shown in orange. **d**,**e**, Close-up views of SNOR residues K68 (**d**) and H96 and R97 (**e**) contacting rRNA. **f**, Close-up view of SNOR residues E43 and E46 contacting ribosomal protein uL16 (blue). **g**, Multiple sequence alignment of *S. pombe* SNOR with homologues from four fungal species (top; *S. cerevisiae*, *Schizosaccharomyces japonicus*,* Kluyveromyces marxianus* and *Puccinia striiformis*) and with *S. cerevisiae* Sdo1 and human SBDS (bottom), highlighting conserved ribosome-contacting residues: E43 and E46 (lilac), K68 (purple), H96 and R97 (teal), and G100 (coral), which was selected for C-terminal truncation. Alignment was generated using ESPript 3.0. **h**, Immunoblot analysis of ribosome co-sedimentation assays showing interaction of wild-type (WT) SNOR and SNOR mutants (G100STOP, K68E, H96A/R97E, K68E/H96A/R97E and E43A/D46A) with purified *S. pombe* 80S ribosomes. SNOR was detected via His tag; Rps6 served as a loading control. Representative immunoblots from biological replicate experiments are shown.

**Extended Data Fig. 7:**
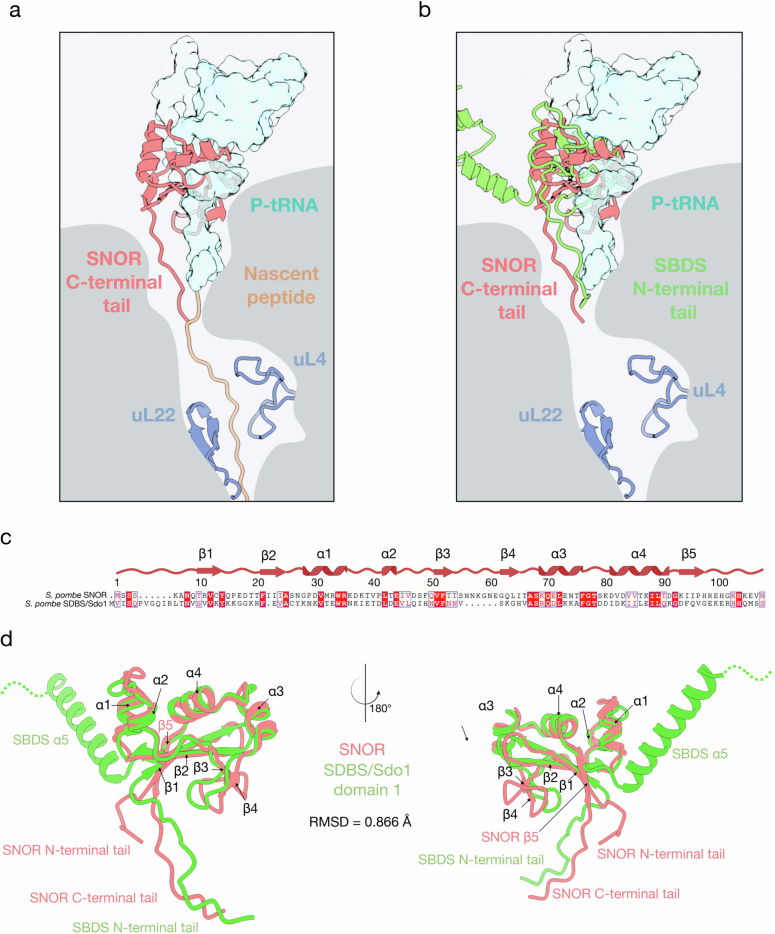
Structural comparison of SNOR and SBDS binding in the PET valley. (a) SNOR occupies the site of the P-site tRNA, with its C-terminal tail extending into the path of the nascent peptide (orange). (b) SNOR binding position overlaps with that of SBDS domain I (PDB: 6QKL); however, SBDS inserts its N-terminal tail into the PET (green). The P-site tRNA is shown as a light blue surface, and ribosomal proteins uL22 and uL4 are depicted as blue cartoons. (c) Schematic representation of SNOR secondary structure elements (top). β-sheets represented as arrows and symbol “β”. Helices represented as ribbons and symbol “α”. Sequence alignment of *S. pombe* SNOR with *S. pombe* SBDS/Sdo1 (bottom). Alignment generated using ESPript 3.0. (d) Structural comparison of a superimposed AlphaFold model of *S. pombe* “SBDS domain” of Sdo1 (human SBDS homolog) (green) and SNOR (coral) atomic model.

SNOR binds within the canyon formed by the ribosomal tRNA-binding sites, capping the entrance to the PET (Fig. 3a–c and Extended Data Fig. 7a,b). SNOR interacts almost exclusively with rRNA through charged interactions, with the side chains of K68, H96 and R97 were resolved, making contacts with the rRNA phosphate backbone (Fig. 3d,e).

In addition to its interactions with rRNA, SNOR establishes multiple contacts with the ribosomal protein uL16 (Rpl10) (Fig. 3f). Notably, uL16 contains a flexible loop that is disordered in the empty ribosome but adopts an ordered conformation when specific factors such as tRNA or Sdo1 engage the ribosome^28,35^. This loop extends toward the P-site and interacts with the CCA end of the P-site tRNA during translation^35^. In the current structure, however, this loop establishes contacts with a loop located between alpha helix 2 and beta sheet 3 of SNOR (Fig. 3f). In particular, residues E43 and D46 of SNOR form polar interactions with several residues within this loop of uL16.

To assess the conservation of these critical residues contacting the ribosome, we performed a multiple sequence alignment of SNOR across various yeast species, also including Sdo1 and human SBDS proteins for further comparison (Fig. 3g). Most of the residues that interacted with the rRNA and proteins were conserved in yeast. In particular, K68 showed strong conservation, including with human SBDS, which contains two lysine residues in this region, one of which has been reported as a disease-associated K/E variant^36^. To then evaluate the functional relevance of these interactions, we generated a series of SNOR mutants and tested their binding to the ribosome. First, we probed SNOR interactions with the rRNA. We generated a single mutant (K68E), a double mutant (H96A/R97E) and a triple mutant (K68E/H96A/R97E). We assessed ribosome-binding activity using in vitro co-sedimentation assays followed by immunoblot analysis (Fig. 3h). The K68E single mutant and K68E/H96A/R97E triple mutant both exhibited reduced ribosome binding relative to wild-type SNOR, whereas the H96A/R97E double mutant demonstrated no significant change in its binding to the ribosome. These results suggest that highly conserved K68 is the primary contributor to RNA-mediated SNOR binding to the ribosome.

The C-terminal tail of SNOR, which is composed of seven residues enriched in positively charged amino acids, was observed inserting into the PET (Fig. 3b). This mode of interaction contrasts with that of Sdo1, which inserts its N-terminal domain into the PET^28^ (Extended Data Fig. 7b,c), despite it having a similar SBDS domain (Extended Data Fig. 7c,d). To test the importance of this tail, we generated a truncation mutant lacking the C-terminal tail (G100STOP) to assess the role of PET insertion. The C-terminal truncation had also reduced ribosome binding, albeit to a lesser extent than the K68E mutant. This indicates that PET insertion is not required for association but may serve another role, perhaps in sensing nascent peptide presence within the tunnel. Finally, a double mutant (E43A/D46A) was created to disrupt interactions with uL16. Notably, this mutant displayed enhanced binding to the ribosome, possibly due to the removal of negatively charged residues that might otherwise repel the surrounding rRNA.

## SNOR forms a tripartite complex

**Fig. 4:**
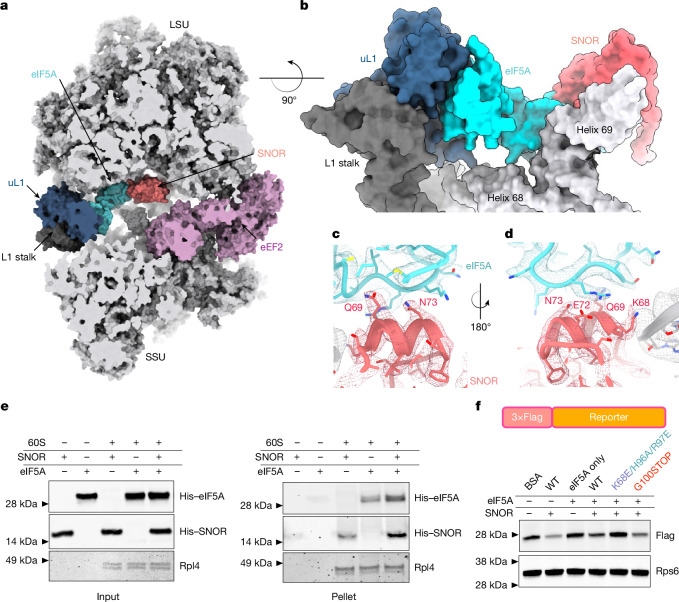
A SNOR–eIF5A–uL1 tripartite interface stabilizes the L1 stalk and helix H69 in hibernating ribosomes. **a**, Atomic model of the *S. pombe* hibernating ribosome shown as a surface representation. Ribosomal RNA is shown in grey. **b**, Close-up view of the SNOR–eIF5A–uL1 tripartite interface stabilizing the L1 stalk. rRNA helices H68 and H69 in grey. **c**,**d**, Close-up views of the SNOR–eIF5A interaction interfaces, highlighting SNOR residues S54/N55/N56 (**c**) and E70/N73 (**d**) engaging eIF5A, with corresponding cryo-EM densities overlaid as mesh. **e**, Immunoblot analysis of ribosome co-sedimentation assays demonstrating cooperative co-binding of SNOR and eIF5A to ribosomes. SNOR was detected via His tag; Rpl4 served as a loading control. Representative immunoblots from biological replicate experiments are shown. **f**, In vitro translation assays using RRL, comparing Flag-tagged reporter expression in the presence of wild-type SNOR, SNOR mutants and/or eIF5A, relative to a BSA control. Representative immunoblots from biological replicate experiments are shown.

**Extended Data Fig. 8:**
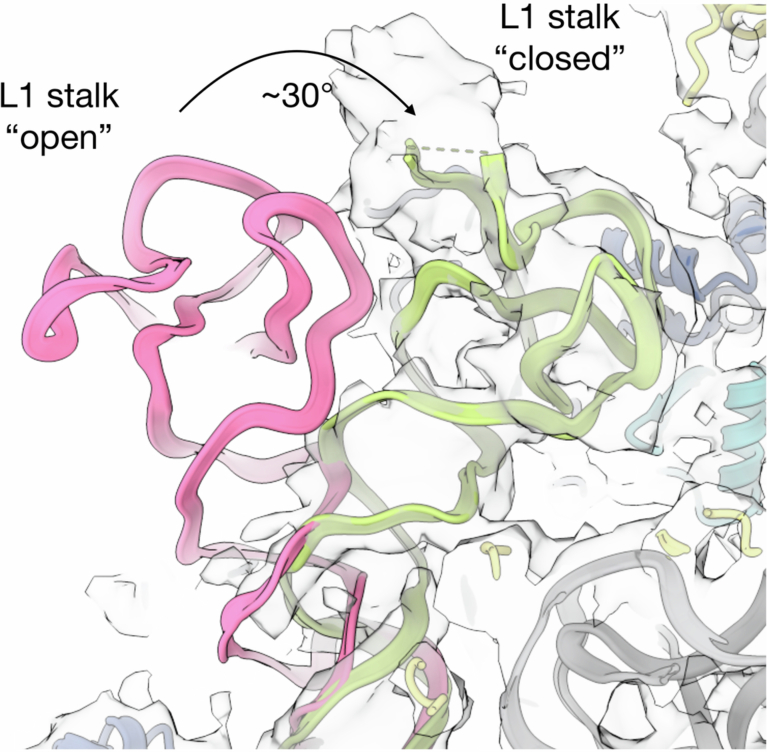
L1 stalk shift into closed state on hibernating ribosome. Conformational comparison of the L1 stalk in “open” (pink, PDB: 6WOO) versus “closed” (green, this study) states. The ribosome atomic model is shown as cartoon; the cryo-ET map shown as a semi-transparent surface.

SNOR contacts the highly conserved factor eIF5A in the vicinity of the PTC, whereas eIF5A simultaneously engages with the ribosomal protein uL1, forming a tripartite interface (Fig. 4a,b). eIF5A engagement with uL1 stabilizes the L1 stalk in an inward (closed) position (Extended Data Fig. 8). The uL1–eIF5A–SNOR tripartite interface may act as a wedge that locks interactions between H69, the PTC and the L1 stalk. This configuration could help maintain a hibernation-like state by preventing L1-tRNA interactions that normally occur during the elongation cycle^37^. The position occupied by SNOR also overlaps with the inward-shifted conformation of helix H69 that was previously observed in inactive ribosomes isolated under similar conditions^24^, indicating that SNOR binding may stabilize the conformation of this helix, sterically occluding this inactive H69 configuration (Supplementary Fig. 4).

SNOR establishes contacts with eIF5A through a conserved interface involving residues Q69, E72 and N73, which form polar interactions with eIF5A (Fig. 4c,d). To test whether SNOR and eIF5A influence each other’s ribosome association, we performed co-sedimentation assays using purified 60S subunits together with SNOR and eIF5A (Fig. 4e). When incubated individually, each factor co-pelleted with the 60S subunit; however, when added together, recovery of both SNOR and eIF5A in the ribosomal pellet increased compared with either factor alone. This enhanced co-pelleting is consistent with cooperative binding between SNOR and eIF5A mediated by this interface.

To assess whether SNOR can inhibit translation, we used an in vitro translation system using rabbit reticulocyte lysate (RRL) and monitored protein synthesis using a Flag-tagged nascent chain construct. We confirmed that SNOR also associates with mammalian ribosomes using in vitro binding assays with purified human ribosomes from HEK cells, followed by co-sedimentation through a sucrose cushion (Supplementary Fig. 5). We then performed in vitro translation in RRL in the presence or absence of SNOR. The addition of purified SNOR to the RRL reaction significantly reduced levels of Flag reporter compared with the bovine serum albumin (BSA) control (Fig. 4f).

We then tested whether eIF5A further enhances SNOR-dependent repression of translation. The addition of eIF5A alone into RRL showed increased levels of Flag reporter (consistent with its role for increasing translation efficiency^35^), which was reduced by the addition of SNOR. By contrast, the SNOR triple mutant (K68E/H96A/R97E), which exhibits poor binding to ribosomes, did not repress translation, whereas the tail mutant (G100STOP) showed a partial reduction in reporter synthesis, consistent with its weaker ribosome binding (Fig. 4f).

## SNOR and eIF5A promote translation restart

**Fig. 5:**
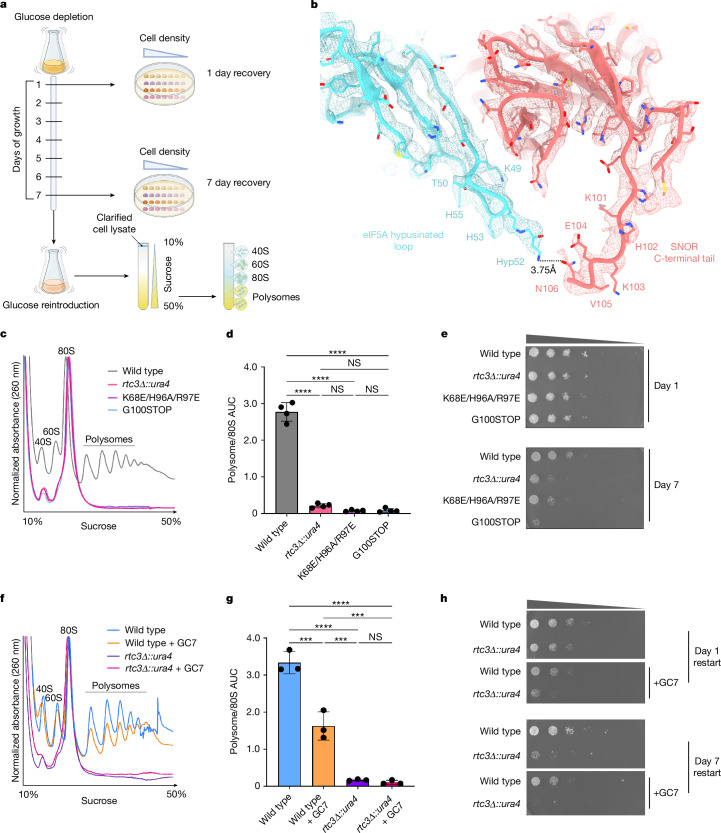
SNOR and hypusinated eIF5A are required for efficient translational restart and cell viability following glucose reintroduction. **a**, Schematic of the experimental design used to assess cell viability and protein synthesis restart following glucose reintroduction. Created in BioRender; Gluc, M. https://BioRender.com/w6c25o4 (2026). **b**, Close-up view of cryo-ET density highlighting eIF5A and SNOR, shown as mesh. The interaction between the hypusinated lysine residue (Hyp52) of eIF5A and Asn106 in the C-terminal tail of SNOR is indicated. Atomic models are displayed as sticks and cartoons. **c**, Overlay of representative polysome gradient profiles from SNOR wild type, SNOR-knockout (*rtc3Δ::ura4*), SNOR knock-in ribosome-binding-deficient (K68E/H96A/R97E) and SNOR C-terminal tail deletion (G100STOP) *S. pombe* cells after 2 h of glucose reintroduction following 7 days of glucose depletion. **d**, Relative area under the curve (AUC) corresponding to 80S monosomes from **c**. Data are mean ± s.d (*n* = 4 biological replicates). Two-tailed *t*-test. **e**, Serial dilution spot assays of cultures grown at 32 °C in EMM containing 0.5% glucose. Tenfold serial dilutions of normalized samples (OD_600_ = 0.25) were plated on EMM containing 2% glucose and incubated at 32 °C. Day 1 corresponds to 24 h of continuous incubation. **f**, Overlay of representative polysome gradient profiles from wild-type, wild-type plus GC7, SNOR-knockout, and SNOR-knockout plus GC7 *S. pombe* performed as in **c**. **g**, Relative area under the curve corresponding to 80S monosomes from **f**. Data are mean ± s.d (*n* = 3 biologically independent experiments). Two-tailed *t*-test. **h**, Serial dilution spot assays performed as in **d**, comparing wild-type and SNOR-knockout cells in the presence or absence of GC7 following glucose reintroduction.

Next, we hypothesized that similarly to Sdo1, SNOR might have an additional role in restarting translation, but in the context of exit from cellular dormancy. To test this model, we monitored translation shutdown and recovery following glucose depletion and assessed whether the interaction between SNOR and the hypusinated loop of eIF5A is required for this process (Fig. 5a,b). Previously, we showed that *S. pombe* cells grown in EMM with low glucose (0.5% w/v) stop dividing and shut down protein synthesis after 3–4 days, once glucose is exhausted, as indicated by the loss of polysomes in sucrose gradients and transition to dormancy by day 7 (ref. ^24^).

**Extended Data Fig. 9:**
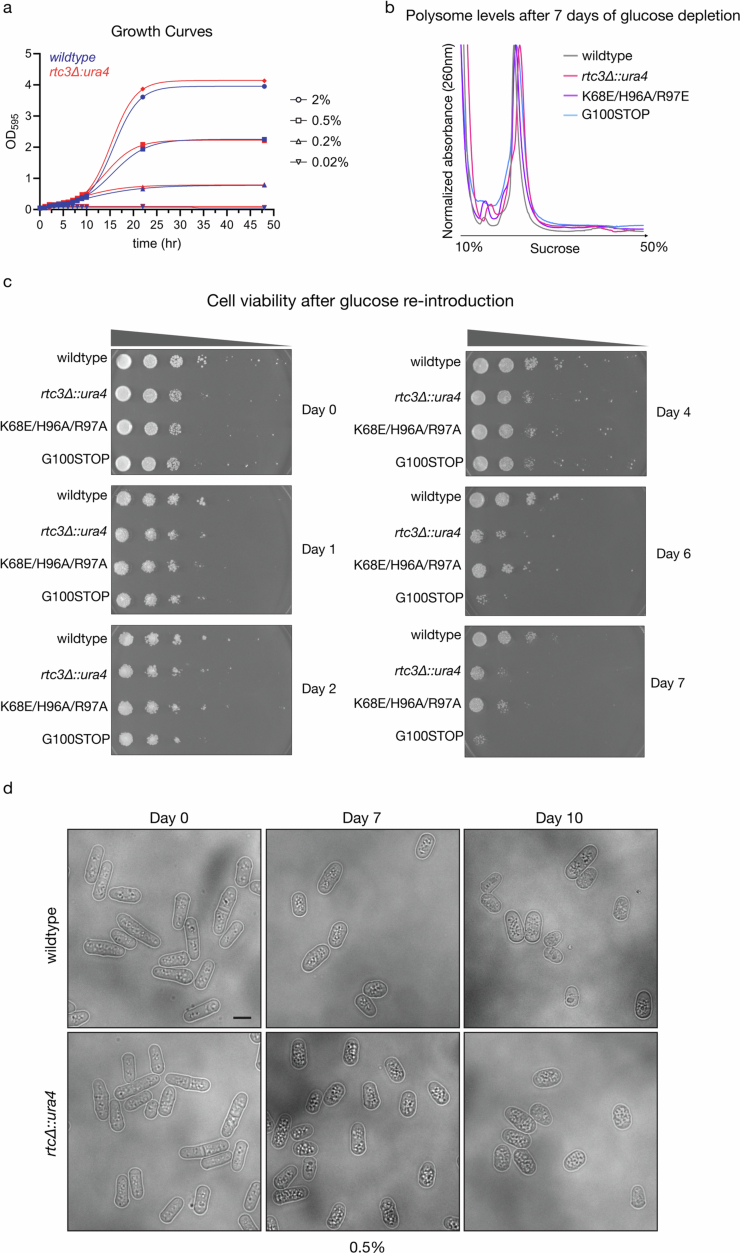
(a) Growth curves of wildtype and *rtc3∆* cultures in EMM with different glucose levels at 32 °C. (b) Polysome gradient profiles from *S. pombe* wildtype and *rtc3* mutant strains following 7 days of glucose depletion showing cessation of translation and accumulation of monosomes. Profiles from wildtype cells are shown in black, profiles from *rtc3∆* cells are shown in pin, profiles from *rtc3* K68E/H96A/R97E cells shown in purple, profiles from *rtc3* G100STOP (C-terminal tail truncation) strains shown in blue. (c) Serial dilution spot assays of *S. pombe* wildtype and *rtc3* mutant cultures grown to saturation in EMM with 0.5% glucose at 32 °C and then continuously incubated in the same media. 10-fold serial dilutions of the normalized samples at OD_600_ of 0.25 were spotted on EMM plates containing 2% glucose and incubated at 32 °C. Day 1 corresponds to the 24 h time point of continuous incubation. (d) Live-cell differential interference contrast images of wildtype and *rtc3∆* cells grown continuously in EMM containing 0.5% glucose for 0, 7 and 10 days. Scale bar represents 5 μm. Representative images from 4 independent experiments are shown.

To examine the role of SNOR in translation recovery, we generated *rtc3*Δ (SNOR-knockout) and SNOR mutant strains carrying ribosome-binding-deficient or C-terminal tail deletions, all introduced by knock-in at the endogenous *rtc3* locus under native regulatory control. We first compared growth of SNOR-knockout cells to wild-type cells under different glucose conditions (2%, 0.5% and 0.02%) and observed no major growth defects under any condition (Fig. 5a and Extended Data Fig. 9a). We next incubated cells in low glucose (0.5%) for 7 days and assessed protein synthesis by polysome profiling. By day 7, polysomes shifted into 80S monosomes in all strains, consistent with global translation shutdown (Extended Data Fig. 9b). After 2 h of glucose reintroduction, we observed restart in protein synthesis as demonstrated by the presence of polysomes in wild-type cells and a decrease in 80S monosomes (Fig. 5c,d and Supplementary Figs. 6 and 7). By contrast, SNOR-knockout cells did not recover polysomes. Similarly, both SNOR knock-in mutants, including the ribosome-binding-deficient mutant and the C-terminal tail deletion mutant, did not recover polysomes. These results demonstrate that SNOR association with the ribosome and its C-terminal tail are both required for translation restart.

To assess the physiological consequences of impaired ribosome reactivation, we monitored growth recovery and viability following prolonged glucose limitation. Wild-type, SNOR-knockout and SNOR mutant strains were maintained in 0.5% glucose for up to 7 days and reintroduced to EMM plates containing 2% glucose medium to assess viability by spot assay. All strains exhibited comparable viability at day 1 and day 2 of glucose depletion (Fig. 5e, Extended Data Fig. 9c and Supplementary Fig. 8). However, starting at day 4, SNOR-knockout and both ribosome-binding-deficient and C-terminal tail-deleted knock-in strains displayed a progressive loss of viability relative to wild type, coinciding with increased SNOR protein levels in wild-type cells (Fig. 2c) and the onset of translational shutdown^24^. By day 7, all mutant strains exhibited pronounced viability defects (Fig. 5e) and a granulated, vacuolated morphology (Extended Data Fig. 9d).

Our high-resolution in situ cryo-ET structure resolves hypusination of eIF5A at K52 (Fig. 5b). The hypusine moiety forms hydrogen-bond interactions with N106 in the C-terminal region of SNOR (Fig. 5b), suggesting that eIF5A participates with SNOR in ribosome reactivation.

To test this, we inhibited eIF5A hypusination using the spermidine analogue N¹-guanyl-1,7-diaminoheptane (GC7)^38,39^ and then examined protein synthesis restart following glucose depletion. GC7 treatment significantly impaired recovery in wild-type cells, resulting in an approximately 50% reduction in the polysome-to-monosome ratio compared with untreated controls, consistent with a defect in ribosome reactivation (Fig. 5f,g). Similarly, SNOR-knockout cells treated with GC7 were unable to restart translation. We further assessed the physiological consequences of impaired ribosome reactivation by measuring cell viability following glucose reintroduction. SNOR-knockout cells treated with GC7 exhibited reduced viability already at day 1 of glucose depletion and a markedly more severe growth defect by day 7, consistent with an additive effect (Fig. 5h).

Together, these results provide direct mechanistic evidence that SNOR cooperates with hypusinated eIF5A, whose modified lysine residue engages the SNOR C-terminal tail to promote efficient ribosome reactivation upon glucose reintroduction.

## Discussion

Cells confronted with environmental stress often enter dormancy as a survival strategy. During this state, energy-intensive processes such as protein synthesis are downregulated to preserve resources until favourable conditions return. Here, by combining in situ cryo-ET, single-particle cryo-EM and functional analyses, we identify SNOR as a stress-induced factor that associates with dormant ribosomes and promotes efficient translational restart upon recovery from glucose-dependent dormancy.

**Fig. 6:**
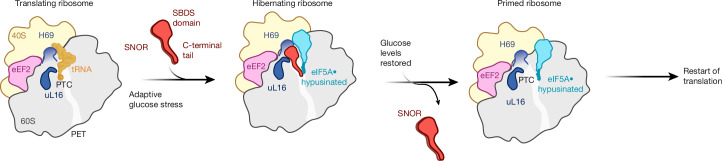
SNOR–eIF5A cooperation licenses ribosome reactivation following glucose-induced dormancy. Schematic model illustrating the role of SNOR in ribosome surveillance and translational restart during glucose-dependent cellular dormancy. Left, during active translation, ribosomes engage tRNAs at the PTC and PET. Under adaptive glucose stress, SNOR is induced and binds dormant ribosomes via its SBDS domain, inserting its C-terminal tail into the PET. SNOR forms a tripartite interface with hypusinated eIF5A and ribosomal protein uL1, stabilizing helix H69 and reorganizing the ribosomal active site into a hibernation-compatible but restart-competent state. Upon glucose restoration, SNOR dissociates, and eIF5A remains poised on the ribosome, priming ribosomes for efficient reactivation and restart of protein synthesis. Created in BioRender. Gluc, M. https://BioRender.com/schah0m (2026).

Following glucose depletion, dormant ribosomes associate with SNOR together with Oga1 (Stm1 in *S. cerevisiae*), eIF5A, and eEF2 (Fig. 6 and Supplementary Video 1). Phylogenetic analysis shows that SNOR is highly conserved across fungi and selectively induced by altered glucose availability, but not by other nutrient or osmotic stresses. Notably, SNOR is largely absent from Microsporidia. Microsporidia have particularly small genomes, as they have undergone significant genome reduction to become obligate intracellular parasites^34^. The microsporidia-specific hibernation factor MDF1^40^, although structurally distinct from SNOR, occupies a similar ribosomal position and may therefore serve an analogous role.

SNOR shares homology with Sdo1 (SBDS in humans), a ribosome biogenesis factor during late stages of assembly^36,41^. Rather than participating in ribosome biogenesis, SNOR binds the PTC of dormant 80S ribosomes and inserts its C-terminal tail into the PET. The positioning of the SNOR C-terminal tail within the exit tunnel raises the possibility that SNOR senses nascent polypeptides unlinked from tRNA but retained in hibernating ribosomes prior to translation restart. This configuration also stabilizes helix H69 in an outward orientation and prevents adoption of the inactive conformation that was previously observed under similar stress conditions^24^. By maintaining the structural integrity of the PTC region, SNOR preserves ribosomes in a restart-competent configuration.

Unlike canonical hibernation factors that primarily block translation, SNOR forms a tripartite interface with hypusinated eIF5A and ribosomal protein uL1. This interaction stabilizes the L1 stalk and reorganizes the active site into a hibernation-compatible yet restart-competent state. eIF5A is a conserved translation factor that carries a hypusine modification and is best known for its role in promoting peptide-bond formation during translation elongation, particularly at stalling sequences such as consecutive proline residues, as well as in termination and quality control processes^35,42,43^. Our high-resolution in situ cryo-ET maps resolve direct interactions between the hypusinated lysine of eIF5A and the C-terminal tail of SNOR, revealing the molecular basis for their cooperative stabilization on dormant ribosomes. Of note, the SNOR–eIF5A interface retains eIF5A in a poised configuration that licenses ribosome reactivation once glucose levels are restored (Fig. 6).

Recent work identified Dap1b in vertebrate oocytes as a hibernation factor that also interacts with eIF5A at the exit tunnel^22^ (Supplementary Fig. 9). However, Dap1b lacks a structured SBDS-like domain. Our findings raise the possibility that higher eukaryotes may utilize additional factors analogous to SNOR to couple exit tunnel surveillance with restart-licensing mechanisms during recovery from stress.

Together, our study defines SNOR as a conserved carbon stress-induced regulator that engages the eukaryotic ribosome active site to prime re-entry into translation. More broadly, these findings provide a mechanistic framework for how ribosomes transition from dormancy to active translation and highlight the power of integrative in situ structural biology to uncover dynamic regulatory layers of translational control.

## Methods

### Sample preparation for cryo-ET

Cryo-ET samples were prepared using 200-mesh R2/2 copper grids (Quantifoil) plasma-cleaned for 30 s in 75% argon/25% oxygen using a 1070 plasma cleaner (Fischione). *S. pombe* cells were grown for 7 days in EMM containing 0.5% (w/v) glucose and diluted in glucose-free EMM to OD_600_ = 0.6 immediately before freezing. A 4 µl aliquot was applied to the carbon side of the grid and back-side blotted for 1 s (Whatman 597 paper; paper contact sensing mode with 1.5 mm movement) using a Leica EM GP2 plunger operated at 23 °C and 100% humidity. Grids were vitrified in liquid ethane.

### Cryo-FIB milling

Vitrified grids were mounted in Cryo-FIB Autogrids (ThermoScientific, 1205101) under liquid nitrogen and transferred to an Aquilos 2 cryo-FIB/SEM (ThermoScientific). The stage and anti-contamination shield were maintained at −194 °C. After scanning electron microscope screening (5 kV, 13 pA), grids were coated with trimethyl(methylcyclopentadienyl)platinum(IV) (GIS, 120 s), preceded and followed by 15 s sputter coating (30 mA). Positions were set using MAPS, and lamellae were milled using AutoTEM (30 kV; 1 nA to 30 pA) and thinned manually to ~160 nm at 30 pA. During final thinning, the stage was tilted by +0.5° to improve surface polishing and reduce curtaining. A final sputter coating (3 s, 10 mA) reduced charging during transmission electron microscopy acquisition and facilitated tilt-series alignment.

### Mitochondria-focused cryo-ET acquisition

#### Data collection

To initially assess ribosomes proximal to mitochondria, lamellae were screened and tilt series were targeted to mitochondrial regions. Data were collected on a Titan Krios G4i (ThermoScientific) operated at 300 kV, equipped with an energy filter and Falcon4i detector in zero-loss mode. Lamellae were mapped at 14.39 Å per pixel (−70 µm defocus; 70 µm objective aperture; 20 eV slit). Mitochondria were selected in SerialEM^44^, guided by SPACEtomo segmentation and PACEtomo^45^ for beam image-shift parallel acquisition. Tilt series were acquired at 64,000× (1.933 Å per pixel), using a 50 µm C2 and 70 µm objective aperture (10 eV slit), in nanoprobe mode (spot size 5; 1.4 µm illumination). A dose-symmetric scheme (3° increments, −65° to +49°, centred on ~−8° pre-tilt) was used. Movies were recorded in counting mode (880 ms; ~3.34 e^−^ Å^−2^ per tilt; total dose ~130 e^−^ Å^−^^2^). Target defocus ranged from −2 to −6 µm. Seventy-eight tilt series were selected for subtomogram averaging based on dose, thickness, contamination and cellular integrity.

#### Data processing

Stage tilts in .mdoc files were corrected for lamella pre-tilt. EER frames were converted to TIFF (RELION v4.0.1^46^), motion-corrected, and contrast transfer function (CTF)-estimated (−2 to −10 µm) in Warp 1.09^47^. After manual inspection, tilt series were aligned in AreTomo2^48^, defocus handedness verified, and 3D-CTF–corrected tomograms reconstructed at 15.44 Å per pixel in Warp.

#### Ribosome picking and classification

Ribosomes were identified using PyTom(1.1)^49^ template matching with a 40 Å low-pass–filtered *S. pombe* OMM-associated ribosome map (EMD-50266). To distinguish OMM-tethered from cytosolic ribosomes, mitochondrial membrane masks were generated. Two tomograms were segmented in Dragonfly (Comet Technologies) to train a 2.5D U-Net (five-slice input; depth 5; patch size 64 pixels), which was applied batch-wise to generate dilation masks around mitochondria. Masked particles were extracted and curated in ArtiaX^50^.

#### Subtomogram averaging

Particles were extracted in Warp and refined in RELION. After iterative 3D classification and refinement, particles were optimized in M (part of the Warp–M suite developed for high-resolution in situ cryo-EM and cryo-ET data refinement)^47^ for pose, deformation, and CTF parameters. cryoDRGN-ET^51^ enabled unsupervised classification, followed by further refinement in M. The final OMM-associated ribosome reached 6.0 Å resolution (Fourier shell correlation (FSC) = 0.143). Cytosolic ribosomes, curated separately, were refined to 6.6 Å resolution (Extended Data Fig. 1 and Supplementary Fig. 2).

### Whole-cell high-resolution in situ cryo-ET

#### Data collection

To obtain a comprehensive and higher-resolution view, we next collected a large dataset sampling ribosomes throughout the cell. Lamellae (<20 µm width) were prepared as above to minimize beam-image shift. Tilt positions were distributed across the entire cell and grouped within <15 µm focus distance to reduce beam-shift aberrations. Data were collected at 1.526 Å per pixel (Extended Data Fig. 1). In total, 1,012 tilt series were acquired across three dose groups (group 1: 518; 3.33 e^−^ Å^−2^; group 2: 375; 3.49 e^−^ Å^−2^; group 3: 85; 3.59 e− Å^−2^).

#### Preprocessing and reconstruction

Preprocessing (EER to tomogram) was automated in AreTomo3^48^. Bad tilts were excluded (DarkTools threshold = 0.8), and alignment, thickness estimation, and pre-tilt correction were automated. Only tomograms with 60–225 nm thickness and <100 nm global shifts were retained. EER fractions were converted to TIFF and upsampled (0.763 Å per pixel) for motion correction and per-tilt CTF estimation in Warp (v2.0.0dev36). Tomograms were reconstructed with 3D CTF correction at 12.208 Å per pixel.

#### Subtomogram averaging and refinement

Seventy-four group 3 tilt series were first processed. Ribosomes were identified using PyTom (v0.7.2) using the previous 5.5 Å consensus hibernation map (Extended Data Fig. 1a; 30 Å low-pass–filtered) as template, yielding 9,071 candidates. After RELION (v4.0.1) refinement/classification, 8,829 particles were refined in M (v2.0.0dev36) with iterative optimization of ImageWarp, ParticlePose, per-particle defocus, VolumeWarp, stage-angle, and deformation grids. This subset reached 4.73 Å resolution and was used as an improved template for template matching in the remaining 715 tomograms, yielding 95,485 particles. After RELION cleanup, 88,206 particles were refined in Warp/M at 1.526 Å per pixel. Tilt-series and per-tilt contributions were weighted during refinement. Third-order Zernike polynomials modelled higher-order aberrations once sub-4 Å resolution was reached. Final refinement after upsampling to 1.0 Å per pixel yielded a 3.38 Å reconstruction (FSC = 0.143). Local resolution analysis showed improved resolution in the ribosomal core and SNOR/eIF5A region which is resolved between 2.8 and 3.3 Å resolution (Extended Data Figs. 1 and 2).

### Gene detection and phylogenetics

Annotated protein sequences for all fungal (2,248 genomes) and mammalian (263 genomes) reference genomes (available on GitHub at https://github.com/cassprince/SNOR_conservation) were downloaded from the NCBI RefSeq database on 28 December 2024 and 3 April 2025, respectively. This resulted in a subset of RefSeq that includes approximately one genome per species^52,53^. SNOR protein sequences were detected using HMMER v3.3 (hmmsearch) (hmmer.org; Supplementary Table 3) with an E-value cutoff of 0.05. To search for SNOR, HMMER profiles were built with six protein sequences annotated as Rtc3 from Ascomycota genomes by the NCBI Eukaryotic Genome Annotation Pipeline^53^. Query sequences are given in Supplementary Table 3. Only protein hits fewer than 200 amino acids in length were considered SNOR candidates to minimize the likelihood of detecting larger aberrant SBDS proteins.

18S rRNA sequences from the fungal genomes described above were analysed using a previously described workflow^54^. In brief, the sequences were identified using BLAST v2.16.0, aligned using MAFFT v7.520, and applied to FastTree v2.1.10 to produce a maximum-likelihood tree. The constructed tree was midpoint rooted with the phangorn v2.12.1 package and visualized with ggtree v3.12.0. Taxonomic classifications were assigned according to the NCBI Taxonomy database. The complete list of genome accession numbers, taxonomic classification, and SNOR presence for all surveyed genomes can be found on Figshare (10.6084/m9.figshare.31350286 (ref. ^33^)).

### RT–qPCR

*S. pombe* cells were grown overnight at 30 °C with agitation in YES medium, then transferred to EMM supplemented with 0.5% (w/v) glucose and cultured for an additional 3 days. Culture samples were collected daily and flash frozen in liquid nitrogen for subsequent analysis. RNA was extracted from 10^7^ cells per sample, using the RNeasy Kit (QIAGEN, 74104) following the manufacturer’s protocol. Quantity and quality of RNA samples were assessed using a NanoDrop (Thermo Scientific). cDNA was synthesized using the Verso cDNA Synthesis Kit (Thermo Scientific, AB1453A). Real-time quantitative PCR was performed on the StepOne Plus system (Applied Biosystems) using SYBR Green Master mix (Thermo Scientific, A25742). Gene expression levels were quantified using the ΔΔCt method and normalized to the expression of the housekeeping gene *act1* (actin). Primer sequences are listed in Supplementary Table 4.

### Monitoring SNOR protein levels using different stress conditions

*S. pombe* cells were grown overnight at 30 °C with agitation in EMM. The overnight cultures were washed 3 times with PBS (Phosphate Buffered Saline) and used to inoculate fresh EMM supplemented with either 0, 0.5, 2 or 20% glucose or EMM (2% glucose) lacking either amino acids or nitrogen or EMM (2% glucose) supplemented with high levels of salt (1.1 M NaCl). Cells were inoculated to a starting OD_600_ of 0.1 and cultured at 30 °C with constant agitation for 40 min after which samples were collected for cell lysis. For cell lysis 1.5 OD_600_ units of cells were resuspended in 500 μl water. Fifty microlitres of 1.85 M NaOH was added and incubated on ice for 10 min. Trichloroacetic acid (TCA) was then added to a final concentration of 10%, followed by an additional 10 min incubation on ice. Samples were centrifuged at 14,000*g* for 15 min at 4 °C. Pellets were then resuspended in 1× SDS–PAGE sample buffer (50 mM Tris-HCl pH 6.8, 2% SDS, 1% β-mercaptoethanol, 6% glycerol, 0.004% bromophenol blue) for western blot analysis. Proteins were resolved using SurePAGE gels (GenScript, M00653) with MES running buffer (GenScript, M00677) and transferred onto 0.2 μm nitrocellulose membranes (LI-COR, 926-31092). Membranes were blocked for 1 h at room temperature in 5% milk prepared in 1× PBST (0.1% Tween-20). After blocking, antibodies diluted in 2% milk in 1× PBST were incubated with membranes. Primary antibodies used: Flag (Genscript, A00187) and GAPDH (Proteintech, 60004-1-Ig). Secondary antibodies used: anti-mouse (Invitrogen, A21058). The LI-COR Odyssey imager was used for detection.

### Ribosome purification

*S. pombe* cells were cultured overnight at 30 °C with agitation in 500 ml of YES medium. Cells were collected by centrifugation at 3,000*g* for 5 min at room temperature, washed with lysis buffer (20 mM HEPES pH 7.4, 100 mM KCl, 5 mM MgCl_2_, 1% (v/v) Triton X-100), and centrifuged again at 5,000*g* for 5 min. The resulting pellet was resuspended in lysis buffer supplemented with 0.04 U µl^−1^ RNase inhibitor (RiboLock, Thermo Fisher, EO0382) and flash frozen in liquid nitrogen as small drops. Frozen pellets were ground to a fine powder in a mortar and pestle under liquid nitrogen. The powder was resuspended 1:1 (v/w) in lysis buffer and the lysate was clarified by centrifugation at 5,000*g* for 5 min at 4 °C to remove cell debris, followed by a second centrifugation at 14,000*g* for 10 min at 4 °C. The resulting supernatant was layered over a 50% sucrose cushion (50% w/v sucrose, 20 mM HEPES pH 7.4, 100 mM KCl, 5 mM MgCl_2_) and centrifuged at 43,000 rpm (50.2 Ti rotor, ~225,000*g*) for 20 h at 4 °C. Following centrifugation, the ribosome pellet was resuspended in either resuspension buffer (20 mM HEPES pH 7.4, 60 mM KCl, 5 mM MgCl_2_) or ribosome splitting buffer (20 mM HEPES pH 7.4, 1 M KCl, 5 mM MgCl_2_, 1 mM puromycin). For subunit separation, ribosome samples were incubated in ribosome splitting buffer for 1 h at 4 °C, then loaded onto a continuous 10–40% sucrose gradient (10–40% w/v sucrose, 20 mM HEPES pH 7.4, 500 mM KCl, 5 mM MgCl_2_, 1 mM DTT) and centrifuged at 21,000 rpm for 20 h at 4 °C. Gradients were analysed using a BIOCOMP Piston Gradient Fractionator system, and fractions corresponding to the small and large ribosomal subunits were collected. Subunit samples were concentrated using Amicon Ultra Centrifugal Filter Concentrator tubes (100 kDa MWCO, Millipore, ref. UFC8100). Three biological replicates of crude ribosomes were purified from cells grown under normal conditions and from cells subjected to 7 days of glucose depletion and were analysed by mass spectrometry.

### LC–MS/MS analysis of ribosomes

Crude *S. pombe* ribosome samples purified from normal conditions (day 1) and glucose-depleted conditions (day 7) were processed by Taplin Biological Mass Spectrometry Facility. 10 µl (20 ng µl^−1^) of modified sequencing-grade trypsin (Promega) was spiked into ribosome sample in resuspension buffer (20 mM HEPES, 5 mM MgCl_2_, 100 mM KCl) and the samples were placed in a 37 °C room overnight. Samples were acidified by spiking in 10 µl 20% formic acid solution and desalted by STAGE tip^55^. On the day of analysis the samples were reconstituted in 5–10 µl of HPLC solvent A (2.5% acetonitrile, 0.1% formic acid). A nano-scale reverse-phase HPLC capillary column was created by packing 2.6 µm C18 spherical silica beads into a fused silica capillary (100 µm inner diameter × ~30 cm length) with a flame-drawn tip^56^. After equilibrating the column each sample was loaded via a Thermo EASY-LC (Thermo Fisher Scientific). A gradient was formed and peptides were eluted with increasing concentrations of solvent B (90% acetonitrile, 0.1% formic acid). As peptides eluted they were subjected to electrospray ionization and entered into an Orbitrap Exploris480 mass spectrometer (Thermo Fisher Scientific). Peptides were detected, isolated, and fragmented to produce a tandem mass spectrum of specific fragment ions for each peptide. Peptide sequences (and hence protein identity) were determined by matching protein databases with the acquired fragmentation pattern by the software program, Sequest^57^ (Thermo Fisher Scientific). All databases include a reversed version of all the sequences, and the data was filtered to between a one and two percent peptide false discovery rate. The complete list of identified peptides can be found on Figshare (10.6084/m9.figshare.31350286 (ref. ^33^)).

### Recombinant SNOR, SNOR mutants and eIF5A purification

A pET28a vector encoding N-terminal 6× His-tagged SNOR or eIF5A was expressed in *E. coli* BL21-CodonPlus (DE3) competent cells (Agilent, 230245). Site-directed point mutations in SNOR were generated using the QuickChange mutagenesis kit (Agilent, 210518) and validated by whole plasmid sequencing by Plasmidsaurus. Cells were cultured in LB medium (Fisher Bioreagents, BP1426) at 37 °C until reaching an OD_600_ = ~0.6. Protein expression was induced with 1 mM IPTG, followed by overnight incubation at 18 °C. Cells were collected by centrifugation, and the resulting pellet was resuspended in lysis buffer (50 mM HEPES pH 7.5, 1 M KCl, 10% glycerol, 6 mM β-mercaptoethanol, 0.5× protease inhibitor cocktail) and lysed using a French Press. The lysate was clarified by two rounds of centrifugation at 20,000 rpm for 30 min at 4 °C using a 50.2 Ti rotor. The cleared supernatant was loaded onto a 5 ml HisTrap HP column (Cytiva, 17524802) using a P1 pump at 4 °C and washed with 1–2 column volumes (CV) of wash buffer (50 mM HEPES-KOH pH 7.5, 500 mM KCl, 15 mM imidazole, 10% glycerol, 6 mM β-mercaptoethanol). The column was then transferred to an ÄKTA Pure fast protein liquid chromatography system (Cytiva) and washed with an additional 5 CV of wash buffer. Bound proteins were eluted using a step gradient with 2–3 CV of 15%, 30% and 80% Elution Buffer (50 mM HEPES-KOH pH 7.5, 500 mM KCl, 300 mM imidazole, 10% glycerol, 6 mM β-mercaptoethanol). Fractions corresponding to the 30% and 80% elution peaks were pooled and transferred to 7 kDa MWCO dialysis tubing (ThermoFisher, 27968700), then dialysed overnight at 4 °C in dialysis buffer (50 mM HEPES-KOH pH 7.5, 150 mM KCl, 30 mM imidazole, 10% glycerol). The protein samples were subsequently concentrated using Amicon Ultra Centrifugal Filter Concentrator tubes (3 kDa MWCO, Millipore, UFC8003), aliquoted, flash frozen in liquid nitrogen, and stored at –80 °C.

### Ribosome co-pelleting assay

The 40S, 60S and 80S ribosome samples were thawed on ice and diluted to 1 µM using Binding buffer (50 mM HEPES pH 7.7, 150 mM KCl, 15 mM MgCl_2_, 0.02% C12E8, 5% v/v glycerol). Purified wild-type SNOR and SNOR point mutants, as well as purified wild-type eIF5A were thawed on ice and diluted to 75 µM using the Binding buffer. To prepare the reactions, the purified ribosome samples were added to a final concentration of 0.2 µM and the purified protein samples were added to a final concentration of 2 µM. The reactions were incubated for 30 min at 30 °C and layered (1:1) over a 40% sucrose cushion (40% w/v sucrose, 20 mM HEPES pH 7.4, 100 mM KCl, 5 mM MgCl_2_). Samples were centrifuged at 100,000 rpm for 2 h at 4 °C. Following the spin, the supernatant was discarded and the resulting pellet was resuspended in 1× SDS–PAGE sample buffer (50 mM Tris-HCl pH 6.8, 2% SDS, 1% β-mercaptoethanol, 6% glycerol, 0.004% bromophenol blue) for analysis by western blot. Samples were resolved on a SurePAGE gel (Genscript, M00653) using MES buffer (Genscript, M00677) and transferred onto a 0.2 μm nitrocellulose membrane (LI-COR, 926-31092). Membrane was blocked in 5% milk in 1× PBST (0.1% Tween-20) for 1 h at room temperature. After blocking, antibodies diluted in 2% milk in 1× PBST were incubated with membranes. Primary antibodies used: 6× His (Genscript, A00186), RPS6 (Cell Signaling, 2217), RPL4 (Invitrogen, cat. no MA5-56865). Secondary antibodies used: anti-mouse (Invitrogen, A21058), anti-rabbit (Invitrogen, A32735). The LI-COR Odyssey imager was used for detection.

### Cryo-EM sample preparation and data collection

The large ribosomal subunit and recombinant SNOR protein were purified as described above. Freshly prepared 60S ribosome samples were diluted to 270 ng µl^−1^ in resuspension buffer A (20 mM HEPES-KOH pH 7.4, 60 mM KCl, 5 mM MgCl_2_). To reconstitute the 60S–SNOR complex, purified SNOR protein was initially diluted using buffer A and added to the reaction to a final concentration of 1 µM. The reaction was incubated for 30 min at 30 °C and then moved to ice. Quantifoil Cu200 R2/2 grids were coated with a 3 nm carbon layer using a Safematic carbon coater (Rave Scientific) prior to sample application. Grids were plasma-cleaned for 15 s at 15 mA using a Pelco easiGlow system to render the surface hydrophilic. Five microlitres of the reaction mixture were then applied to the grids, followed by a 60 s incubation. Grids were blotted for 9 seconds at blot force +7 and plunge-frozen in liquid ethane using a Vitrobot Mark IV (ThermoScientific) operated at 4 °C and 100% humidity. Cryo-EM data acquisition was carried out using a Titan Krios transmission electron microscope (ThermoScientific) operated at 300 kV. The microscope was equipped with a K3 direct electron detector and a Gatan Quantum energy filter set to a 10 eV slit width. Movies were recorded in counting mode at a nominal magnification of 105,000×, yielding a calibrated pixel size of 0.84 Å. A total of 7,277 movies were acquired, each consisting of 40 frames and accumulating a total electron dose of 50 e– Å^−2^. The target defocus range was set between −1.6 and −0.6 μm.

### Cryo-EM data processing

Cryo-EM movies were motion-corrected and dose-weighted using cryoSPARC^58^, and CTF parameters were estimated for each micrograph in RELION^59^. An initial set of 821,035 particles was picked using a Laplacian blob-based picker in RELION. Ab initio reconstruction and an initial round of 3D classification were then performed to identify ribosomal particles. After the first round of 3D classification, 324,887 particles containing SNOR were retained, while particles that showed low-resolution features or did not yield a meaningful 3D 60S class were excluded. A second round of 3D classification was performed to improve particle homogeneity, followed by focused 3D classification with signal subtraction using a soft mask encompassing the SNOR region to enhance local resolution. Selected particles were subjected to 3D auto-refinement, per-particle CTF refinement, and beam-tilt correction in RELION. The final reconstruction was obtained from 110,548 particles and sharpened. Global resolution was estimated using gold-standard FSC between independently refined half-maps, applying the FSC = 0.143 criterion.

### Model building

#### Large ribosomal subunit and SNOR complex

Following data processing, a previously generated model of the *S. pombe* large ribosomal subunit (PDB: 9AXU) and AlphaFold prediction model of SNOR (AF-Q9P7K6-F1-v4) were docked into the cryo-EM map using ChimeraX. SNOR was further rigid body fit into the observed density using COOT and manually adjusted based on the observed density. PHENIX was used to refine the model in the 60S–SNOR map with five macrocycles of real-space refinements applying Ramachandran, side-chain rotamer, protein secondary structure and nucleotide restraints to correct for clashes. The final model was validated using MolProbity in PHENIX. All figure generation was done using ChimeraX.

#### In situ cryo-ET hibernating 80S ribosome

The previously determined *S. pombe* ribosome model (PDB: 9AXV) was docked into the in situ cryo-ET map using ChimeraX. Homology models of eEF2 and Stm1 were generated by using PHYRE2^60^ with one-to-one threading and aligning *S. pombe* sequences to the AlphaFold predicted structures of their respective *S. cerevisiae* homologues. The generated models were docked into the cryo-ET map using ChimeraX and further adjusted as rigid bodies in COOT based on the local density features and visible side chains. AlphaFold models of SNOR and eIF5A were initially fitted as rigid bodies into the cryo-ET density and manually adjusted based on the resolved side chains of the amino acids and the high-quality local EM density in this region. The L1 stalk and uL1 protein were manually adjusted in COOT based on the corresponding EM density. The complete atomic model of the hibernating *S. pombe* 80S ribosome was refined against the in situ cryo-ET map using five macrocycles of real-space refinement in PHENIX, applying Ramachandran, side-chain rotamer, protein secondary structure, and nucleotide restraints. The final model was validated using MolProbity as implemented in PHENIX.

### Small-scale in vitro translation

Purified mRNA of N-terminal 3× Flag-tagged reporter and residues 1-38 of filamin C connected via a linker was translated in RRL (Promega, L4540). The lysate was diluted to 66.7% (v/v) with a translation mix containing purified RNA (final concentration 0.5 µg µl^−1^), 3 µM SNOR, 3 µM eIF5A, a combination of both, or 3 µM BSA as a control. The final reaction buffer also included 0.04 U µl^−1^ RNase inhibitor (Promega), 0.5× protease inhibitor cocktail (Promega), 81 mM KCl, 2 mM magnesium acetate, and 24 µM amino acid mix. Reactions were incubated at 32 °C for 25 min, then placed on ice. Following the incubation, samples were analysed by SDS–PAGE and western blotting to detect levels of Flag-tagged translation product.

### In vivo functional experiments

#### SNOR-knockout and mutant strain generation

To construct the *rtc3∆::ura4*^*+*^ strain, 300 bp of the *rtc3*^*+*^ 5′ untranslated region (UTR) and 3′ UTR were amplified from the genomic DNA of wild-type cells. The resulting amplicons were cloned into the BamHI/PstI site and KpnI/XhoI site, respectively, of a pSK plasmid containing the *ura4*^*+*^ gene within the PstI/KpnI sites using the Gibson assembly method. A PCR product containing the UTRs and *ura4*^*+*^ was then transformed into *ura4-D18* cells using a lithium acetate method^61^. Transformants were selected on EMM agar plates lacking uracil and the correct deletion was verified by whole-cell PCR using oligonucleotides flanking the *rtc3* UTR sequences and internal to *ura4*^*+*^.

To create *rtc3* mutant strains, the open reading frame of *rtc3*^*+*^ with 300 bp each of 5′ and 3′ flanking sequences was amplified from wild-type genomic DNA and Gibson cloned into the PstI site of pIRT2. Point mutations in *rtc3* were then generated by site-directed mutagenesis and confirmed by DNA sequencing. The resultant pIRT2 constructs were each transformed into the *rtc3∆::ura4*^*+*^ strain, and transformants were isolated on EMM agar plates lacking uracil and leucine. Mutant *rtc3* strains were then selected by growing transformants overnight under non-selective conditions and plating on EMM plates containing uracil, leucine, and 5-fluoroorotic acid (1.5 mg ml^−1^). Replacement of *ura4*^*+*^ with each correct *rtc3* mutant was confirmed by whole-cell PCR and DNA sequencing. PCR products and plasmids were sequenced by Plasmidsaurus using Oxford Nanopore Technology with custom analysis and annotation.

Differential interference contrast images were acquired using a Zeiss Axio Observer inverted epifluorescence microscope with Zeiss 63× oil (1.46 NA) objective and captured using Zeiss ZEN 3.0 (Blue edition) software. A singular medial Z slice was obtained. All images were further processed using ImageJ. A list of *S. pombe* strains used in this study is provided in Supplementary Table 5.

#### Genomic Flag tagging of SNOR

To add two Flag tags to the N-terminus of SNOR, a CRISPR–Cas9-based approach was used. A guide RNA (gRNA) targeting a region near the SNOR start codon was selected using https://crispr.dbcls.jp/ and cloned into a Cas9 gRNA plasmid to induce a double-stranded break at the desired genomic location. The plasmid backbone was a gift from H. Levin. Cloning was performed using Q5 polymerase, following the manufacturer’s instructions. In parallel, a double-stranded repair template was synthesized as a gBlock, containing in order: 200 nucleotides of the SNOR 5′, two Flag tags beginning with AUG, and 200 nucleotides of the SNOR coding sequence (CDS). *S. pombe* cells (*S. pombe* YHL 912 h-, ura4-294 leu1-32 gift from H. Levin) were then co-transformed with the gRNA-Cas9 plasmid and the repair template. The transformation protocol was adapted from Levin et al.^62^. After transformation, cells were plated on EMM lacking leucine and incubated for 3 days. Next, individual colonies were transferred to YES medium to allow for the loss of the gRNA-Cas9 plasmid. Colonies were checked for Flag tag incorporation by colony PCR as well as western blot. A list of *S. pombe* strains used in this study is provided in Supplementary Table 5.

#### Glucose restart experiment and polysome gradient profiling

Wild-type, *rtc3∆*, and *rtc3* mutant cells were grown to mid-log phase at 32 °C in EMM containing 2% glucose overnight, adjusted to the same OD_600_ of 0.05 in 0.5% glucose EMM, (considered day 0 of the experiment) and incubated continuously for the remainder of the experiment at 32 °C. Each day, an aliquot was removed from each culture and adjusted to an OD_600_ of 0.25. Tenfold serial dilutions of the normalized samples were spotted on EMM plates containing 0.5% glucose and incubated at 32 °C. A technical replicate was performed at each time point. All samples were spotted on the same plate, and plates were imaged 3 days later. The experiment was performed twice.

For analysing polysome gradients, cells were first incubated in EMM supplemented with 0.5% (w/v) glucose for 7 days. Following glucose depletion, cells were collected, washed with double-distilled H_2_O, resuspended in fresh YES (Yeast Extract with Supplements) medium to an OD_600_ of 1 and incubated for 2 h at 30 °C with agitation. Following the incubation, cells were treated with 100 μg ml^−1^ cycloheximide for 15 min, then collected and lysed as described above (see ‘Ribosome purification’). The clarified lysate was quantified by measuring absorbance at 260 nm, and 10 A260 units were loaded onto a continuous 10–50% sucrose gradient. Gradients were centrifuged at 230,000*g* for 2.5 h at 4 °C. Polysome gradient profiles were analysed using a BIOCOMP Piston Gradient Fractionator and visualized with GraphPad Prism 10.

#### Inhibition of eIF5A hypusination by GC7 treatment

Wild-type and *rtc3Δ::ura4*^*+*^ cells were grown overnight to mid-log phase at 30 °C in EMM containing 2% glucose. Cultures were then adjusted to an OD_600_ of 0.05 in fresh EMM supplemented with 0.5% (w/v) glucose, with or without 20 µM GC7 sulfate (deoxyhypusine synthase inhibitor; MedChemExpress, HY-108314A). This time point was designated day 0 of the experiment, and cultures were maintained at 30 °C for 7 days. To maintain effective inhibition of hypusination, GC7 sulfate was additionally spiked into treated cultures after 3 and 6 days of incubation.

To assess the effect of eIF5A hypusination inhibition on the ability of cells to resume growth, aliquots were collected from each culture on days 1 and 7 and adjusted to an OD_600_ of 0.25. Cells were subjected to a serial dilution spotting assay as described above. Untreated cells were plated on EMM plates containing 2% glucose, whereas GC7-treated cells were plated on EMM plates containing 2% glucose supplemented with 20 µM GC7 sulfate. Plates were incubated for 3 days at 32 °C prior to imaging.

For polysome gradient analysis, cells were collected after 7 days of incubation in EMM supplemented with 0.5% (w/v) glucose, with or without GC7 treatment as described above. Cells were washed with double-distilled water and resuspended to an OD_600_ of 1.0 in fresh YES medium, with or without the addition of 20 µM GC7 sulfate. Cultures were incubated for 2 h at 30 °C with agitation, after which polysome profiles were analysed as described above.

### 3D rendering of tomograms and EM volumes

Models of mitochondrial membranes displayed in Fig. 1 and in the video (Supplementary Video 1) were segmented in Dragonfly and then imported into Blender 4.2 (https://www.blender.org/) using Microscopy Nodes plugin^63^, together with the corresponding tomogram. Ribosomes were placed in the 3D rendering layout following their positions in the.star files using the plugin Molecular Nodes (v4.4.3). All other electron microscopy volumes were prepared with ChimeraX.

### Reporting summary

Further information on research design is available in the Nature Portfolio Reporting Summary linked to this article.

## Online content

Any methods, additional references, Nature Portfolio reporting summaries, source data, extended data, supplementary information, acknowledgements, peer review information; details of author contributions and competing interests; and statements of data and code availability are available at 10.1038/s41586-026-10530-7.

## Supplementary information

Supplementary Information Supplementary Figs. 1–9 include uncropped immunoblot images, supportive SNOR expression and co-pelleting assays, polysome profiles, viability assays, and structural comparisons. Supplementary Tables 1–5 include data on protein abundance, SNOR sequence conservation analysis, quantitative PCR primers, and *S. pombe* strains used in this study.

Reporting Summary

Peer Review file

Supplementary Video 1 This video presents a 3D rendering of mitochondria and hibernating ribosomes, highlighting SNOR-bound complexes both on and off the OMM.

## Figures and Tables

**Extended Data Table 1:**
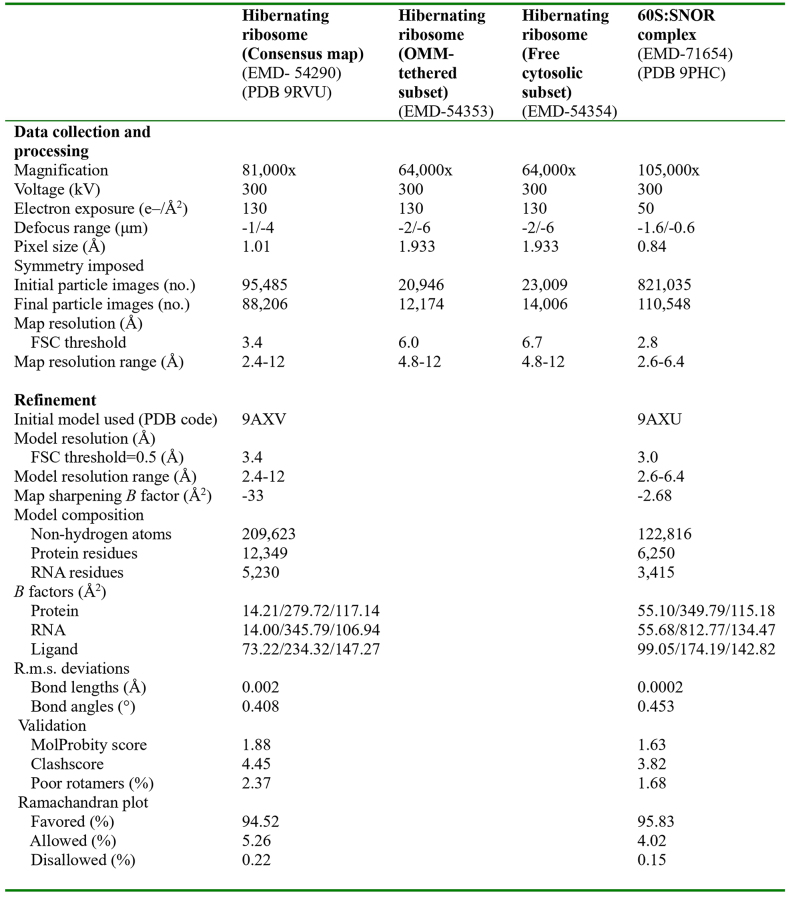
Cryo-ET/Cryo-EM data collection, refinement and validation statistics

Data collection, refinement and validation statistics of the hibernating ribosome (consensus map, EMD-54290, PDB 9RVU), OMM-tethered hibernating ribosome subset (EMD-54353), free cytosolic hibernating ribosome subset (EMD-54354) and in vitro reconstituted 60S:SNOR complex (EMD-71654, PDB 9PHC).

## Data Availability

Cryo-EM maps and model coordinates are deposited in the Electron Microscopy Data Bank (EMDB) as EMD-54290 and in the Protein Data Bank (PDB) as 9RVU for the in situ consensus cryo-ET hibernating ribosome, EMD-54353 and EMD-54354 for the cryo-ET mitochondria-tethered and cytosolic hibernating ribosome, respectively, and EMD-71654 and 9PHC for the cryo-EM structure of the *S. pombe* 60S–SNOR complex. Mass spectrometry and gene conservation data are available on Figshare (10.6084/m9.figshare.31350286 (ref. ^33^)). Reference genomes are available at https://github.com/cassprince/SNOR_conservation. All other data are available in the main text or the supplementary materials and source data.
