# Supplementary material for: SNOR promotes translation restart after dormancy
Source: Nature. 2026 May 13;655(8122):516–24. doi: 10.1038/s41586-026-10530-7 (PMC13345908; doi:10.1038/s41586-026-10530-7)
Supplement: Supplementary file 1 — Supplementary Figs. 1–9 include uncropped immunoblot images, supportive SNOR expression and co-pelleting assays, polysome profiles, viability assays, and structural comparisons. Supplementary Tables 1–5 include data on protein abundance, SNOR sequence conservation analysis, quantitative PCR primers, and S. pombe strains used in this study. [file 41586_2026_10530_MOESM1_ESM.pdf]

nature portfolio

https://doi.org/10.1038/s41586-026-10530-7

Supplementary information

SNOR promotes translation restart after dormancy

In the format provided by the
authors and unedited

a

Figure 2c raw uncropped western blot

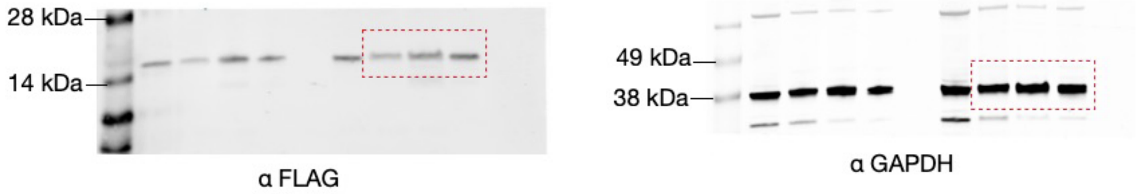

b

Figure 2d raw uncropped western blot

Figure 2d raw uncropped western blot replicate

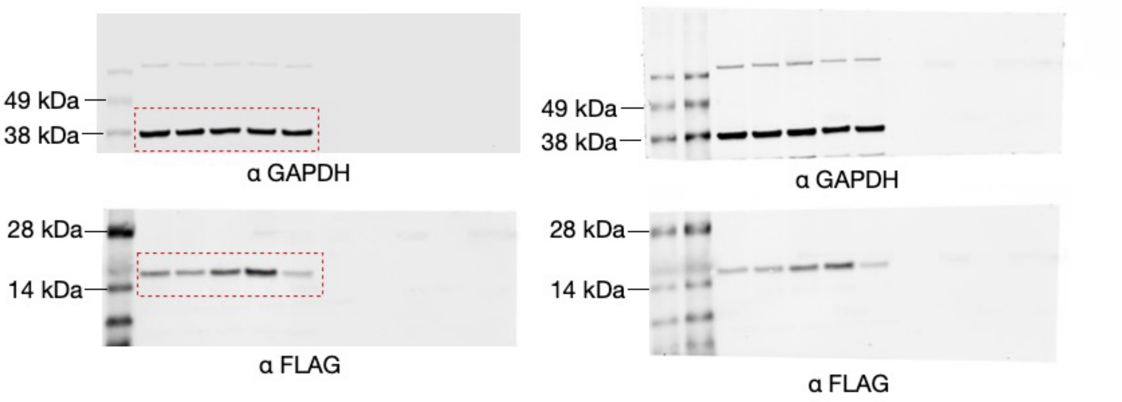

c

Figure 2e raw uncropped western blot

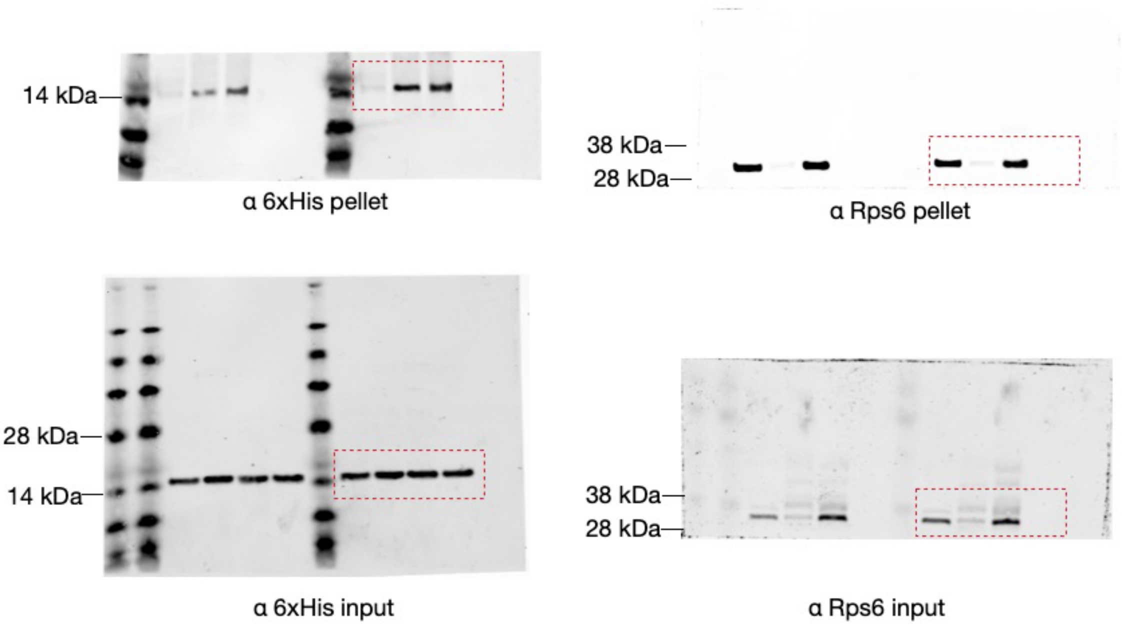

d

Figure 3h raw uncropped western blot

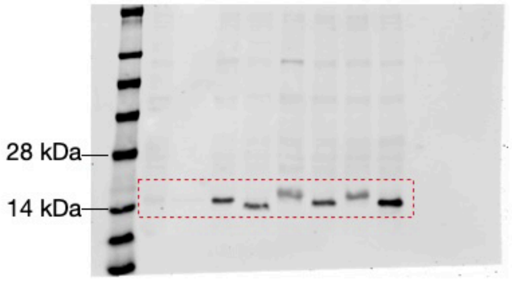

α 6xHis pellet

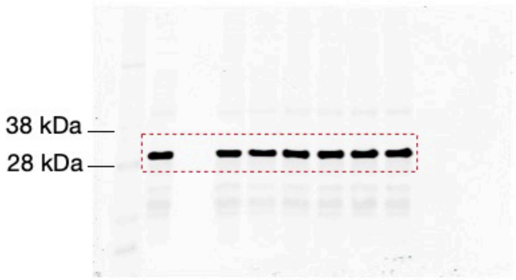

α Rps6 pellet

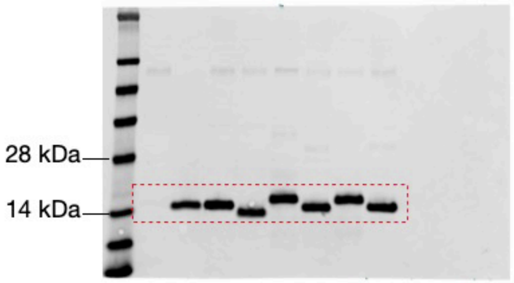

α 6xHis input

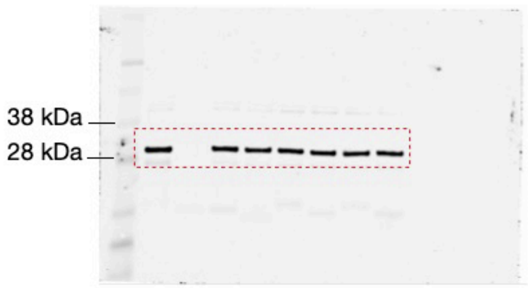

α Rps6 input

Figure 3h raw uncropped western blot replicate

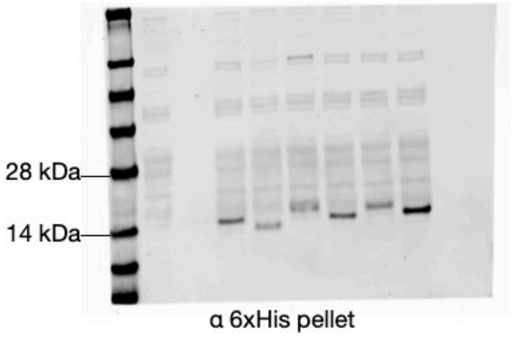

α 6xHis pellet

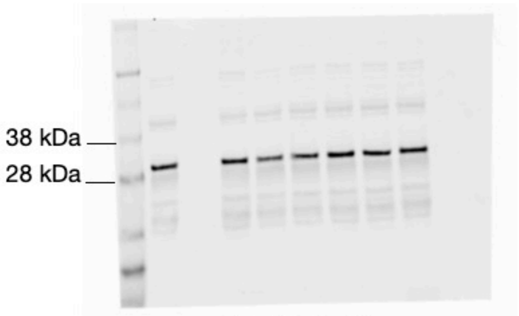

α Rps6 pellet

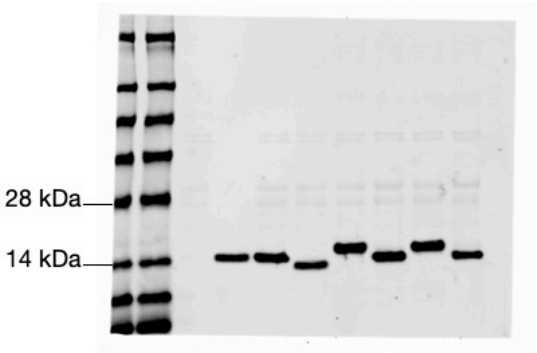

α 6xHis input

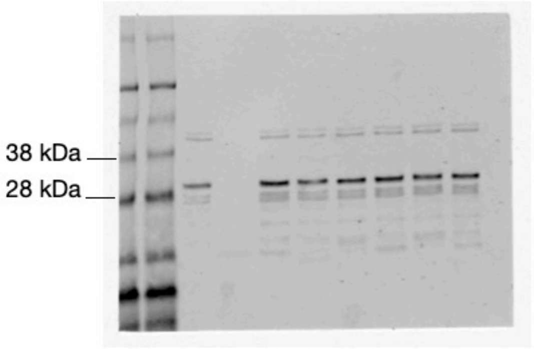

α Rps6 input

e

Figure 4e raw uncropped western blot

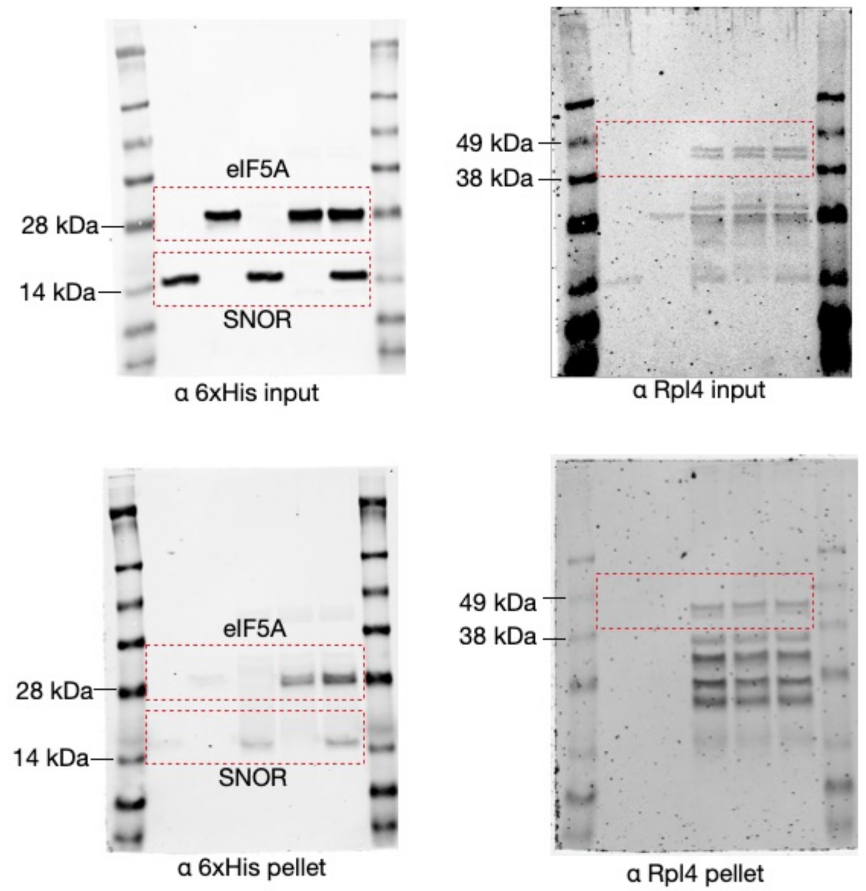

Figure 4e raw uncropped western blot replicate

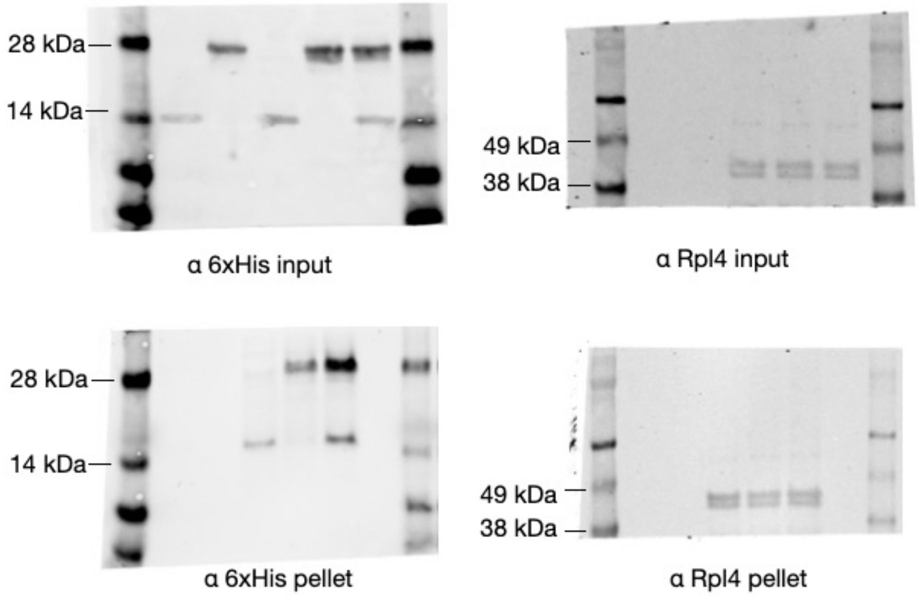

f

Figure 4f raw uncropped western blot

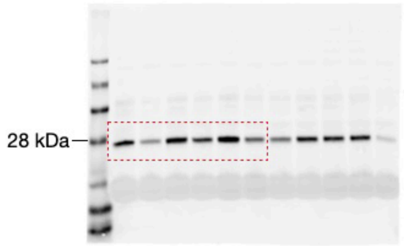

α FLAG reporter

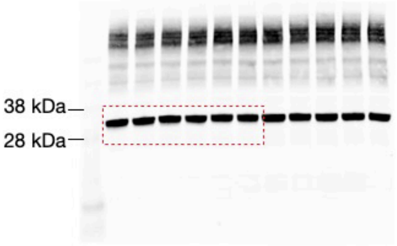

α Rps6 loading control

g

Supplementary Figure 2 raw uncropped western blot

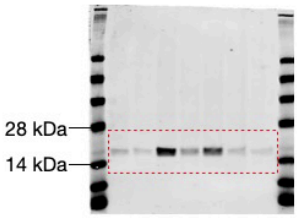

α FLAG

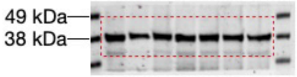

α GAPDH

Supplementary Figure 2 raw uncropped western blot replicate

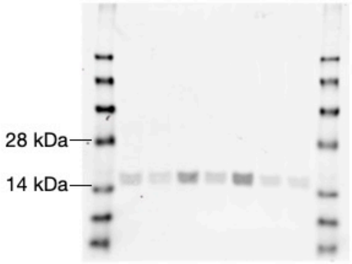

α FLAG

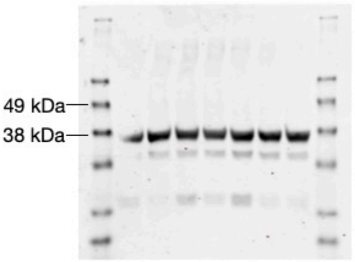

α GAPDH

h

Supplementary Figure 4 raw uncropped western blot

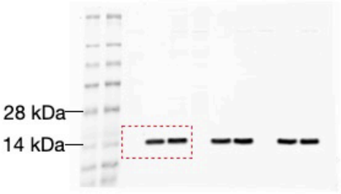

α 6xHis Input

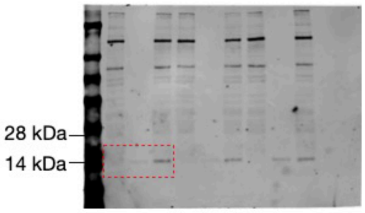

α 6xHis Pellet

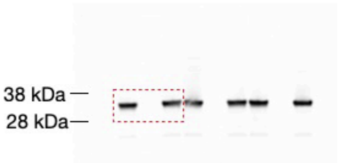

α Rps6 Input

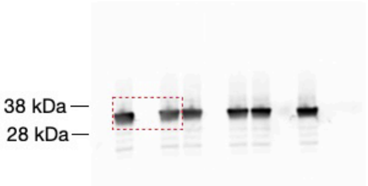

α Rps6 Pellet

Supplementary Data Figure 1. Uncropped images of western blots shown in this study

(a) Immunoblots corresponding to Fig. 2c showing SNOR protein levels in *S. pombe* cell lysates collected after 1, 4, and 7 days of glucose depletion. SNOR was detected using an anti-FLAG antibody; GAPDH served as a loading control. The red dashed box indicates the region displayed in the corresponding main figure panel. Replicate shown on the same gel.

(b) Immunoblots corresponding to Fig. 2d showing SNOR protein levels in *S. pombe* cell lysates exposed to the indicated stress conditions for 2 h prior to lysis. SNOR was detected using an anti-FLAG antibody; GAPDH served as a loading control. The red dashed box indicates the region displayed in the corresponding main figure panel.

(c) Immunoblots of ribosome co-sedimentation assays showing SNOR association with purified 40S, 60S, and 80S ribosomal particles. SNOR was detected via His tag antibody; Rps6 served as a ribosomal loading control. The red dashed box indicates the region displayed in the corresponding main figure panel. Replicate shown on the same gel.

(d) Immunoblots corresponding to Fig. 3h showing ribosome co-sedimentation assays assessing interactions of wildtype SNOR and SNOR mutants with purified *S. pombe* 80S ribosomes. SNOR was detected via His tag antibody; Rps6 served as a loading control. The red dashed box indicates the region displayed in the corresponding main figure panel.

(e) Immunoblots corresponding to Fig. 4e showing ribosome co-sedimentation assays demonstrating cooperative co-binding of SNOR and eIF5A to ribosomes. SNOR and eIF5A were detected via His tag antibody; Rpl4 served as a ribosomal loading control. The red dashed box indicates the region displayed in the corresponding main figure panel.

(f) Immunoblots corresponding to Fig. 4f showing in vitro translation assays using rabbit reticulocyte lysate (RRL), comparing FLAG-tagged reporter expression in the presence of wildtype SNOR, SNOR mutants, and/or eIF5A relative to a BSA control. Rps6 served as a loading control. The red dashed box indicates the region displayed in the corresponding main figure panel. Replicate shown on the same gel.

(g) Immunoblots corresponding to Supplementary Fig. 2 showing SNOR levels in *S. pombe* cell lysates exposed to various stress conditions for 2 h prior to lysis. SNOR was detected using an anti-FLAG antibody; GAPDH served as a loading control. The red dashed box indicates the region displayed in the corresponding supplementary figure panel.

(h) Immunoblots corresponding to Supplementary Fig. 4 showing an in vitro binding assay between SNOR and ribosomes isolated from HEK293 cells. SNOR was detected via its His tag antibody; Rps6 served as a loading control. The red dashed box indicates the region displayed in the corresponding supplementary figure panel. Replicate shown on the same gel.

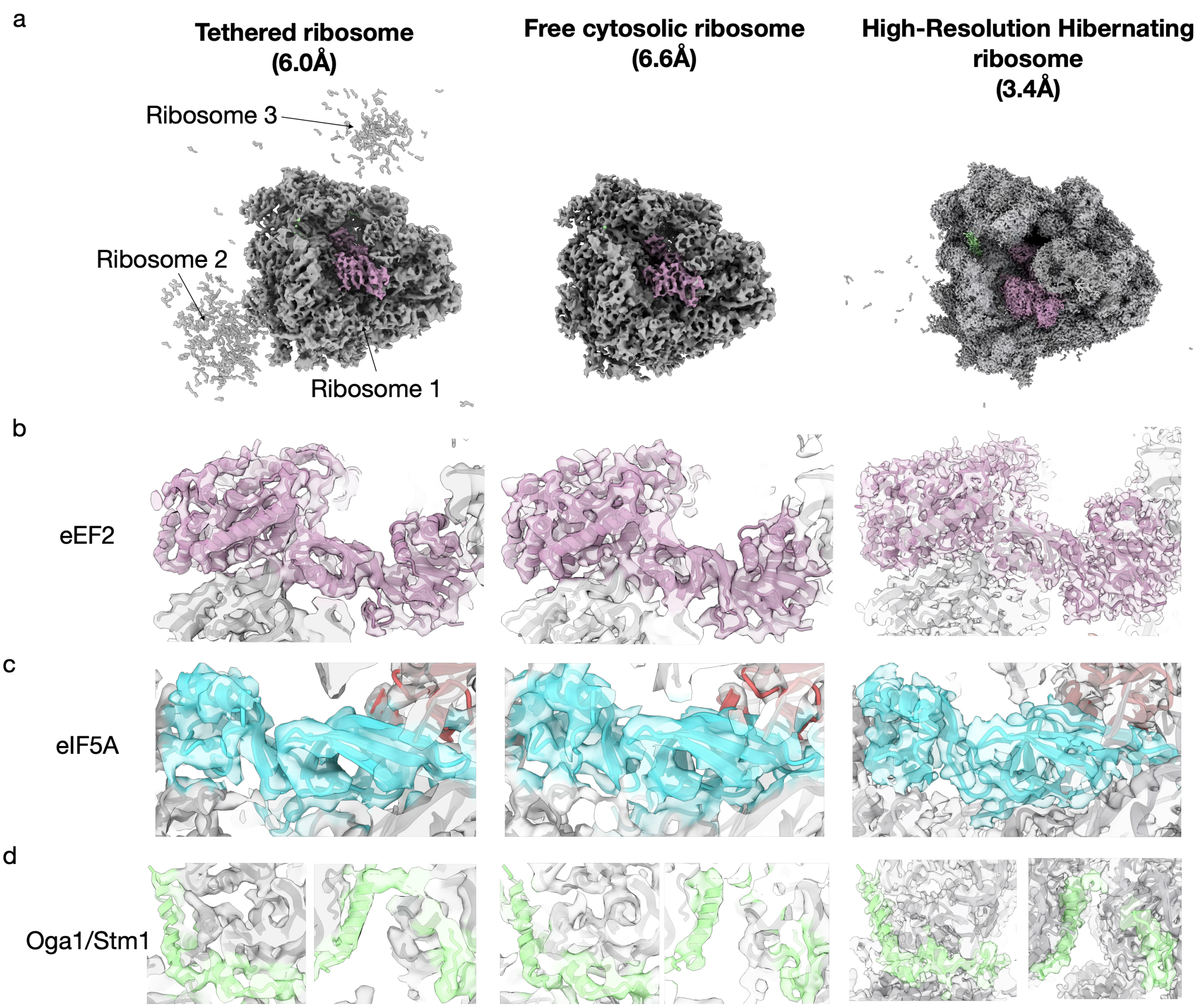

Supplementary Data Figure 2. Densities of hibernation factors bound to the ribosomes in the OMM-tethered and free cytosolic ribosome maps.

(a) Reconstructions of the *S. pombe* hibernating ribosomes obtained by subtomogram averaging of OMM-tethered (left) and free cytosolic (center) and consensus (right) ribosome particles. Maps shown as surface, densities corresponding to observed hibernation factors shown in color. (b-d) Close-ups of densities corresponding to the observed hibernation factors bound to OMM-tethered ribosomes (left), free cytosolic ribosomes (center) and consensus (right). eEF2 shown in purple, eIF5A shown in teal, Oga1/Stm1 shown in green. Atomic models shown as cartoon, density maps shown as surface.

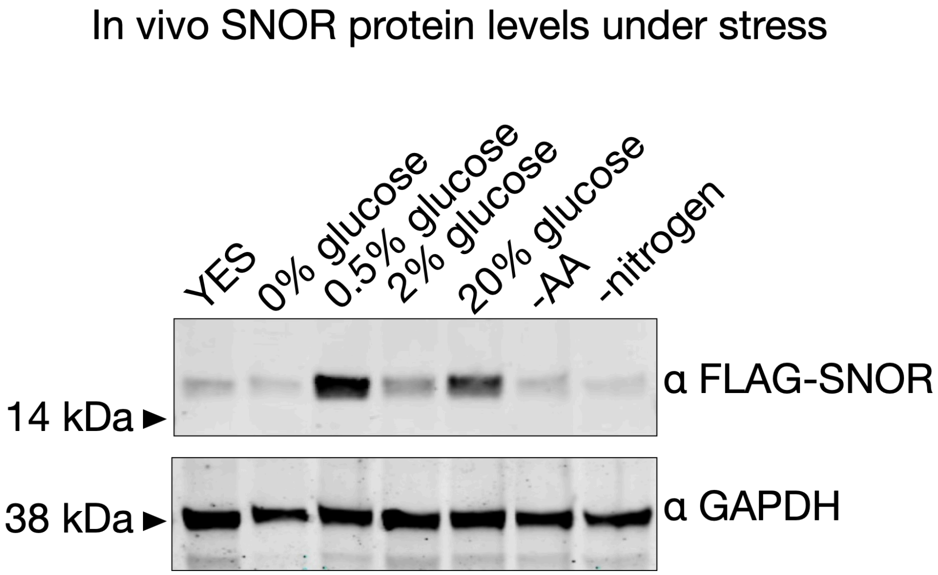

Supplementary Data Figure 3. In vivo SNOR protein levels under stress.

Immunoblot analysis of SNOR levels in *S. pombe* cell lysates exposed to various stress conditions for 2 hours before cell lysis. SNOR was detected using anti-FLAG antibody; GAPDH served as a loading control.

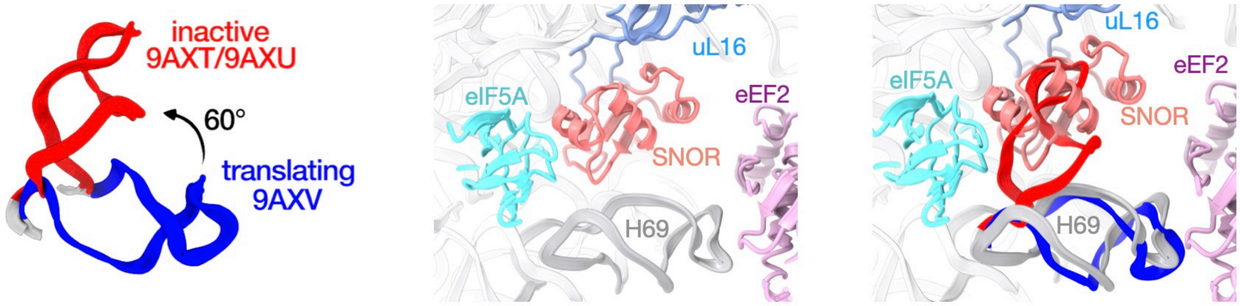

Supplementary Data Figure 4. SNOR binding stabilizes H69 in an outward conformation in dormant ribosomes.

Comparison of H69 conformations across inactive, translating, and hibernating ribosome states. Left: Overlay of H69 from inactive ribosomes (PDB IDs: 9AXT/9AXU; red) and translating ribosomes (PDB ID: 9AXV; blue), illustrating the conformational shift previously observed at low resolution. Middle: H69 position observed in hibernating ribosomes bound to SNOR (coral), eIF5A (teal) and eEF2 (purple) (9RVU - this study). Right: Superposition of SNOR-bound hibernating ribosomes (9RVU - this study; grey) with inactive and translating states. SNOR binding maintains H69 in an outward conformation, whereas inward positioning of H69 observed in the absence of SNOR would sterically overlap with the SNOR binding site.

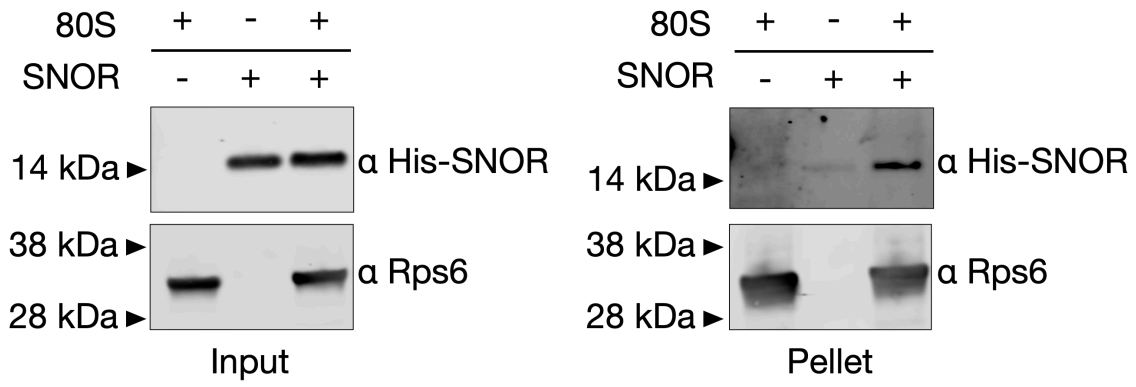

Supplementary Data Figure 5. SNOR binds to mammalian ribosomes.

Immunoblot analysis of an in-vitro binding assay between SNOR and ribosomes isolated from HEK293 cells. Input and pellets after centrifugation though a 30% sucrose cushion are shown. SNOR was detected using anti-His antibody; Rps6 served as a loading control.

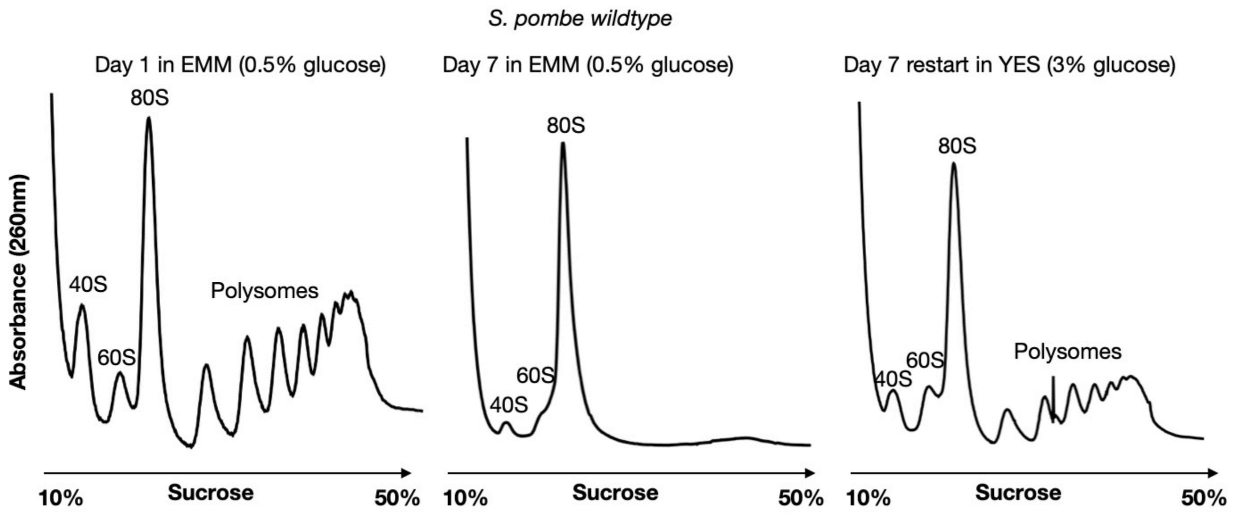

Supplementary Data Figure 6. Protein synthesis restart after glucose reintroduction.

Wildtype *S. pombe* cells (strain 972) were grown in low-glucose EMM (0.5%) for 7 days. Cells were harvested on days 1 and 7, lysed, and subjected to sucrose gradient centrifugation and then fractionated to analyze polysome profiles. By day 7, the profile shifted toward 80S monosomes, indicating a shutdown of protein synthesis, as previously described. Switching the medium to YES and incubating the cells for 2 hours led to the reappearance of polysomes, indicating a restart of protein synthesis.

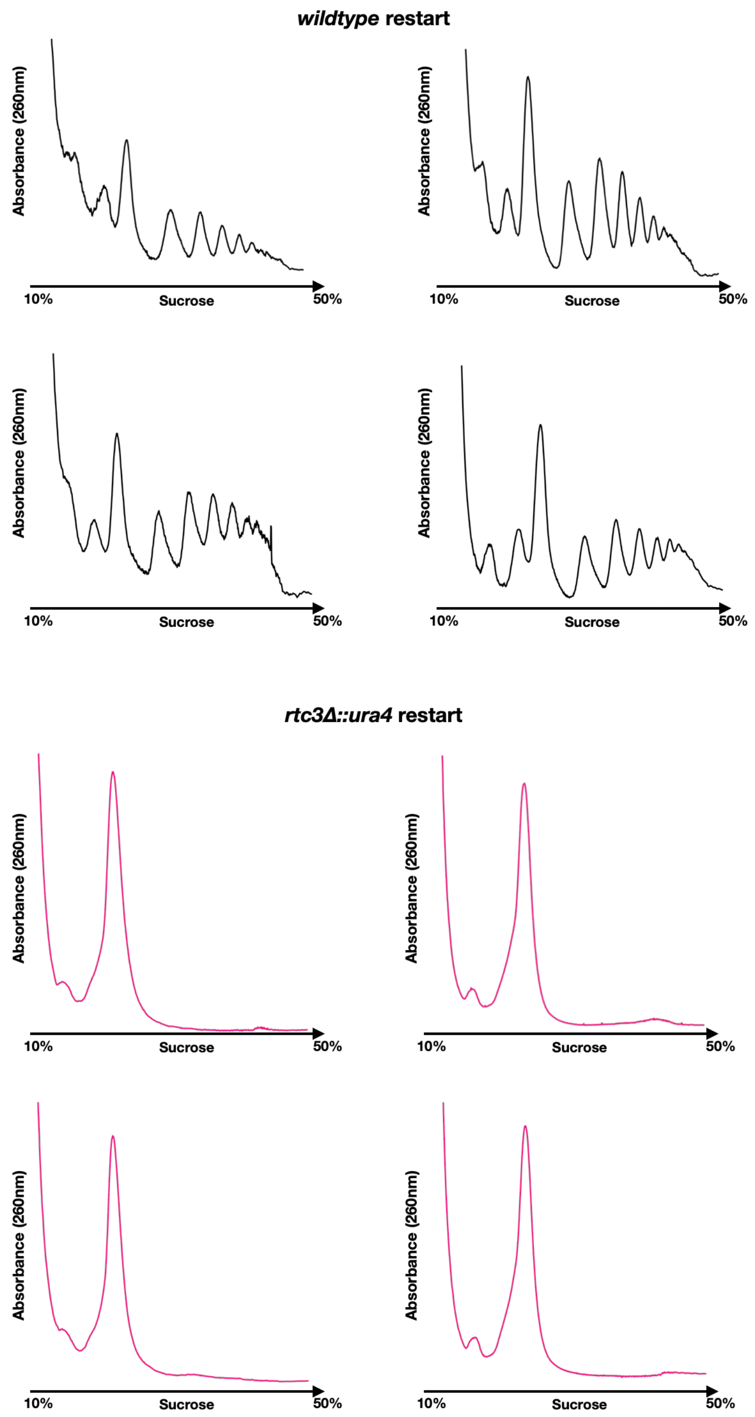

Supplementary Data Figure 7. SNOR knockout cells fail to restart protein synthesis after glucose depletion.

Independent biological replicates of polysome gradient profiles from *S. pombe* cells following glucose reintroduction after 7 days of glucose depletion. Profiles from wildtype cells are shown in black, while profiles from *rtc3*Δ*::ura4* cultures are shown in pink.

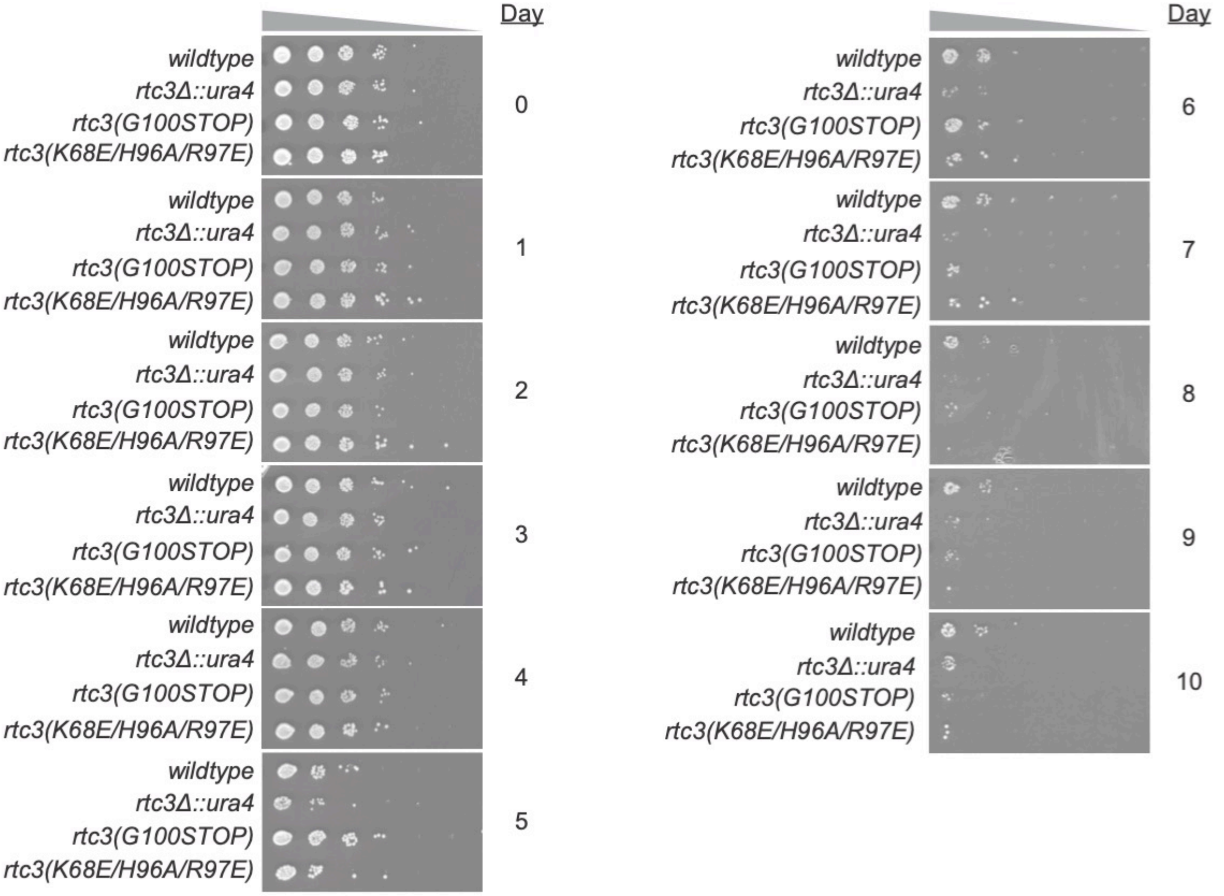

Supplementary Data Figure 8. SNOR mutant strains show pronounced viability defect following glucose depletion.

Serial dilution spot assays of *S. pombe* wildtype and *rtc3* mutant cultures grown to saturation in EMM with 0.5% glucose at 32˚C and then continuously incubated in the same media for up to 10 days. 10-fold serial dilutions of the normalized samples at OD_{595} of 0.25 were spotted on EMM plates containing 2% glucose and incubated at 32˚C. Day 1 corresponds to the 24 h time point of continuous incubation.

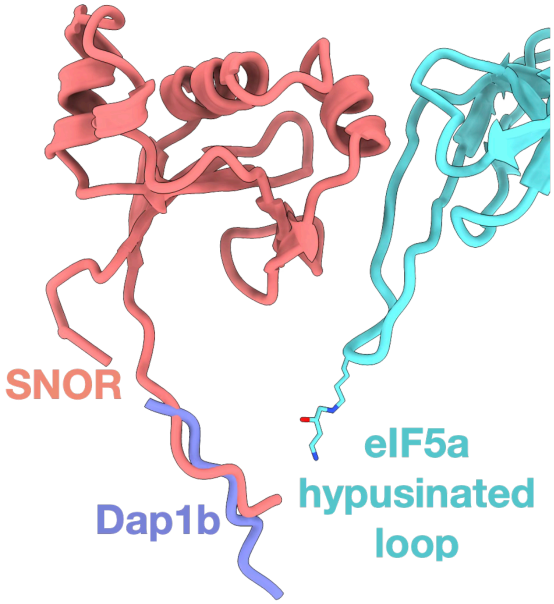

Supplementary Data Figure 9. Comparison of Dap1b binding and SNOR capping at the ribosome exit tunnel.

SNOR model shown in coral, eIF5A model shown in teal (this study), Dap1b shown in light purple (PDB ID:7OYA.) All models shown as cartoon.

Gene name	Protein name	Normalized avg intensity (Day 7 / Day 1)
eft201	Elongation factor 2	**18.67**
**SPBC21C3.19**	**SDO1-like protein C21C3.19 (SNOR)**	**3.56**
cdb4	Curved DNA-binding protein (Ebp1 homolog)	**5.75**
SPBC16A3.08c	Oga1 / Stm1	**0.45**
tif51a	eIF5A	**0.07**

Supplementary Table 1. Normalized average protein intensities based on unbiased mass spectrometry analysis of ribosomes isolated at Day 7 relative to Day 1.

**Phylum**	**Total Genomes**	**Genomes with SNOR**	**% Genomes with SNOR**
Ascomycota	1343	1264	94.1
Basidiomycota	474	412	86.9
Blastocladiomycota	8	2	25.0
Chytridiomycota	65	35	53.8
Cryptomycota	2	1	50.0
Microsporidia	46	1	2.2
Mucoromycota	130	111	85.4
Olpidiomycota	1	0	0.0
Zoopagomycota	179	126	70.4
Total	2248	1952	86.8

Supplementary Table 2. Number of fungal genomes with SNOR across phyla.

Query_accession	Sequence
SPBC21C3.19.1	MSSSKANQTRVCYQPEDTTFIIIASNGPDVMRWRKDKTVPLTEIVDSFQVFTTSNNKGNEGQLITASKQELENTFGTSKDVDVVTKILTDGKII
NP_011955.1	MSTVTKYFYKGENTDLIVFAASEELVDEYLKNPSIGKLSEVVELFEVFTPQDGRGAEGELGAASKAQVENEFGKGKKIEEVIDLILRNGKPNSTTSSLKTKGGNAGTKAYN
XP_013021238.1	MSSGPANQTRVVCQTDIASFVVIGASENIIKSWRTDKTIPLTEVVDSFQVFTLTKGSEGELFKASKQQLENAFGTSKDVDVCAKILSEGKISPH
XP_013019791.1	MSSGPANQTRVYYQTDVASFVIIAASEKDVNNWRSDKTIPLTEVVDSFQIFSLNKGSEGELAKASKLELENAFGTSKDVDVCSKILSEGKITPH
XP_033766758.1	MSTVTKYFYKGENTDLIVFATSEELVDEYLKNPSIGKLSEVVEIFEVFTPQDGRGAEGELGAASKAQVENEFGKGKKIEEVIDLILRNGKPNSTTSSLKTKGGNAYK
XP_022676094.1	MSSPIKYFYKGEETDFIIFVNSEEKVQDYLKNSNINNLTEAVSLFKVFANQDARGSEGELGEASKSQIENEFGPKKTTEEVLDLILKNGKPLSS

Supplementary Table 3. Query sequences used for HMMER profile building.

Primer	Sequence (5’-3’)
Act1 Fwd	AAGTACCCCATTGAGCACGG
Act1 Rev	TCTCACGGTTGGATTTGGGG
Rtc3 Fwd	ATCATCGCCTCAAACGGTCC
Rtc3 Rev	TTGTGCTTGCCATGTTCACG
Stm1 Fwd	GAAAACCGCTGCTTCTCGTG
Stm1 Rev	GCTTCTTTGCCTTCACGAGC
eIF5A Fwd	GAACGGCCACGTCGTGATTA
eIF5A Rev	GAGCTCACCTTCGGGAAGAC
eEF2 Fwd	ACTTGCGTTCTTGCCGTTTC
eEF2 Rev	AGAAAACGGGCTCCTGGATG

Supplementary Table 4. Real-time qPCR primer sequences.

Strain	Genotype	Source
AJY19	*912 ura4-294 leu1-32 h⁻*	gift Dr. Henry Levin (NIH)
AJY23	*2xFLAG-rtc3 ura4-294 leu1-32 h⁻*	This study
KGY28	*972 h⁻*	Lab stock
KGY45	*975 h⁺*	Lab stock
KGY10138-2	*rtc3Δ::ura4⁺ ade6-M210 leu1-32 ura4-D18 h⁻*	This study
KGY2418-2	*rtc3Δ::kanMX6 ade6-M21X ura4-D18 leu1-32 h⁺*	This study
KGY2215-2	*rtc3(1-99) ade6-M210 leu1-32 ura4-D18 h⁻*	This study
KGY567-2	*rtc3-K68E,H96A,R97E ade6-M210 leu1-32 ura4-D18 h⁻*	This study
KGY6063-2	*rtc3Δ::ura4⁺ h⁺*	This study
KGY2177-2	*rtc3(1-99) h⁺*	This study
KGY6071-2	*rtc3-K68E,H96A,R97E h⁺*	This study

Supplementary Table 5. *S. pombe* strains used in this study
