# Supplementary material for: SNOR promotes translation restart after dormancy
Source: Nature. 2026 May 13;655(8122):516–24. doi: 10.1038/s41586-026-10530-7 (PMC13345908; doi:10.1038/s41586-026-10530-7)
Supplement: Supplementary file 2 — Reporting Summary [file 41586_2026_10530_MOESM2_ESM.pdf]

nature portfolio

Corresponding author(s): Simone Mattei, Ahmad Jomaa

Last updated by author(s): Mar 24, 2026

Reporting Summary

Nature Portfolio wishes to improve the reproducibility of the work that we publish. This form provides structure for consistency and transparency in reporting. For further information on Nature Portfolio policies, see our Editorial Policies and the Editorial Policy Checklist.

Statistics

For all statistical analyses, confirm that the following items are present in the figure legend, table legend, main text, or Methods section.

n/a	Confirmed	
☐	☒	The exact sample size (n) for each experimental group/condition, given as a discrete number and unit of measurement
☐	☒	A statement on whether measurements were taken from distinct samples or whether the same sample was measured repeatedly
☐	☒	The statistical test(s) used AND whether they are one- or two-sided<br>*Only common tests should be described solely by name; describe more complex techniques in the Methods section.*
☒	☐	A description of all covariates tested
☐	☒	A description of any assumptions or corrections, such as tests of normality and adjustment for multiple comparisons
☐	☒	A full description of the statistical parameters including central tendency (e.g. means) or other basic estimates (e.g. regression coefficient) AND variation (e.g. standard deviation) or associated estimates of uncertainty (e.g. confidence intervals)
☐	☒	For null hypothesis testing, the test statistic (e.g. F, t, r) with confidence intervals, effect sizes, degrees of freedom and P value noted<br>*Give P values as exact values whenever suitable.*
☒	☐	For Bayesian analysis, information on the choice of priors and Markov chain Monte Carlo settings
☒	☐	For hierarchical and complex designs, identification of the appropriate level for tests and full reporting of outcomes
☒	☐	Estimates of effect sizes (e.g. Cohen's d, Pearson's r), indicating how they were calculated

Our web collection on statistics for biologists contains articles on many of the points above.

Software and code

Policy information about availability of computer code

Data collection	SerialEM 3.8 for cryo-EM SPA acquisition. MAPS 3.8 for cryo-SEM and FIB acquisition. SerialEM 4.2 and SPACEtomo 1.0 for cryoET acquisition.
Data analysis	CryoSPARC 4.6.0 and RELION3 for single-particle analysis. COOT 0.9.8.95 and PHENIX 1.20.1 for model building. RELION 4.0.1 for frame conversion, refinement and classification. Warp 1.09 and 2.0.0dev36; and AreTomo2/1.1.2 and Aretomo3/2.2.2 for alignment and reconstruction. PyTom 1.1 and 0.7.2 for ribosome picking. Dragonfly 2024.1 with a 2.5D U-Net for membrane segmentation and mask generation. ArtiaX for particle curation. M for multi-particle refinement. CryoDRGN-ET for heterogeneity analysis. ChimeraX 1.9, Blender 4.2 with Microscopy Nodes and Molecular Nodes 2.6.1 for visualization.

For manuscripts utilizing custom algorithms or software that are central to the research but not yet described in published literature, software must be made available to editors and reviewers. We strongly encourage code deposition in a community repository (e.g. GitHub). See the Nature Portfolio guidelines for submitting code & software for further information.

Data

Policy information about availability of data

All manuscripts must include a data availability statement. This statement should provide the following information, where applicable:

- Accession codes, unique identifiers, or web links for publicly available datasets
- A description of any restrictions on data availability
- For clinical datasets or third party data, please ensure that the statement adheres to our policy

Cryo-EM maps and model coordinates are deposited in the EMDB as EMD-54290 and in the PDB as PDB ID 9RVU for the in-situ consensus cryo-ET hibernating ribosome, and EMD-54353 and EMD-54354 for the cryo-ET mitochondria-tethered and free cytosolic free hibernating ribosome, respectively, and EMD-71654 and in the PDB as PDB ID 9PHC for the cryo-EM structure of the S. pombe 60S:SNOR ribosomes. Model coordinates for previously determined S. pombe translating and non-translating ribosomes used for initial docking into the observed densities are deposited in the PDB as PDB ID 9AXU and 9AXV, respectively. All other data are available in the main text or the supplementary materials.

Research involving human participants, their data, or biological material

Policy information about studies with human participants or human data. See also policy information about sex, gender (identity/presentation), and sexual orientation and race, ethnicity and racism.

Reporting on sex and gender	N/A
Reporting on race, ethnicity, or other socially relevant groupings	N/A
Population characteristics	N/A
Recruitment	N/A
Ethics oversight	N/A

Note that full information on the approval of the study protocol must also be provided in the manuscript.

Field-specific reporting

Please select the one below that is the best fit for your research. If you are not sure, read the appropriate sections before making your selection.

☒ Life sciences ☐ Behavioural & social sciences ☐ Ecological, evolutionary & environmental sciences

For a reference copy of the document with all sections, see nature.com/documents/nr-reporting-summary-flat.pdf

Life sciences study design

All studies must disclose on these points even when the disclosure is negative.

Sample size	No statistical methods were used to predetermine EM and ET sample size. Instead, objective data quality parameters, including B-factors from comparable cryo-EM and cryo-ET studies, were used to estimate the number of particles, tilt-series and images required to achieve the target resolution. Based on these estimates, data acquisition was planned to reach a resolution sufficient for reliable structural interpretation, including side-chain resolution where applicable.<br>For sub-tomogram averaging, 1,012 tilt-series acquired from lamellae of S. pombe cells deprived of glucose for 7 days were analyzed, yielding 88,206 sub-tomograms selected by template matching, contributed to the final 3D reconstruction. For single-particle analysis of the in vitro reconstituted 60S:SNOR complex, 7,277 micrographs were analyzed from which a subset of 110,548 picked particles, picked using a Laplacian blob-based picker in RELION, exhibiting best defined SNOR density contributed to the final 3D map. Biochemical experiments were performed in at least two independent replicates with consistent results.
Data exclusions	No data was excluded
Replication	All experiments were successfully replicated. Biochemical assays were performed in at least two independent experiments, and polysome gradient profiling was performed in at least three independent experiments.
Randomization	Resolution was estimated from Fourier shell correlation (FSC) curves using the gold-standard approach
Blinding	Blinding was not applicable, as this study did not involve clinical or human research.

Reporting for specific materials, systems and methods

We require information from authors about some types of materials, experimental systems and methods used in many studies. Here, indicate whether each material, system or method listed is relevant to your study. If you are not sure if a list item applies to your research, read the appropriate section before selecting a response.

Materials & experimental systems

n/a	Involved in the study
☐	☒ Antibodies
☐	☒ Eukaryotic cell lines
☒	☐ Palaeontology and archaeology
☒	☐ Animals and other organisms
☒	☐ Clinical data
☒	☐ Dual use research of concern
☒	☐ Plants

Methods

n/a	Involved in the study
☒	☐ ChIP-seq
☒	☐ Flow cytometry
☒	☐ MRI-based neuroimaging

Antibodies

Antibodies used

FLAG (Genscript, cat no. A00187), 1:4000 dilution
6xHis (Genscript, cat no. A00186), 1:4000 dilution
Rps6 (Cell Signaling, cat. no. 2217),1:2000 dilution
GAPDH (Proteintech, cat no. 60004-1-Ig), 1:5000 dilution
Rpl4 (Invitrogen, cat. no MA5-56865), 1:2000 dilution
Goat anti-Rabbit IgG (H+L) (Invitrogen, cat. no A-11034), 1:10000 dilution
Goat anti-Mouse IgG (H+L) (Invitrogen, cat. no A-21058), 1:10000 dilution

Validation

Primary antibodies were validated by the manufacturers for Western blot applications, as indicated in the respective datasheets. Anti-FLAG (GenScript, A00187) and anti-6×His (GenScript, A00186) antibodies recognize engineered epitope tags. Anti-Rps6 (Cell Signaling Technology, 2217), anti-Rpl4 (Invitrogen, MA5-56865) and anti-GAPDH (Proteintech, 60004-1-Ig) antibodies are widely used for Western blotting and showed bands at the expected molecular weights in this study. Secondary antibodies (Invitrogen, A-11034 and A-21058) were used according to manufacturer recommendations and showed the expected species-specific reactivity with their respective primary antibodies (anti-rabbit and anti-mouse, respectively).

Eukaryotic cell lines

Policy information about cell lines and Sex and Gender in Research

Cell line source(s)

S. pombe strain 972 h-, S. pombe strain 912 h- ura4-294 leu1-32

Authentication

ATCC, Bioneer, all strains were validated by whole-cell PCR using specific sequences.

Mycoplasma contamination

Schizosaccharomyces pombe strains used in this study were not tested for mycoplasma contamination

Commonly misidentified lines
(See ICLAC register)

No commonly misidentified lines were used in this study

Plants

Seed stocks

N/A

Novel plant genotypes

N/A

Authentication

N/A
