# Supplementary material for: SNOR promotes translation restart after dormancy
Source: Nature. 2026 May 13;655(8122):516–24. doi: 10.1038/s41586-026-10530-7 (PMC13345908; doi:10.1038/s41586-026-10530-7)
Supplement: Supplementary file 3 — Peer Review file [file 41586_2026_10530_MOESM3_ESM.pdf]

nature portfolio

Peer Review File

SNOR promotes translation restart after dormancy

Corresponding Author: Professor Ahmad Jomaa

Any redactions in this file are there to maintain patient confidentiality, the confidentiality of unpublished data, or to remove third-party material.

This file contains all reviewer reports in order by version, followed by all author rebuttals in order by version.

Attachments originally included by the reviewers as part of their assessment can be found at the end of this file on pages 21-22.

Version 1:

Reviewer comments:

Referee #1

(Remarks to the Author)
The paper by Gluc et al. identifies a novel stress factor responsible for adaptation of Schizosaccharomyces pombe to altered glucose levels. Cryo-ET structural studies of glucose-depleted yeast cells revealed hibernating ribosomes associated, besides the previously known hibernation factors, with a protein with an unknown function, Rtc3 (renamed by authors as SNOR). The authors establish that SNOR expression is induced by altered glucose levels and show that this factor associates with the 80S ribosome to shut down cellular translation in the stress conditions. Cryo-EM structural and biochemical analysis reveal that SNOR binds via its N-terminal globular domain at the interface side of the large ribosomal subunit near the peptidyl-transferase center, positioning its C-terminal tail within the nascent peptide exit tunnel, thereby excluding peptidyl-tRNA from the ribosome. The authors further show that SNOR facilitates restarting cellular translation after stress-induced dormancy.
Critique
This is an important study that extends our understanding of how eukaryotes regulate global cellular translation in response to environmental stress. The structural and biochemical analyses are of high quality. The experiments are convincing and properly controlled. There are several aspects however, that require clarification.
Specifically:

1. To assign extra density associated with hibernating ribosomes in glucose-depleted cells, the authors use fairly indirect structure-based approaches. Although there is nothing wrong with this, the mass spectrometry analysis seems a more direct way to identify the ribosome-associated protein factors. Was such analysis performed? If yes, what were the results? If no, maybe it should be done.
2. Authors discuss interaction of SNOR with and important and still enigmatic eIF5A (Fig. 4a,b). It would be really interesting to know whether SNOR and eIF5A show binding cooperativity. The established in vitro binding assay described in the paper (Figure 2c) could answer this question.
3. Cessation of translation may lead to degradation of rRNA in the dormant ribosome and one of the proposed roles of hibernation factors is protection from such degradation. It would be a simple but informative experiment to compare the rRNA integrity in the wt and SNOR-depleted cells over time during cessation of translation caused by glucose depletion.
There are several more minor points:
- ll. 142-143. Fig. 2a does not seem to support the statement that “rtc3 transcript upregulation being the most prominent among the analyzed hibernation factors” . Upregulation of eft201 and possibly of tif51 seem to be somewhat more pronounced than that of rtc3.
- ll. 152-153 and Fig. 2c. Why is the small subunit protein present in the 60S sample?
- ll. 234-240 and Fig. 4g. While overexpression of SNOR does affect translation, it remains unclear whether this effect is related to the ability of SNOR to bind to the ribosome. Therefore, this experiment lacks the critical control: the effect of the wt SNOR overexpression should be contrasted with that of the inactive SNOR mutant (e.g. K68E/H96A/R97E).
- ll. 248-256. Extended Data Fig. 10 seems unneeded given that the main result is shown in the main Fig. 5b.
- ll. 328-329. It is unclear what authors mean by “potential nascent chain stuck in the tunnel of hibernating ribosome”. Do they mean tRNA-unlinked nascent chain? Because if nascent chain remains linked to the tRNA, then SNOR should not be

able to bind.

Referee #2

(Remarks to the Author)
I co-reviewed this manuscript with one of the reviewers who provided the listed reports.

Referee #3

(Remarks to the Author)
This is a well-executed and carefully interpreted study that identifies SNOR as a fungal ribosome-associated factor linked to hibernation and recovery during glucose starvation. The work provides high-resolution structural insight and compelling biochemical characterization. Its major strengths are the technical rigor and the integration of cryo-ET and cryo-EM data. The main limitation is the lack of a mechanistic explanation for SNOR-dependent recovery, which somewhat restricts the conceptual advance. Nonetheless, the study represents a valuable contribution to understanding ribosome hibernation and nutrient-dependent translational control.

Comments:

The authors previously identified that hibernating 80S ribosomes (lacking mRNA and tRNA) are tethered to the surface of the outer mitochondrial membrane (OMM) through 40S-bound RACK1 upon glucose depletion in S. pombe, using cryo-electron tomography (cryo-ET) (Ref #24). In the present study, the authors improve both the resolution and quality of their cryo-ET data and report structures of OMM-bound and cytoplasmic hibernating ribosomes. As the two structures are highly similar, they were merged to yield a 5.5 Å (overall) / 4.7 Å (large subunit) resolution reconstruction. The structure reveals that the 80S ribosome contains eEF2, Stm1 (Oga1), eIF5A, and an additional density at the peptidyl-transferase center (PTC), without associated tRNA or mRNA.

Through database searches, the authors identified Rtc3 (previously of unknown function) as a candidate for this additional density and proposes to name it SNOR. Interestingly, SNOR expression is upregulated at both transcript and protein levels upon glucose depletion. Purified recombinant SNOR interacts with the 60S and 80S ribosomal subunits but not with 40S. Cryo-EM analysis of a reconstituted SNOR–60S complex (2.7 Å overall, with local resolution of 3.5–5 Å for SNOR) revealed a structure that closely matches the unidentified density seen in cryo-ET. By fitting this cryo-EM structure into the cryo-ET map, the authors show that the SNOR C-terminal tail caps the peptide-exit channel, while the core SBDS-like domain occupies the P-site and prevents tRNA binding. This configuration stabilizes the inactive conformation of H69. Importantly, structure-based mutagenesis affects 60S binding in predictable ways, consistent with the structural model. The proposed interaction bridge involving the L1 stalk, eIF5A, SNOR, and the PTC/H69 region represents an intriguing new mechanism for stabilizing ribosomes in an inactive state.

Biochemical data are used to show that purified S. pombe SNOR interacts with human 80S ribosomes and inhibits in-vitro translation, whereas 60S-binding mutants do not. Interestingly, in S. pombe, SNOR overexpression causes a rather modest (~30%) reduction in the polysome/monosome (P/M) ratio, suggesting that SNOR alone is not able to regulate translation inhibition. Upon glucose depletion, wild-type cells lose polysomes (with a predominant 80S peak), which recover within 2 h of glucose re-addition. In contrast, SNOR knockout (KO) cells exhibit the same initial polysome loss but fail to recover upon glucose replenishment and display reduced viability. The severity of the recovery defect scales with the duration of glucose depletion. SNOR-deficient cells also show altered morphology under these conditions. SNOR expression is induced specifically by glucose limitation but not by amino acid or nitrogen starvation. Homologs of SNOR are conserved in most fungi but are absent in higher eukaryotes.

Overall summary:
The structural and biochemical data are of high quality and convincingly support the conclusion that SNOR associates with hibernating 80S ribosomes upon glucose depletion. The evidence indicates that SNOR itself is not a primary driver of ribosome hibernation since it's overexpression alone has only a modest translational effect. Interestingly, SNOR KO cells still undergo hibernation normally—but the recovery from glucose starvation is impacted. This interpretation is well supported by the data.

The study also extends previous observations of eEF2–Stm1(Oga1)-containing hibernating complexes and connects them to a newly identified ribosome-bound factor. Interestingly, a closely related inactive 80S complex has been reported in Xenopus oocytes (80S containing eEF2, Habp4/Stm1, eIF5A, and Dap1b; ref. 21). Although Dap1b is not homologous to SNOR, it appears functionally analogous, interacting with eIF5A at the PTC/PET. Perhaps the real novelty in the current study lies in the detailed description of the L1-stalk–eIF5A–SNOR bridge, which provides a mechanistic rationale for the stabilization of the L1 stalk in the inactive 80S state.

Unfortunately, the central mechanistic insight (and which the title of the manuscript focuses on)—that SNOR promotes ribosome recovery after glucose depletion—remains speculative. The authors suggest that eIF5A might be involved, but no direct evidence is presented. Given that SNOR function appears specific to glucose levels in fungi, it would be important to elucidate how SNOR connects to mitochondrial metabolism or the signaling events governing ribosome reactivation. Without such mechanistic understanding, the work—though technically strong—may be considered incremental as it

primarily presents another variant of a known hibernating 80S complex with an additional factor identified through cryo-ET.

Specific points:

1. Gene expression analysis: Lines 140–143 state that eft201, tif51, oga1, and rtc3 transcripts are all elevated upon glucose depletion. However, the qPCR data in Fig. 2a do not appear to show a corresponding increase in Oga1 expression. This discrepancy should be clarified.

2. Overexpression assay (Fig. 4g): SNOR overexpression was examined after three days in 0.5% glucose medium, just before hibernation occurs, yielding only a modest reduction in polysomes. Extending the incubation (> 4 days) and testing whether overexpression accelerates or enhances hibernation would strengthen the interpretation. Including the 60S-binding mutant in this assay would also be informative.

3. Recovery assays (Fig. 5b,d): In addition to the SNOR-KO strain, it would be valuable to test the 60S-binding mutant for both polysome recovery and growth rescue, to directly link the structural findings to functional consequences.

Referee #4

(Remarks to the Author)
In this study, Gluc et al. have identified a new ribosomal factor, SNOR, along with the hibernating factors Oga1 eEF2 and eIF5A, using cellular tomography (cryo-ET) of glucose-starved S. pombe cells. Also reported is a single-particle cryo-reconstruction in vitro reconstitution of the 60S ribosome SNOR complex. Furthermore, SNOR functional characterization is carried out by creating SNOR knockout and point mutants, as well as SNOR overexpression.

Authors have improved the resolution of cryo-ET reconstruction from ~11 Å, as reported earlier (Gemin et al., 2024), to ~5.5 Å in the current study. SNOR was not detected in the previous research, most likely due to its low resolution. Essentially, it's a continuation of the earlier study with improved resolution, identifying a new factor, SNOR, and some other factors.

The authors have claimed that the SNOR has a role in translation restart following cellular dormancy. The SNOR binding to glucose-stressed ribosomes appears apparent, but the mechanism of ribosome reactivation is not clearly demonstrated at the molecular level.

I have the following major concerns.
1. The Cryo-ET reconstruction is claimed to be 5.5 Å, and the single particle reconstruction is claimed to be 2.9 Å. However, the cryo density claimed for SNOR is fragmented in both Cryo-ET reconstruction and single-particle reconstruction. Overall map quality is poor; a few screenshots are shown in Figure 1. attached with comments.
At a 2.9 Å resolution, we should be able to see the bulky side-chain residues clearly. None of the interactions between SNOR-60S ribosome, shown in figures, are supported with cryo-EM densities.
SNOR has low occupancy, as evident in cryo-EM reconstruction; only ~10% of particles from 2D classification were finally selected for the 60S-SNOR reconstruction. The FSC shows in the PDB validation report for 60S-SNOR reconstruction is also falling very early.
I suggest improving the occupancy of SNOR in the final reconstruction by increasing the SNOR concentration in the in-vitro reconstruction. Sorting the structural heterogeneity by extensive 3D classification and using partial signal subtraction embedded in RELION.

2. An interesting observation in Gemin et al. 2024 was that H69 adopts a different conformation in the hibernating ribosome. However, in the current manuscript, the authors do not mention this.
A comparison of the current coordinates with the 11 Å cryo-ET reconstruction (PDB ID: 9AXU) is shown in the figure below. It looks like the altered conformation reported earlier overlaps with the SNOR. Shown in Figure 2, attached with comments. Any explanation?

3. As SNOR and some other factors were missed at 11 Å cryo-ET reconstruction, what are the probabilities of missing some small protein in the current 5.5 Å resolution cryo-ET reconstruction? I would suggest performing mass spectrometry to determine if any unknown factors remain unmodeled in cryo-ET maps. That would also confirm that it's SNOR, not another protein having the same domain with disordered regions.

4. The SNOR name is coined because it resembles Domaine 1 of the SBOD protein. If we look carefully, the polarity of SNOR and SBOD DI is opposite. SNOR's C-terminus is inserted into the PTC, whereas the N-terminus of SBOD. Basically, the secondary structure topology will be opposite. Whether this claim same domain is valid? It needs to be supported with literature. Furthermore, does the sequence analysis shown in Figure 3d have any relevance? As the direction of SNOR and SBOD D1 is opposite.

5. The authors have made the SNOR knock-out strain. However, ideally, I would like to create an SNOR complement strain in an SNOR knockout background and conduct all functional studies.

6. SNOR is expressed in glucose concentrations of 0.5% and 20% Whether SNOR expression is glucose-specific or 20% glucose induces osmotic stress to the cell, remains unclear. I would suggest the SNOR expression by replacing 20% glucose with an equimolar concentration of sorbitol, NaCl, etc.
7. All figure qualities can be improved.

Minor points
In line 61: Check if reference 14 is correctly cited; in my understanding, it's not the first report.
The result section from lines 86 to 103 can be made concise, and details can be added to the method section.
Line 143: Extended data table 2 is not correctly cited.
Paragraph 170 -176, Fig. 3C is not cited.
Line 240 under nutrient stress, or it should be under carbon stress.
Extended Data Figure 1: What does M stand for? An expansion is required
Table 1: Data collection and processing table section FSC threshold value missing
Line 509: If the splitting buffer contains 5 mM $MgCl_2$, or lower concentration. Generally, 1 mM $MgCl_2$ is used for splitting.

Version 2:

Reviewer comments:

Referee #1

(Remarks to the Author)
In the revised manuscript, Gluc et al. have added useful details and improved further the paper. The authors have convincingly addressed most of my questions as well as, I believe, the questions and concerns of other referees.
My only remaining issue is related to the last minor point in my original review. I suspect that I am not the only one who would be confused with "nascent polypeptides retained in hibernating ribosomes prior to translation restart". I recommend that authors clarify that they mean the nascent protein unlinked from tRNA (lines 312-314) so that it clarifies that the nascent protein is unlinked from tRNA. I propose the following version of the sentence (ll. 312-314) for the authors' consideration: "The positioning of the SNOR C-terminal tail within the exit tunnel raises the possibility that SNOR senses nascent polypeptides unlinked from tRNA but retained in hibernating ribosomes prior to translation restart.

Referee #2

(Remarks to the Author)
I co-reviewed this manuscript with one of the reviewers who provided the listed reports.

Referee #3

(Remarks to the Author)
The authors have addressed my main comments in this significantly revised manuscript. In particular, I am now more convinced that the interaction between SNOR and eIF5A is important for the restart of translation following stress. This is an interesting study that moves the field forward and I support its publication.

Referee #4

(Remarks to the Author)
The revised manuscript is very much improved. The authors have done additional experiments and analysis. Recollected cryo-ET data and improved the overall resolution to ~3.3 Å. Improved overall cryo- EM map for SNOR. Performed mass spectrometry analysis. Generated SNOR knock-out strains. Performed osmotic stress experiments, etc. Overall, the authors have satisfactorily addressed most of the points and have done a great job.

However, the authors have not mentioned anything about my query. "The authors have claimed that the SNOR has a role in translation restart following cellular dormancy. The SNOR binding to glucose-stressed ribosomes appears apparent, but the mechanism of ribosome reactivation is not clearly demonstrated at the molecular level."

Open Access This Peer Review File is licensed under a Creative Commons Attribution 4.0 International License, which permits use, sharing, adaptation, distribution and reproduction in any medium or format, as long as you give appropriate credit to the original author(s) and the source, provide a link to the Creative Commons license, and indicate if changes were made.
In cases where reviewers are anonymous, credit should be given to 'Anonymous Referee' and the source.
The images or other third party material in this Peer Review File are included in the article's Creative Commons license, unless indicated otherwise in a credit line to the material. If material is not included in the article's Creative Commons license and your intended use is not permitted by statutory regulation or exceeds the permitted use, you will need to obtain permission directly from the copyright holder.
To view a copy of this license, visit https://creativecommons.org/licenses/by/4.0/

Nature manuscript 2025-08-21565A:

We thank the reviewers for their careful and constructive evaluation of our manuscript, and we're encouraged by their assessment that this work represents an important study with high-quality structural and biochemical analyses. We particularly appreciate their thoughtful comments emphasizing the need for deeper mechanistic insight into SNOR function and its interplay with eIF5A during ribosome restart.

In response, we undertook extensive additional structural, biochemical, and functional analyses that substantially strengthen both the structural and mechanistic conclusions of the study. Most notably, we improved the in situ cryo-ET reconstruction to ~3.38 Å global resolution (with local resolution reaching ~2.8 Å), compared to the ~5.5 Å map in the original submission. This advance unambiguously resolves SNOR bound to dormant ribosomes in cells and firmly establishes the physiological relevance of the complex. In parallel, we now define the molecular mechanism underlying SNOR–eIF5A cooperation in ribosome reactivation through high-resolution structural analysis combined with functional characterization of ribosome-binding–deficient and C-terminal tail–deleted SNOR mutants, as well as perturbation of eIF5A activity, under endogenous regulatory control in vivo and in vitro.

Detailed, point-by-point responses to all reviewer comments are provided below.

Reviewer #1:
This is an important study that extends our understanding of how eukaryotes regulate global cellular translation in response to environmental stress. The structural and biochemical analyses are of high quality. The experiments are convincing and properly controlled. There are several aspects however, that require clarification. Specifically:

1. To assign extra density associated with hibernating ribosomes in glucose-depleted cells, the authors use fairly indirect structure-based approaches. Although there is nothing wrong with this, the mass spectrometry analysis seems a more direct way to identify the ribosome-associated protein factors. Was such analysis performed? If yes, what were the results? If no, maybe it should be done.

Response: Thank you very much for this comment. We initially did not pursue this approach based on our experience from our previous study (Gemin *et al.*, 2023), where we observed rapid dissociation of ribosome-associated factors during ribosome purification, consistent with their transient binding to ribosomes. Nevertheless, in response to the reviewer's suggestion, we performed mass spectrometry analysis on purified ribosome fractions isolated from day 1 and day 7 glucose-depleted cells. Consistent with our cryo-ET observations, SNOR was reproducibly detected in ribosome fractions from day 7 cells but was largely absent from day 1 samples. We compiled a list of hibernation and translation factors levels in ribosomes isolated from day 7 relative to day 1 of glucose depletion.

Gene name	Protein name	Normalized avg intensity (Day 7 / Day 1)
eft201	Elongation factor 2	**18.67**
**SPBC21C3.19**	**SDO1-like protein C21C3.19 (SNOR)**	**3.56**
cdb4	Curved DNA-binding protein (Ebp1 homolog)	**5.75**
SPBC16A3.08c	Oga1 / Stm1	**0.45**
tif51a	eIF5A	**0.07**

Response table 1. Normalized average protein intensities based on unbiased mass spectrometry analysis of ribosomes isolated at Day 7 relative to Day 1.

This was added as Supplementary Table 1 and the raw data is shared on https://figshare.com/s/a47eb5002eec57a1830c). We have also included the following statement:

Lines 112- 114: "Consistently, mass spectrometry analysis confirmed the presence of SNOR/Rtc3 in ribosomes isolated after prolonged glucose depletion (Supplementary Table 1)."

2. Authors discuss interaction of SNOR with and important and still enigmatic eIF5A (Fig. 4a,b). It would be really interesting to know whether SNOR and eIF5A show binding cooperativity. The established in vitro binding assay described in the paper (Figure 2c) could answer this question.

Response: This is an excellent point. In the revised manuscript, we performed the requested in vitro binding assay, examining SNOR and eIF5A association with ribosomes individually and in combination. When both factors were present, we observed increased recovery of each in the ribosomal pellet compared with either factor alone, consistent with cooperative binding to the 60S subunit. These findings provide a mechanistic basis for the functional interplay between SNOR and eIF5A during ribosome restart.

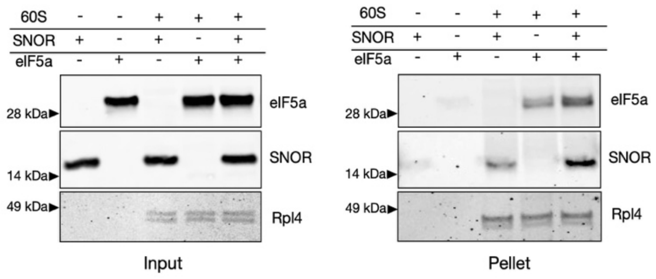

Response Figure 1. Immunoblot analysis of ribosome co-sedimentation assay showing binding cooperativity between SNOR and eIF5A with purified *S. pombe* 60S large ribosome subunit. SNOR and eIF5A were detected via His-tag; Rpl4 served as a loading control.

The new data are presented in the Revised Figure 4 and is also described in the revised Results section, lines 223-229:

"To test whether SNOR and eIF5A influence each other's ribosome association, we performed co-sedimentation assays using purified 60S subunits together with SNOR and eIF5A (Fig. 4e). When incubated individually, each factor co-pelleted with the 60S subunit; however, when added together, recovery of both SNOR and eIF5A in the ribosomal pellet increased compared with either factor alone. This enhanced co-pelleting is consistent with cooperative binding between SNOR and eIF5A mediated by this interface."

3. Cessation of translation may lead to degradation of rRNA in the dormant ribosome and one of the proposed roles of hibernation factors is protection from such degradation. It would be a simple but informative experiment to compare the rRNA integrity in the wt and SNOR-depleted cells over time during cessation of translation caused by glucose depletion.

Response: We have performed RNA extraction from WT and SNOR KO cells and ran an agarose gel to assess integrity of the rRNA. In our glucose-depletion time course, total RNA profiles from WT and ΔSNOR cells showed comparable 28S and 18S rRNA integrity over time, with no evidence for gross rRNA destabilization in the absence of SNOR under these conditions.

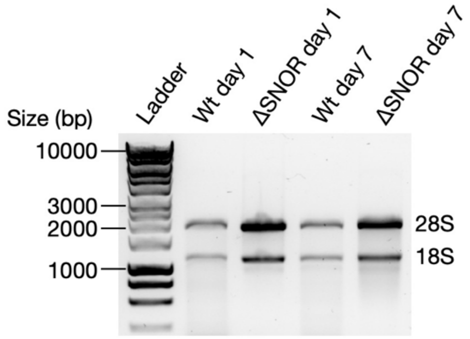

Response Figure 2. rRNA integrity during glucose depletion in WT and ΔSNOR cells. Total RNA was isolated from wildtype (WT) and ΔSNOR S. pombe cells at the indicated time points during glucose depletion and resolved on a 1% agarose gel stained with SYBR Safe. HyperLadder™ 1kb DNA ladder was included as molecular weight marker. The positions of the 28S and 18S rRNAs are indicated.

Minor points:

1. 142-143. Fig. 2a does not seem to support the statement that "rtc3 transcript upregulation being the most prominent among the analyzed hibernation factors" . Upregulation of eft201 and possibly of tif51 seem to be somewhat more pronounced than that of rtc3.

Response: We have revised the text to correct this inaccuracy.

Lines 136-139: "Transcripts for the genes eft201, tif51, and rtc3 encoding eEF2, eIF5A, and SNOR, respectively, were all elevated after 3 days of glucose deprivation, with eft201 and tif51 transcripts showing the strongest induction, whereas oga1 (Stm1) transcript levels remained largely unchanged."

2. 152-153 and Fig. 2c. Why is the small subunit protein present in the 60S sample?

Response: In the initial experiment, a residual of SSU signal remained after puromycin-mediated splitting followed by high salt treatment due to residual 80S contamination that did not fully dissociate. We have repeated the splitting procedure to effectively eliminate detectable 80S ribosomes. We then repeated the binding experiment using these improved 60S preparations (please see **Response Figure 3)**. The Panel in revised figure 2 in the main text has been updated.

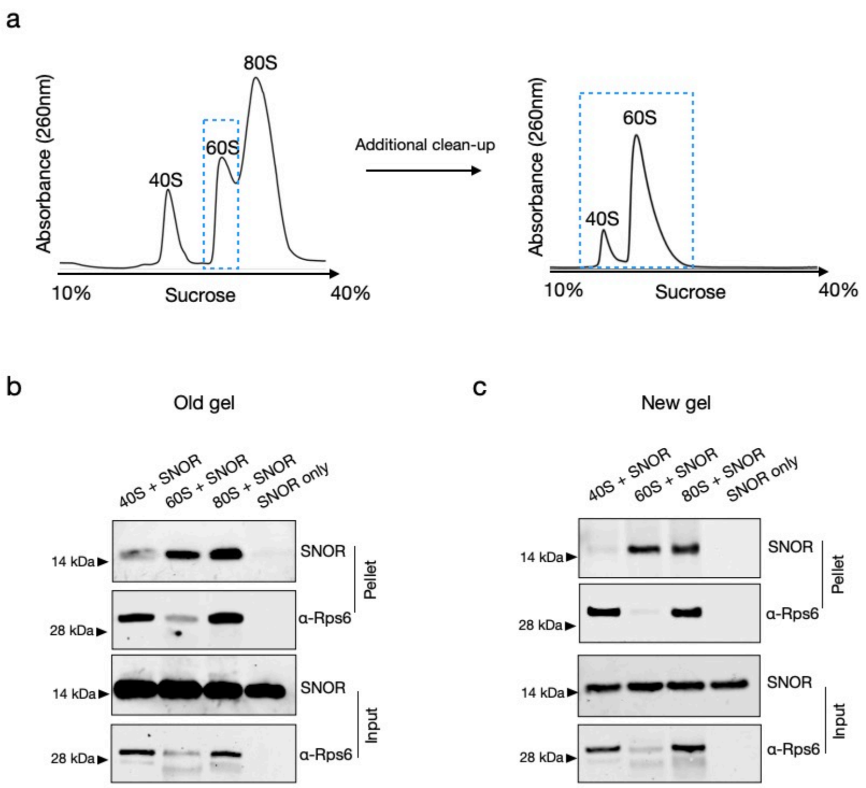

Response Figure 3. (a) Absorbance profile obtained after splitting of *S. pombe* isolated crude ribosome sample (left) and an additional clean-up step (right) on the isolated large ribosomal subunit fractions (blue dotted box). (b) Immunoblot analysis of SNOR binding to 40S, 60S, and 80S ribosomal subunits using co-sedimentation assays. SNOR was detected via His-tag; Rps6 was used as a loading control. (c) Immunoblot analysis of SNOR binding to 40S, 60S, and 80S ribosomal subunits using co-sedimentation assays, following the additional 60S clean-up step. SNOR was detected via His-tag; Rps6 was used as a loading control.

3. 234-240 and Fig. 4g. While overexpression of SNOR does affect translation, it remains unclear whether this effect is related to the ability of SNOR to bind to the ribosome. Therefore, this experiment lacks the critical control: the effect of the wt SNOR overexpression should be contrasted with that of the inactive SNOR mutant (e.g. K68E/H96A/R97E).

Response: Due to the high expression levels achieved in this overexpression system, we could not reliably control SNOR abundance relative to its ribosome-binding affinity, raising the possibility that even binding-deficient mutants may engage ribosomes at supraphysiological levels. Because this assay does not provide

a physiologically controlled assessment of SNOR function and is not central to our main conclusions, we have removed the experiment and the corresponding figure panel. We instead focus on loss-of-function and endogenous knock-in ribosome-binding and C-terminal tail mutants under native regulatory control, which provide more rigorous and physiologically relevant evidence that SNOR ribosome binding is required for efficient translation restart following glucose depletion.

4. 248-256. Extended Data Fig. 10 seems unneeded given that the main result is shown in the main Fig. 5b.

Response: We have moved the replicate profiles to Supplementary Information.

5. 328-329. It is unclear what authors mean by "potential nascent chain stuck in the tunnel of hibernating ribosome". Do they mean tRNA-unlinked nascent chain? Because if nascent chain remains linked to the tRNA, then SNOR should not be able to bind.

Response: Correct, we refer to a tRNA-unlinked nascent polypeptide retained within the exit tunnel of a hibernating ribosome. We have revised the text to explicitly state this and to remove any ambiguity.

The revised text now reads line 312-314: "*The positioning of the SNOR C-terminal tail within the exit tunnel raises the possibility that SNOR senses nascent polypeptides retained in hibernating ribosomes prior to translation restart.*"

Reviewer #2

The structural and biochemical data are of high quality and convincingly support the conclusion that SNOR associates with hibernating 80S ribosomes upon glucose depletion. The evidence indicates that SNOR itself is not a primary driver of ribosome hibernation since its overexpression alone has only a modest translational effect. Interestingly, SNOR KO cells still undergo hibernation normally—but the recovery from glucose starvation is impacted. This interpretation is well supported by the data.

The study also extends previous observations of eEF2–Stm1(Oga1)-containing hibernating complexes and connects them to a newly identified ribosome-bound factor. Interestingly, a closely related inactive 80S complex has been reported in Xenopus oocytes (80S containing eEF2, Habp4/Stm1, eIF5A, and Dap1b; ref. 21). Although Dap1b is not homologous to SNOR, it appears functionally analogous, interacting with eIF5A at the PTC/PET. Perhaps the real novelty in the current study lies in the detailed description of the L1-stalk–eIF5A–SNOR bridge, which provides a mechanistic rationale for the stabilization of the L1 stalk in the inactive 80S state.

1. The authors suggest that eIF5A might be involved, but no direct evidence is presented. Given that SNOR function appears specific to glucose levels in fungi, it would be important to elucidate how SNOR connects to mitochondrial metabolism or the signaling events governing ribosome reactivation.

We thank the reviewer for highlighting the potential involvement of eIF5A. Although SNOR induction is triggered by carbon-source depletion, our data indicate that its function is not restricted to mitochondrial-tethered ribosomes, as SNOR associates broadly with dormant ribosomes under these conditions. We agree that the key mechanistic advance lies in defining how SNOR contributes to translational restart, particularly through its functional coupling to eIF5A. To address this directly, we performed additional experiments. First, we show that SNOR and eIF5A bind cooperatively to the ribosome, with increased recovery of both factors when present together (see Reviewer 1, point 2). Second, our improved in situ cryo-ET reconstruction resolves the hypusinated loop of eIF5A engaging the C-terminal tail of SNOR, providing a structural basis for their cooperation during ribosome reactivation (Response Fig. 4a). Third, inhibition of

eIF5A hypusination using the spermidine analogue N[1]-guanyl-1,7-diaminoheptane (GC7; PMID 33731685) significantly impaired translational restart following glucose depletion and exacerbated the restart defect in SNOR knockout cells. Consistent with this, GC7 treatment reduced viability after glucose reintroduction.

Together, these data identify the mechanism underlying the cooperative role for SNOR and hypusinated eIF5A in promoting efficient ribosome reactivation following glucose starvation.

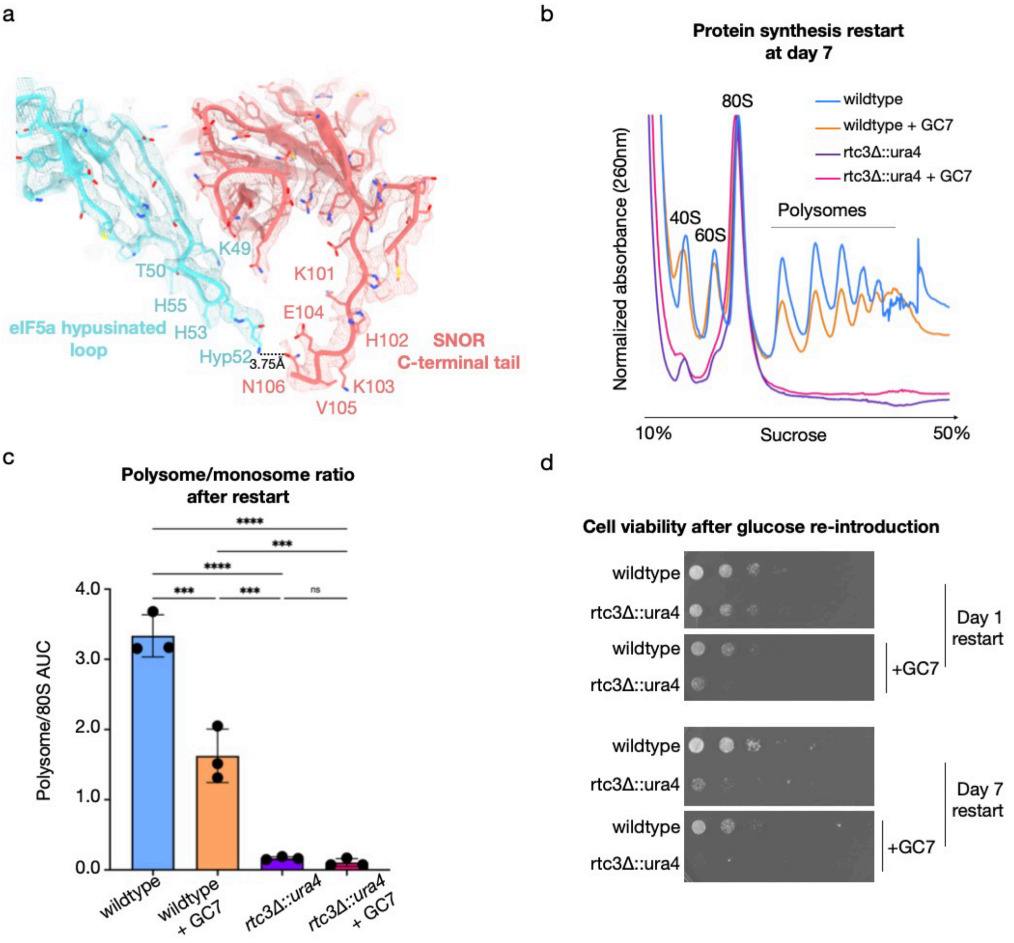

Response Figure 4. SNOR–eIF5A interaction and requirement of hypusination for translation restart. (a) (a) Cryo-ET density (mesh) highlighting eIF5A and SNOR. The interaction between hypusinated eIF5A Lys52 (Hyp52) and SNOR Asn108 is indicated. Atomic models are shown as sticks and cartoons.(b) Representative polysome profiles from S. pombe cells after 7 days of glucose depletion followed by 2 h glucose readdition, with or without 20 µM GC7. Profiles were normalized to the 80S peak (WT −GC7, blue; WT +GC7, orange; rtc3Δ::ura4 −GC7, purple; rtc3Δ::ura4 +GC7, magenta).(c) Polysome-to-monosome ratios quantified from (b) (mean ± S.D.; t-test, **P < 0.001).(d) Serial dilution growth assays of WT and rtc3Δ::ura4 cells grown with or without 20 µM GC7.

The results from the restart assays using the GC7 inhibitor are now incorporated in the revised Figure 5 in the updated text and the results sections titled “SNOR cooperates with eIF5A to promote protein synthesis restart following cellular dormancy”, lines 274-287.

2. Gene expression analysis: Lines 140–143 state that eft201, tif51, oga1, and rtc3 transcripts are all elevated upon glucose depletion. However, the qPCR data in Fig. 2a do not appear to show a corresponding increase in Oga1 expression. This discrepancy should be clarified.

Response: We thank the reviewer for raising this important point. Oga1/Stm1 is a relatively abundant ribosome-associated factor, and its transcript levels show only modest changes upon glucose depletion compared to lower-abundance factors such as SNOR. This is also consistent with recent studies (PMID: 41315256; PMID: 40315826) that show that Stm1 is regulated at the level of ribosome association rather than through strong transcriptional induction. Please also refer to Reviewer 1, minor point 1 response.

3. Overexpression assay (Fig. 4g): SNOR overexpression was examined after three days in 0.5% glucose medium, just before hibernation occurs, yielding only a modest reduction in polysomes. Extending the incubation (> 4 days) and testing whether overexpression accelerates or enhances hibernation would strengthen the interpretation. Including the 60S-binding mutant in this assay would also be informative.

Response: We agree that extending the overexpression assay could, in principle, be informative. However, as discussed in our response to Reviewer 1, Minor Point 3, overexpression does not allow reliable control of SNOR abundance relative to ribosome-binding affinity and may lead to non-physiological ribosome engagement. Moreover, prolonged low-glucose conditions (>4 days) result in near-complete shutdown of global translation (PMID: 39379376), limiting the interpretability of such assays. We therefore removed the overexpression experiment and instead focused on loss-of-function and endogenous knock-in ribosome-binding and tail mutants, which provide a more physiologically relevant and mechanistically rigorous assessment of SNOR's role in translation restart.

4. Recovery assays (Fig. 5b,d): In addition to the SNOR-KO strain, it would be valuable to test the 60S-binding mutant for both polysome recovery and growth rescue, to directly link the structural findings to functional consequences.

Response: Thank you for this suggestion, we have extended the recovery assays to include knock-in strains for both a ribosome-binding–deficient SNOR (K68E/H96A/R97E) mutant in addition to the SNOR C-terminal tail (G100STOP) mutants. In both polysome recovery and growth rescue assays, these mutants phenocopy the SNOR knockout, demonstrating that ribosome association and tail insertion are essential for SNOR function during recovery from glucose depletion, which validate our structural observations and support a role for SNOR tail and binding to the ribosome in restart of protein synthesis.

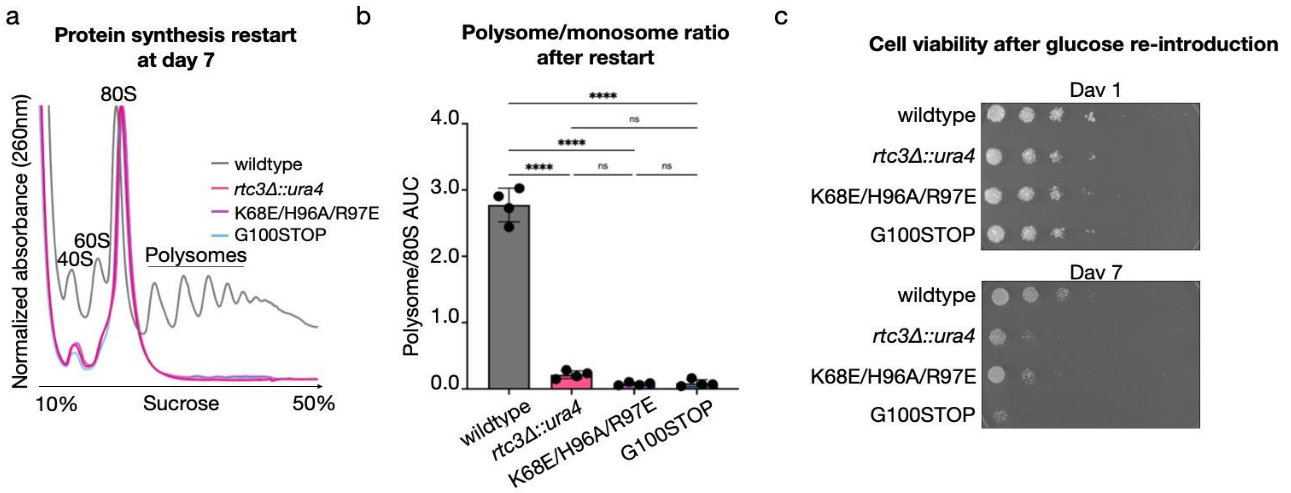

Response figure 5. (a) Overlay of representative polysome gradient profiles from *S. pombe* cells after reintroduction of glucose for 2 hours following 7 days of glucose depletion. Absorbance values were normalized to 80S peak for each sample. Profile obtained from wildtype cells shown in gray, profiles obtained from *rtc3Δ::ura4* cells shown in pink, profiles obtained from rtc3-K68E/H96A/R97E cells shown in purple and profiles obtained from rtc3-G100STOP cells shown in blue. (b) Quantification of polysome to monosome area under the

curve ratios (panel a). Data shown as mean ± S.D. Statistical analysis was performed using one-way ANOVA with Tukey's post-hoc multiple comparisons test. ****P < 0.0001. (c) Serial dilution spot assays of cultures grown to saturation in EMM with 0.5% glucose at 32˚C and then continuously incubated in the same media. 10-fold serial dilutions of the normalized samples at OD_{595} of 0.25 were spotted on EMM plates containing 2% glucose and incubated at 32˚C. Day 1 corresponds to the 24 h time point of continuous incubation.

The results for this experiment are presented in Fig. 5 (revised) and Extended Data Fig. 9.

Reviewer #3

In this study, Gluc et al. have identified a new ribosomal factor, SNOR, along with the hibernating factors Oga1 eEF2 and eIF5A, using cellular tomography (cryo-ET) of glucose-starved S. pombe cells. Also reported is a single-particle cryo-reconstruction in vitro reconstitution of the 60S ribosome SNOR complex. Furthermore, SNOR functional characterization is carried out by creating SNOR knockout and point mutants, as well as SNOR overexpression.

The authors have claimed that the SNOR has a role in translation restart following cellular dormancy. The SNOR binding to glucose-stressed ribosomes appears apparent, but the mechanism of ribosome reactivation is not clearly demonstrated at the molecular level.

1. The Cryo-ET reconstruction is claimed to be 5.5 Å, and the single particle reconstruction is claimed to be 2.9 Å. However, the cryo density claimed for SNOR is fragmented in both Cryo-ET reconstruction and single-particle reconstruction. Overall map quality is poor; a few screenshots are shown in Figure 1. attached with comments. At a 2.9 Å resolution, we should be able to see the bulky side-chain residues clearly. None of the interactions between SNOR-60S ribosome, shown in figures, are supported with cryo-EM densities. SNOR has low occupancy, as evident in cryo-EM reconstruction; only ~10% of particles from 2D classification were finally selected for the 60S-SNOR reconstruction. The FSC shows in the PDB validation report for 60S-SNOR reconstruction is also falling very early. I suggest improving the occupancy of SNOR in the final reconstruction by increasing the SNOR concentration in the in-vitro reconstruction. Sorting the structural heterogeneity by extensive 3D classification and using partial signal subtraction embedded in RELION.

Response: We thank the reviewer for this careful evaluation and for the constructive technical and mechanistic suggestions. We have substantially expanded and reprocessed both the in situ cryo-ET and in vitro cryo-EM datasets to address concerns regarding map quality, SNOR occupancy, and model support.
We recollected cryo-ET data using an expanded acquisition strategy, acquiring 1,012 tilt series from 26 lamellae (approximately ten-fold more data than in the original submission), yielding nearly 100,000 ribosomes from high-quality tomograms. This resulted in a final in situ reconstruction at ~3.38 Å overall resolution, with local resolution reaching ~2.8 Å in the SNOR region (**Response Figure 6**). The improved map resolves SNOR with clear side-chain density at the peptidyl transferase center and visualizes the interaction between the SNOR C-terminal tail and the hypusinated loop of eIF5A, defining the structural interface underlying ribosome reactivation. Together with functional analyses of SNOR ribosome-binding mutants and inhibition of eIF5A hypusination (see Responses to Reviewer 2, points 1 and 4), these data establish the molecular mechanism by which SNOR and eIF5A cooperate to promote translation restart following glucose depletion.
Second, we reprocessed the single-particle cryo-EM dataset in RELION, incorporating additional rounds of 3D classification and focused refinement with partial signal subtraction to address structural heterogeneity, as suggested (please see **Response Figure 7**). These refinements improved SNOR density in the 60S–SNOR complex, with corresponding improvements in validation metrics, including FSC behavior and map-to-model agreement.

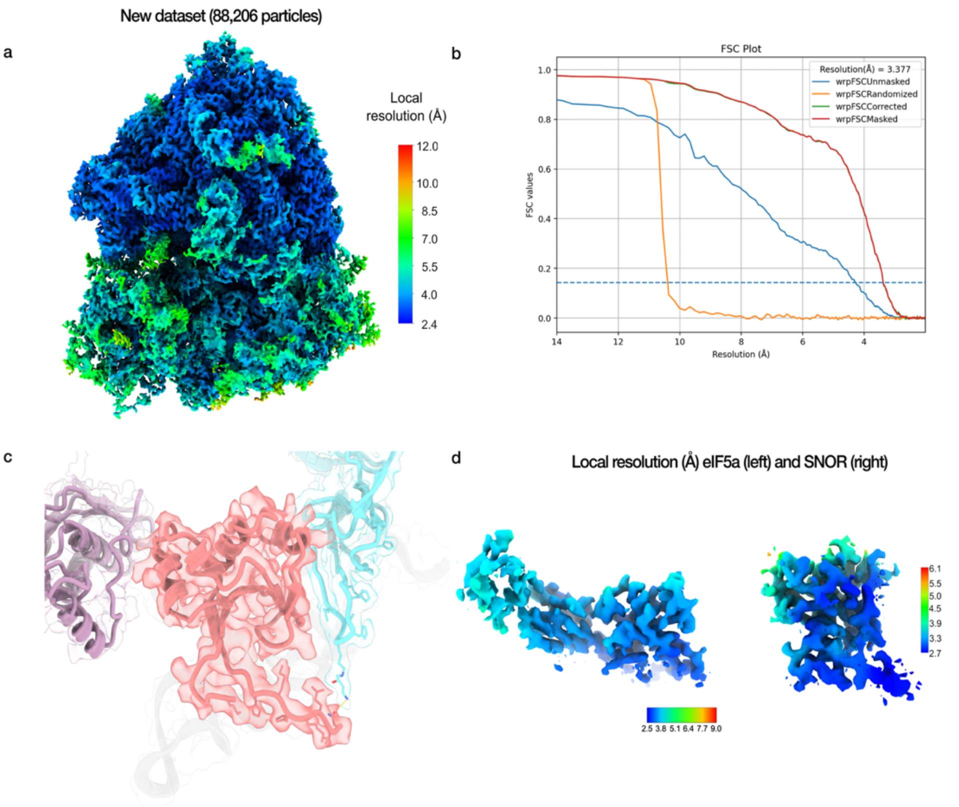

Response Figure 6. Improved in situ cryo-ET reconstruction resolves SNOR at side-chain level and shows a PTM *in situ*. (a) Local resolution map of the revised in situ cryo-ET reconstruction of the hibernating ribosome obtained from the expanded dataset (95,485 particles). (b) Gold-standard FSC curve of the final cryo-ET reconstruction, yielding a global resolution of 3.38 Å (FSC = 0.143). (c) Overview of the SNOR binding site on the 60S subunit in the revised map. SNOR in salmon; eIF5A, including hypusine loop, in cyan and eEF2 in purple. (d) Local resolution estimate for SNOR, indicating resolutions up to 2.8 Å in the best-resolved regions, consistent with clear side-chain density and supporting the SNOR–eIF5A interaction described in the manuscript.

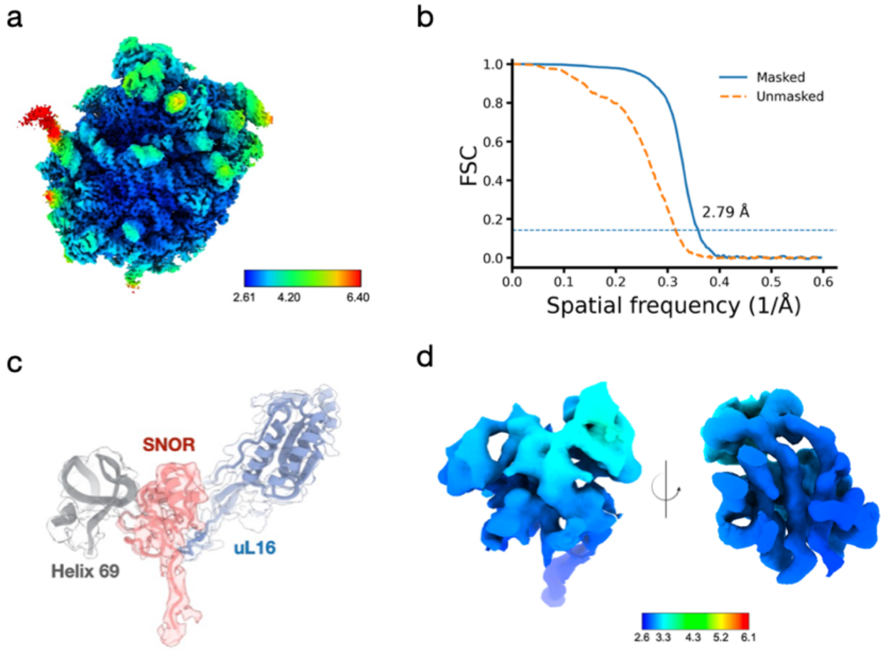

Response Figure 7. Improved cryo-EM reconstruction resolves SNOR at side-chain level. (a) Local resolution map of the revised cryo-EM reconstruction of the 60S:SNOR reconstituted complex in vitro (b) Gold-standard FSC curve of the final cryo-EM reconstruction, yielding a global resolution of 2.8 Å (FSC = 0.143). (c) Overview of the SNOR binding site on the 60S subunit in the revised map (SNOR in coral; uL16 in blue, H69 in gray). (d) Local resolution estimate for SNOR, indicating resolutions up to ~3 Å in the best-resolved regions.

The revised maps, models, FSC curves, and validation metrics are presented in the updated figures and tables.

2.	An interesting observation in Gemin et al. 2024 was that H69 adopts a different conformation in the hibernating ribosome. However, in the current manuscript, the authors do not mention this. A comparison of the current coordinates with the 11 Å cryo-ET reconstruction (PDB ID: 9AXU) is shown in the figure below. It looks like the altered conformation reported earlier overlaps with the SNOR. Shown in Figure 2, attached with comments. Any explanation?

Response: Thank you very much for this comment. The altered conformation of H69 in hibernating ribosomes was indeed described in our previous study (Gemin *et al.*, 2024) and was briefly mentioned in the initial manuscript. Nevertheless, we agree that this point now merits more explicit discussion. In the revised manuscript, we now elaborate on this comparison and include a direct structural overlay between the single-particle reconstructions representing inactive and translating states (PDB IDs: 9AXT/9AXU and 9AXV respectively) and the current higher-resolution map to compare the shift in H69 (**9RVU – this study**). This comparison indicates how the presence of SNOR binding could maintain H69 in the outward conformation and how its loss in dormant ribosomes could lead to the conformational change in this helix we observed before **(Response Fig. 8)**.

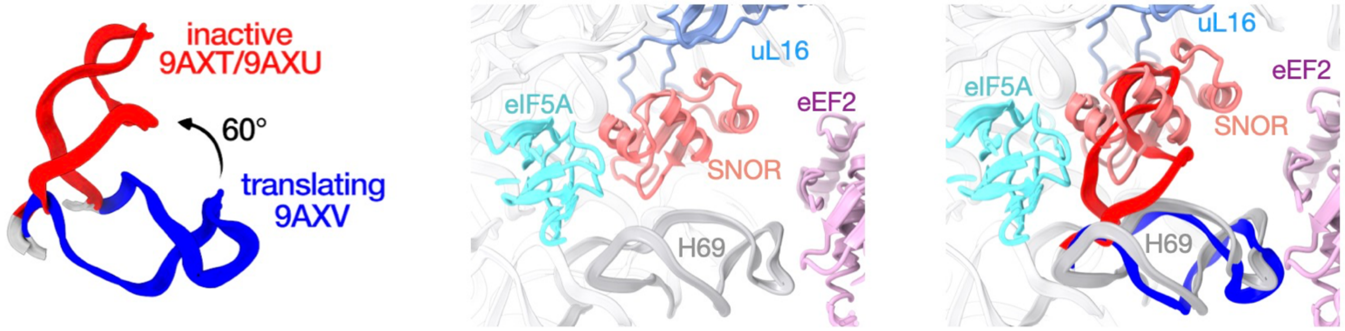

Response Fig. 8. SNOR stabilizes H69 in an outward conformation. Comparison of H69 conformations in inactive (PDB: 9AXT/9AXU; red), translating (PDB: 9AXV; blue), and SNOR-bound hibernating ribosomes (9RVU; grey). Superposition in two views shows that SNOR binding maintains H69 in an outward conformation, whereas the inward conformation observed in the absence of SNOR would sterically clash with the SNOR binding site.

This figure has been added as Supplementary Figure 4 in the revised manuscript and details about this interaction is included in results section "SNOR forms a tripartite complex with uL1 and eIF5A on the ribosome" and also in the discussion.

Lines 218-221: *"The position occupied by SNOR also overlaps with the inward-shifted conformation of helix H69 previously observed in inactive ribosomes isolated under similar conditions, indicating that SNOR binding may stabilize the conformation of this helix and sterically occludes this inactive H69 configuration (Supplementary Fig. 4)".*

And also in the discussion line 315-319: *"This configuration also stabilizes helix H69 in an outward orientation and prevents adoption of the inactive conformation previously observed under similar stress conditions*[25]*. By maintaining the structural integrity of the PTC region, SNOR preserves ribosomes in a restart-competent configuration."*

3.	As SNOR and some other factors were missed at 11 Å cryo-ET reconstruction, what are the probabilities of missing some small protein in the current 5.5 Å resolution cryo-ET reconstruction? I would suggest performing mass spectrometry to determine if any unknown factors remain unmodeled in

cryo-ET maps. That would also confirm that it's SNOR, not another protein having the same domain with disordered regions.

Response: We thank the reviewer for this important point. As described in our response to Reviewer 1, point 1, we performed mass spectrometry analysis of purified ribosome fractions isolated from glucose-depleted cells, which independently confirmed the enrichment of SNOR/Rtc3 in dormant ribosomes. Most importantly, the substantially improved in situ cryo-ET reconstruction (3.38 Å overall resolution, with ~2.8 Å local resolution in the SNOR region) now resolves continuous density corresponding to the SBDS core fold and side-chain features that unambiguously match SNOR. The structural identity of the factor occupying this site is therefore directly established by the cryo-ET map itself.

Interestingly, we did detect Cdb4 (homolog of human Ebp1) in the mass spectrometry dataset. When the in situ cryo-ET map is low-pass filtered to ~7 Å, a weak density is visible near the polypeptide exit tunnel that may be consistent with this factor (**Response Figure 9**). However, due to its low local resolution and lack of high-resolution features, we conservatively refrained from modeling this density in the final atomic structure. Together, these analyses indicate that SNOR is the principal factor occupying the resolved density, while any additional factors, if present, are likely of lower occupancy.

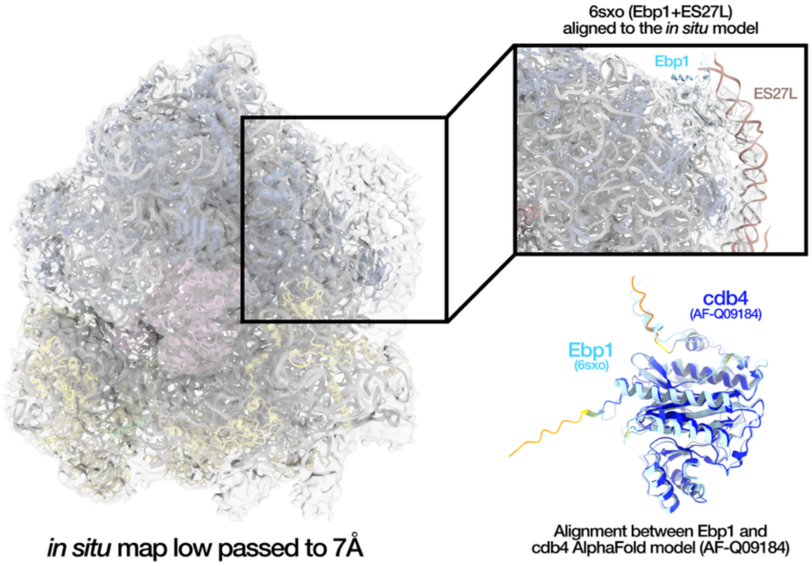

Response Figure 9. Putative density for cdb4 at the ribosomal exit tunnel revealed at low-res. Top left: *In situ* cryo-ET map low-pass filtered to 7 Å, highlighting a weak additional density near the exit of the polypeptide exit tunnel. Inset shows a close-up of the fitting of the Ebp1 and ES27L (6SXO) into the low-pass filtered density. Bottom right panel: Structural alignment between Ebp1 and the cdb4 AlphaFold model.

4. The SNOR name is coined because it resembles Domaine 1 of the SBOD protein. If we look carefully, the polarity of SNOR and SBOD DI is opposite. SNOR's C-terminus is inserted into the PTC, whereas the N-terminus of SBOD. Basically, the secondary structure topology will be opposite. Whether this claim same domain is valid? It needs to be supported with literature. Furthermore, does the sequence analysis shown in Figure 3d have any relevance? As the direction of SNOR and SBOD D1 is opposite.

Response: We thank the reviewer for raising this point. To clarify, SNOR (Rtc3/YHR087W) is a bona fide member of the SBDS protein family, as established in prior studies (PMID: 1570163; PMID: 22720784; reviewed in PMID: 28942353) and annotated in PomBase (SPBC21C3.19) and the Saccharomyces Genome Database. Structurally, SNOR adopts the conserved SBDS core fold with the same domain polarity and secondary-structure topology as canonical SBDS (**see Response Figures. 11–12**). In particular, there is no inversion of the SBDS core. The distinction lies in ribosome engagement: SNOR contains a SNOR-specific C-terminal extension that inserts into the peptidyl transferase center, resulting in a different overall orientation on the mature ribosome. The apparent discrepancy arises because the termini of SNOR and Sdo1/SBDS are positioned proximally in the ribosome-bound state, which may give the impression of reversed polarity (Response Figure 12). Accordingly, the sequence alignment in Fig. 3d remains directly

relevant, as it highlights conservation within the shared SBDS core domain rather than the terminal extensions.

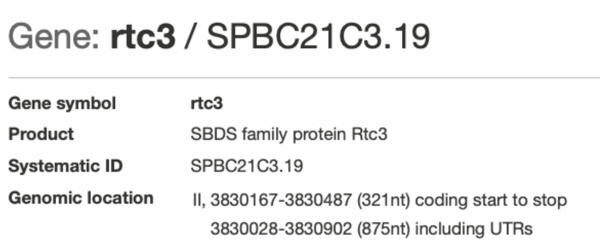
Gene: **rtc3** / SPBC21C3.19

**Gene symbol**	rtc3
**Product**	SBDS family protein Rtc3
**Systematic ID**	SPBC21C3.19
**Genomic location**	II, 3830167-3830487 (321nt) coding start to stop 3830028-3830902 (875nt) including UTRs

Response Figure 11. Screenshot of the PomBase gene annotation for rtc3 (SPBC21C3.19), indicating its classification as an SBDS family protein (product: "SBDS family protein Rtc3"). This annotation is consistent with prior literature and supports the designation of SNOR as an SBDS-containing protein.

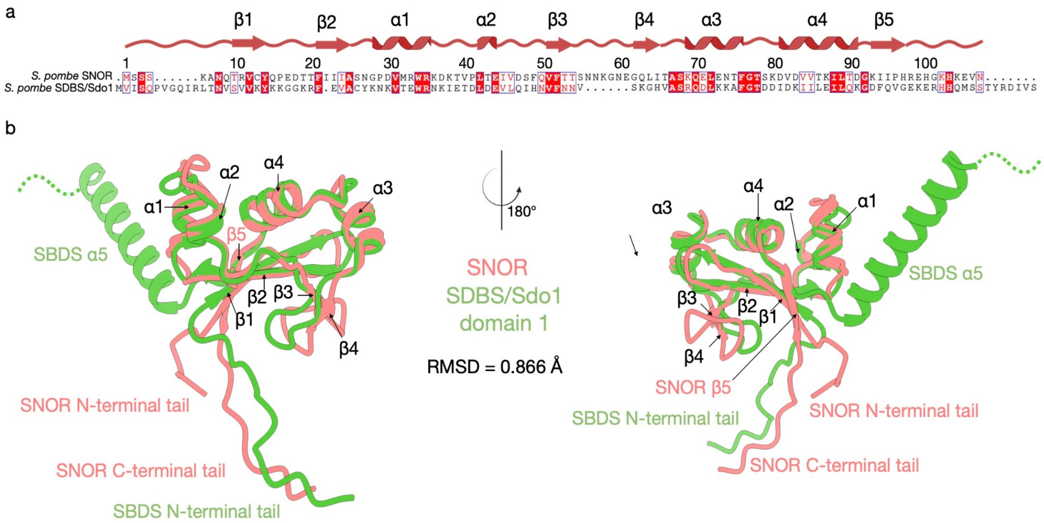

Response Figure 12. (a) Schematic representation of SNOR secondary structure elements (top). β-sheets represented as arrows and symbol "β". Helices represented as ribbons and symbol "α". Sequence alignment of *S. pombe* SNOR with *S. pombe* SBDS/Sdo1 (bottom). Alignment generated using ESPript 3.0. (b) Structural comparison of a superimposed AlphaFold model of *S. pombe* "SBDS domain" of Sdo1 (human SBDS homolog) (green) and SNOR (coral) atomic model.

We have clarified this point in the revised manuscript by consistently referring to SNOR as an "SBDS-domain-containing" protein and by adding Extended Data Figure 7, which presents a structural overlay highlighting conservation of the SBDS core fold despite differences in ribosome engagement. Accordingly, the sequence alignment shown in Fig. 3 remains relevant, as it emphasizes conservation within the shared SBDS core domain, including residues that mediate ribosome interactions.

5. The authors have made the SNOR knock-out strain. However, ideally, I would like to create an SNOR complement strain in an SNOR knockout background and conduct all functional studies.

Response: We thank the reviewer for this suggestion. In the revised manuscript, we addressed this point by generating SNOR knock-in strains in the SNOR knockout background, reintroducing either wildtype or mutant SNOR alleles at the endogenous locus under native regulatory control. These strains are genetically equivalent to the parental wildtype strain, differing only at the engineered SNOR allele. This strategy provides a physiologically controlled complementation approach under native regulatory conditions and avoids confounding effects associated with ectopic overexpression. Accordingly, the wildtype strain serves as the appropriate complementation control for both the SNOR knockout and knock-in mutants in all glucose restart assays.

6. SNOR is expressed in glucose concentrations of 0.5% and 20%. Whether SNOR expression is glucose-specific or 20% glucose induces osmotic stress to the cell, remains unclear. I would suggest the SNOR expression by replacing 20% glucose with an equimolar concentration of sorbitol, NaCl, etc.

Response: We tested whether SNOR induction reflects a general osmotic stress response by exposing cells to high NaCl concentrations under otherwise identical conditions. In contrast to high-glucose treatment, NaCl did not induce SNOR expression (**Response Figure 13**), indicating that SNOR upregulation is not a generic osmotic stress response. These data support the interpretation that SNOR regulation is linked to carbon availability rather than osmotic stress.

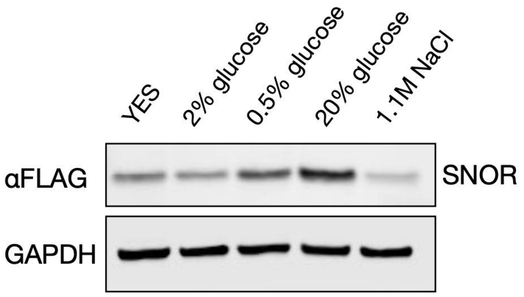

Response Figure 13. SNOR expression is regulated by carbon source availability and not by osmotic stress. Immunoblot analysis of FLAG-tagged SNOR expression in S. pombe cells grown under the indicated conditions: standard YES medium, 2% glucose, 0.5% glucose, 20% glucose, or 1.1 M NaCl. SNOR levels were detected using an anti-FLAG antibody. GAPDH serves as a loading control.

The text has been revised accordingly, and the updated Figure 2 is presented in the Results section.

7. All figure qualities can be improved.

Response: We have revised all figures to improve clarity and presentation.

Minor points:

1. In line 61: Check if reference 14 is correctly cited; in my understanding, it's not the first report.

Response: We have added original reports demonstrating RNA protection by hibernation factors (PMID: 15502846; PMID: 15278243).

2. The result section from lines 86 to 103 can be made concise, and details can be added to the method section.

Response: We have revised this section to be more concise and moved details to the Methods section.

3. Line 143: Extended data table 2 is not correctly cited.

Response: We have corrected this in the text, please note that this is now referred to as "Extended Data Table 4".

4. Paragraph 170 -176, Fig. 3C is not cited.

Response: Thank you for spotting this. Comparison of SBDS/Sdo1 and SNOR and the schematic of the tail insertion within the PTC have been moved to the updated Extended Data Fig. 7 and is now cited in the text.

5. Line 240 under nutrient stress, or it should be under carbon stress.

Response: We have corrected this in the text.

6. Extended Data Figure 1: What does M stand for? An expansion is required

Response: "M" refers to the image-processing software tool M, part of the Warp–M suite developed for high-resolution in situ cryo-EM and cryo-ET data refinement (Tegunov et al., Nat. Methods 2021). M performs reference-based refinement by jointly optimizing particle poses, local sample deformation, and electron-optical parameters. We have clarified this in the revised manuscript in the Methods section.

7. Table 1: Data collection and processing table section FSC threshold value missing

Response: This has been updated to reflect the values with new cryo-EM and cryo-ET data analyses.

8. Line 509: If the splitting buffer contains 5 mM $MgCl_2$, or lower concentration. Generally, 1 mM $MgCl_2$ is used for splitting.

Response: We used 5 mM $MgCl_2$ and not 1 mM. For eukaryotic ribosomes, we used high salt (1 M KCl) to promote subunit dissociation. A clarification has been added in the methods.

Nature manuscript 2025-08-21565A:

Referees' comments:

Referee #1 (Remarks to the Author):

In the revised manuscript, Gluc et al. have added useful details and improved further the paper. The authors have convincingly addressed most of my questions as well as, I believe, the questions and concerns of other referees.

My only remaining issue is related to the last minor point in my original review. I suspect that I am not the only one who would be confused with "nascent polypeptides retained in hibernating ribosomes prior to translation restart". I recommend that authors clarify that they mean the nascent protein unlinked from tRNA (lines 312-314) so that it clarifies that the nascent protein is unlinked from tRNA. I propose the following version of the sentence (ll. 312-314) for the authors' consideration: "The positioning of the SNOR C-terminal tail within the exit tunnel raises the possibility that SNOR senses nascent polypeptides unlinked from tRNA but retained in hibernating ribosomes prior to translation restart.

Response: We thank the referee for the positive assessment of our revised manuscript and for noting that the revisions have addressed the questions and concerns raised in the initial review. We agree that the original phrasing could be ambiguous and have revised the sentence to explicitly indicate that the nascent polypeptide is unlinked from tRNA but retained within hibernating ribosomes prior to translation restart, as suggested.

Referee #2 (Remarks to the Author):

I co-reviewed this manuscript with one of the reviewers who provided the listed reports.

Referee #3 (Remarks to the Author):

The authors have addressed my main comments in this significantly revised manuscript. In particular, I am now more convinced that the interaction between SNOR and eIF5A is important for the restart of translation following stress. This is an interesting study that moves the field forward and I support its publication.

Response: We thank the referee for recognizing the importance of the interaction between SNOR and eIF5A in translation restart following stress and for supporting the publication of our manuscript.

Referee #4 (Remarks to the Author):

The revised manuscript is very much improved. The authors have done additional experiments and analysis. Recollected cryo-ET data and improved the overall resolution to ~3.3 Å. Improved overall cryo- EM map for SNOR. Performed mass spectrometry analysis. Generated SNOR knock-out strains. Performed osmotic stress experiments, etc. Overall, the authors have satisfactorily addressed most of the points and have done a great job.

However, the authors have not mentioned anything about my query. "The authors have claimed that the SNOR has a role in translation restart following cellular dormancy. The SNOR binding to glucose-stressed ribosomes

appears apparent, but the mechanism of ribosome reactivation is not clearly demonstrated at the molecular level."

Response: We thank the referee for the positive evaluation of our revised manuscript. We also thank the reviewer for the comment regarding ribosome reactivation. Our study reveals a novel mechanism for SNOR- and eIF5A-mediated ribosome priming for translational restart following cellular dormancy. While our findings define how SNOR and eIF5A cooperate at the ribosome to enable this process, the subsequent steps underlying ribosome reactivation remain an important direction for future work.

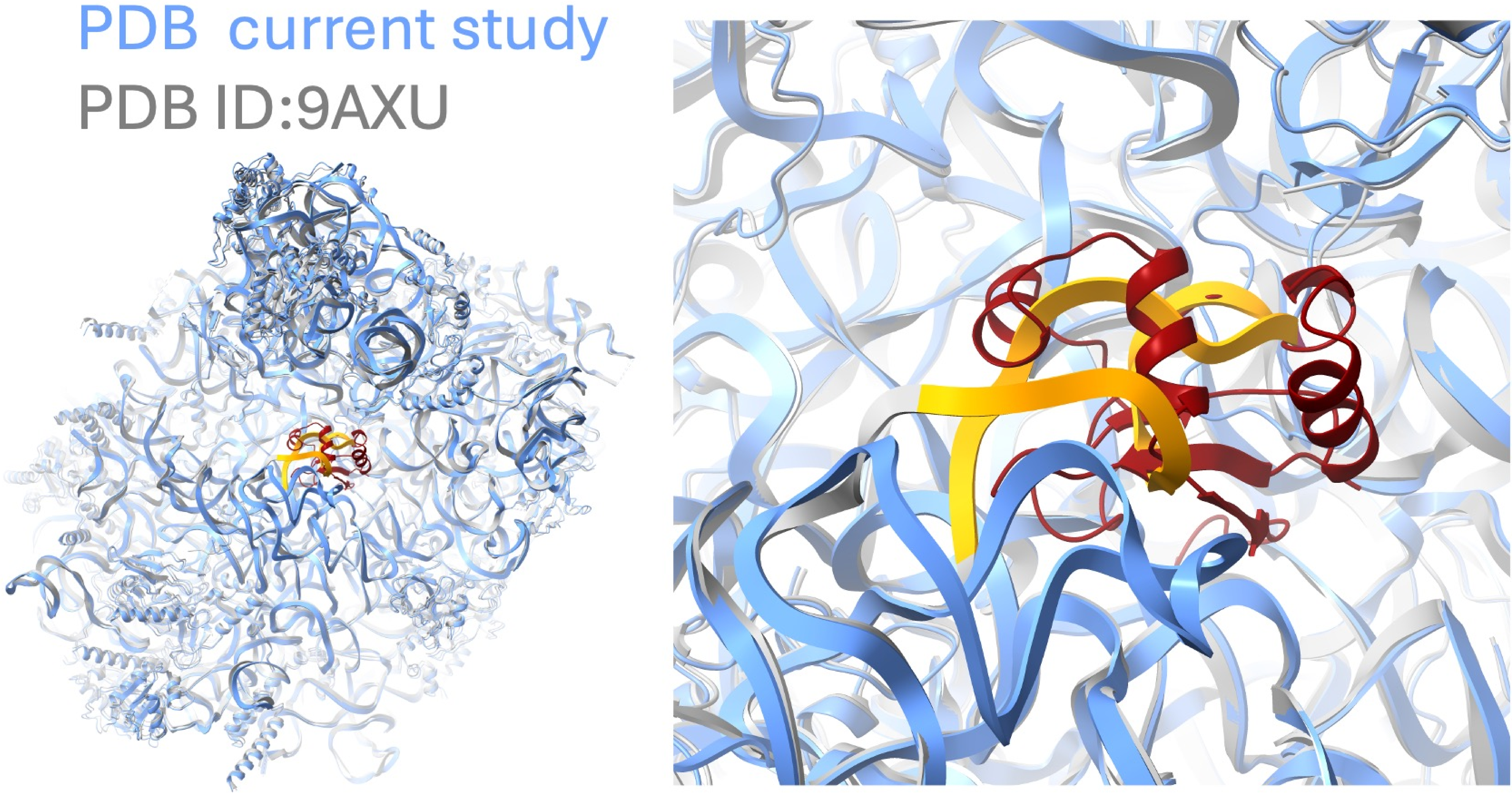
PDB current study
PDB ID:9AXU

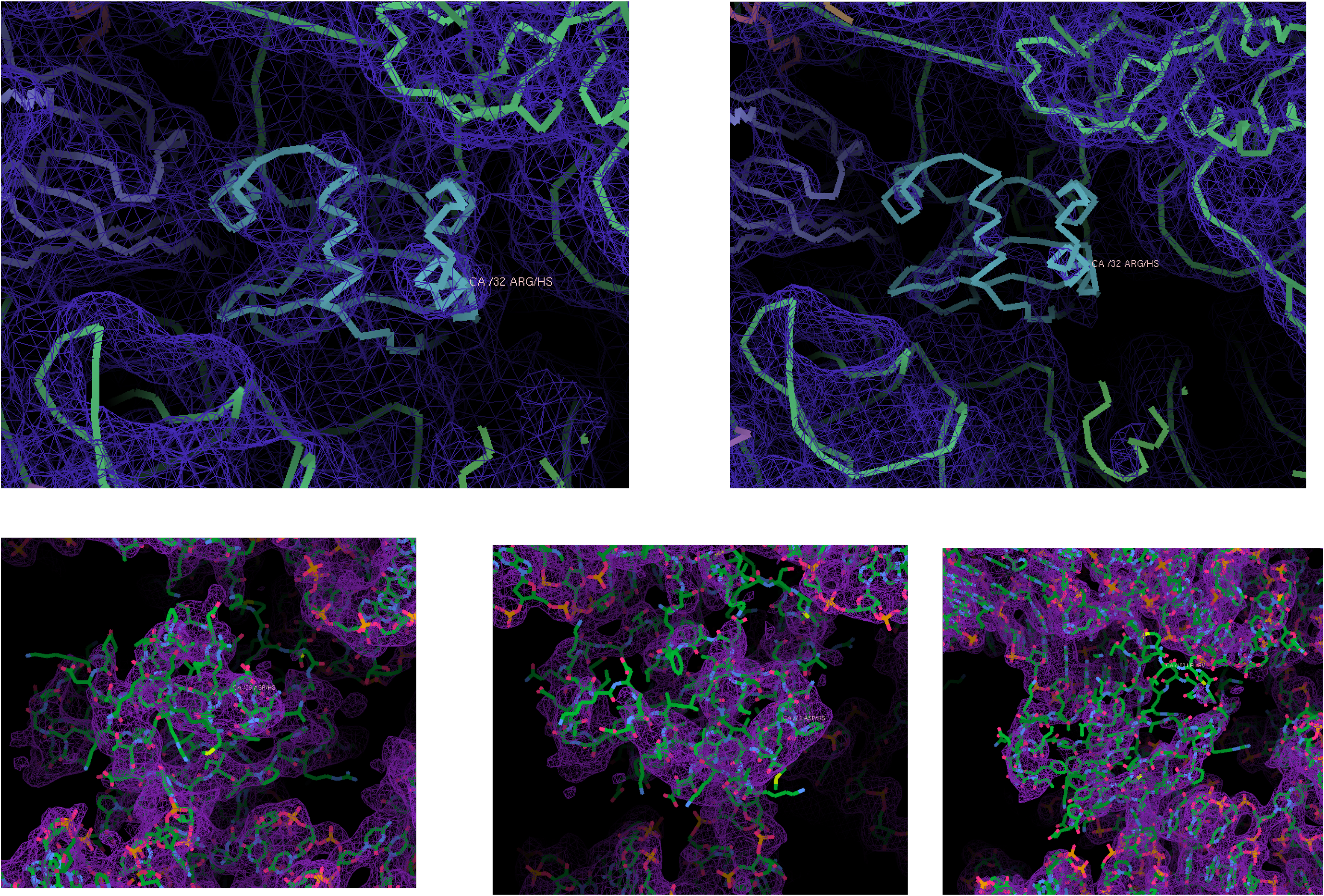
CA /32 ARG/HS
CA /32 ARG/HS
